# Identification and longitudinal assessment of sepsis phenotypes derived from routine clinical data in critically ill patients: a retrospective repeated measures latent profile analysis

**DOI:** 10.1007/s15010-025-02607-8

**Published:** 2025-07-23

**Authors:** Carolin Jung, Niklas Oetzmann, Hans-Joerg Gillmann, Thomas Stueber

**Affiliations:** Department of Anaesthesiology and Intensive Care Medicine, Hannover Medical School, Carl-Neuberg Strasse 1, 30625 Hannover, Germany

**Keywords:** Sepsis, Phenotypes, Latent profile analysis, Time stability

## Abstract

**Background:**

The definition of sepsis as an organ dysfunction with dysregulated host response leads to a considerable heterogeneity in this cohort of patients. Research is ongoing to identify subgroups of septic patients who share a common, potentially treatable pathomechanism. There are now several examples of reproducible subgroups, but they often rely on complex biomarker panels and data on their longitudinal stability are scarce, which limits their translation to the bedside. The objective of this study was to identify sepsis subgroups using routinely available clinical data and to assess the temporal stability of these subgroups.

**Methods:**

We retrospectively collected data on all adult patients treated for sepsis according to sepsis-3 criteria in our intensive care unit at a university hospital in Germany between 2013 and 2021. Subgroup identification was performed using latent profile analysis, based on data collected within 48 h of the onset of their sepsis, and was repeated with data collected 120–144 h after onset. We assessed the stability of subgroup assignment over time and the in-hospital mortality of these subgroups.

**Results:**

The analysis included 637 patients with sepsis, 83% of whom were in septic shock. Latent profile analysis of clinical data from the first period identified four subgroups with a high median probability of class membership in all subgroups and distinct characteristics. Subgroup 1 included 76 patients (12%) and was characterized by a hepatobiliary and cardiovascular dysfunction. Subgroup 2, which included 242 patients (38%), showed the least inflammation and organ dysfunction. Subgroup 3 included 236 patients (37%) and was characterized by hyperinflammation. Subgroup 4 included 83 patients (13%) who were older and had more comorbidities. They tended to have high procalcitonin and INR levels. In-hospital mortality was excessive in Subgroup 1 and lowest in Subgroups 4 and 2. In the longitudinal assessment conducted at 120–144 h post-inclusion, subgroup 1 demonstrated the greatest stability over time.

**Conclusion:**

Analysis of clinical routine data identified four distinct clinical subgroups. In the longitudinal analysis, subgroup 1, which was characterized by hepatobiliary dysfunction and high mortality, demonstrated stability over the course of the illness.

**Supplementary Information:**

The online version contains supplementary material available at 10.1007/s15010-025-02607-8.

## Background

Sepsis is defined as life-threatening organ dysfunction caused by a dysregulated host response to infection [1]. However, this definition views sepsis as a syndrome, resulting in significant heterogeneity amongst patients diagnosed and treated with sepsis. The underlying disease may be caused by a variety of different pathogens, and the severity and mechanism of organ dysfunction may depend on the individual host response and its dysregulation [2]. Despite decades of research, mortality due to sepsis and septic shock remains high [3]. The paucity of therapeutic progress may be ascribed to the complex pathophysiology of the host response and the significant heterogeneity of the cohort of patients with sepsis [2, 3]. Current research attempts to identify sepsis subgroups that may in the future make it possible to treat sepsis patients more specifically.

In addition to the obvious subtypes of sepsis based on the anatomical site of infection or the infectious agent, various research groups have identified sepsis phenotypes based on the analysis of clinical data, such as temperature time series, biomarkers, or genetics [4–9]. However, these phenotypes may often not be useful in clinical practice or sepsis research due to several factors. A common problem with the external validity of these models is overfitting, which means that the subgroups identified apply only to the training dataset, but are not reproducible when applied to different data [10]. Complex biomarker panels or genetic analyses are not readily available for daily clinical practice and take time to provide results, which poses a challenge for early trial enrolment. In a retrospective analysis of a huge clinical dataset combining data from observational and randomized trials, Seymour et al. identified and validated four new sepsis phenotypes [5]. These so-called SENECA-phenotypes correlated with host response patterns and clinical outcome. In contrast, Sinha et al. identified and validated only two distinct phenotypes, a hypo- and hyperinflammatory phenotype, in patients in the intensive care unit (ICU) with pulmonary sepsis previously included in the VALID and EARLI trials [6]. The divergent findings from these studies may be attributable to differences in patient cohorts or assessment times. Currently, only few data are available on the stability of subgroup assignments over time [11–13].

The present study aimed to identify phenotypes in critically ill patients with sepsis and septic shock based on a parsimonious set of variables derived from routinely available clinical data in the early phase of intensive care by latent profile analysis and to investigate their consistency over time. Furthermore, we aimed to compare these novel phenotypes to “traditional” phenotypes and evaluate their association with mortality.

## Methods

### Study design and population

This retrospective study was approved by the Hannover Medical School Ethics Committee, Hannover, Germany (Chairperson Prof. B Schmidt, Local Ethical Committee No. 10376_BO_SK_2022) on November 8, 2022, with a waiver of informed consent. The study was conducted in compliance with relevant guidelines and regulations. The report of this study was written in accordance with the Strengthening the Reporting of Observational Studies in Epidemiology (STROBE) guidelines [14]. The study included all adult patients treated with sepsis or septic shock in the intensive care unit of the Department of Anaesthesiology and Intensive Care, Hannover Medical School, Hannover, Germany between July 2013 and November 2021. Hannover Medical School is a tertiary university hospital. All clinically available data encompassing vital signs, laboratory data, ICU scoring systems (SOFA, SAPS-TISS) medication and physicians notes are stored in a patient data management system (m-life, Medisite GmbH, Hannover, Germany).

### Inclusion and exclusion criteria

We screened the electronic database of all patients treated in our intensive care unit between 2013 and 2021 for the diagnosis “sepsis” and “septic shock”. Identified cases were individually reviewed by an investigator to determine whether they met the criteria for sepsis according to the Sepsis-3 definition. Inclusion criteria included all adult patients with new-onset sepsis according to the Sepsis-3 definition. This included patients who were admitted to the ICU with sepsis, as well as those who subsequently developed sepsis while in the ICU. Readmissions of patients already included were excluded from the study. Death or transfer during the first 48 h did not result in exclusion from the primary analysis.

### Study endpoints and main outcome measures

The primary endpoint was to identify phenotypes in critically ill patients with sepsis at the beginning of the syndrome by latent profile analysis. We furthermore aimed to assess the time stability of these phenotypes and to compare the outcome of different sepsis phenotypes.

### Data collection

A detailed description of the data collection and pre-processing can be found in the Supplement.

### Clinical variables for phenotyping

Phenotypes were analyzed with a latent profile analysis (LPA), which belongs to the family of mixture models. Mixture models allow the identification of subgroups according to similarities in within-class parameters, as well as the assignment of cases to these subgroups based on the individual membership probability [15]. In order to assess the time-stability of the identified subgroups, a repeated measures latent profile analysis was performed. Candidate variables for phenotyping were selected based on theoretical considerations. We selected variables available in the routine clinical management of patients with sepsis representing patient characteristics, inflammation, and organ failure metrics, and allowing longitudinal assessment. We selected 17 continuous available in the electronic health database for inclusion in the latent profile analysis. We assessed the missingness, distributions, and degree of linear relationships between candidate variables. Variables with more than 10% of missing values in the selected 48-hour time frame (early time point) were excluded from the model for the LPA. As a result, IL-6, creatinine kinase and aspartate aminotransferase were excluded from the set of variables. If two variables shared a correlation > 0.5, we removed the least informative variable from the analysis [16]. This resulted in the exclusion of the Charlson Comorbidity Index in favor of age, as well as the SOFA and SAPSII scores, which are composite variables incorporating redundant information. Variables that remained in the latent profile analysis were patient age at the time of inclusion, C-reactive protein (CRP), procalcitonin (PCT), white blood cell count (WBC), platelets, bilirubin, alanine aminotransferase (ALT), creatinine, international normalized ratio of the prothrombin time (INR), serum lactate, norepinephrine dose in µg/kg/min and P/F ratio.

Missing data at the early time point were considered to be missing at random. To account for these missing data, non-parametric missing value imputation with Random Forest via the missForest [17] package was applied for variables included in the LPA. Missing values at the late time point were most often due to death or transfer to the normal ward of patients who recovered quickly and were therefore considered not missing at random. Cases with missing values of variables that were routinely available on a daily basis were therefore excluded from the dataset used for the late LPA. This included all variables selected for the early LPA except for ALT and PCT, which were not measured on a daily basis and therefore considered as including missing values at random. Missing values in the remaining dataset were imputed via missForest imputation. Variables were standardized and log-transformed, if non-normally distributed.

### Validation of subgroups

In order to validate the subgroups [18], a multivariate regression analysis was performed to identify predictor variables of subgroup assignment for both time periods (Table S8). We also analyzed changes in subgroup allocation over time. The subgroups were compared for differences in 30-day and ICU mortality. As sensitivity analyses, we performed LPA with a stepwise exclusion of variables with an above average amount of extreme values (Norepinephrine, procalcitonin, and ALT, Tables S2C-G). The optimal number of profiles was selected based on goodness-of-fit of the model with Bayesian (BIC) information criteria (lower values indicating a better fit), entropy (higher values indicating a better fit), Bootstrapped likelihood ratio test (BLRT) and class size for the retrieved profiles [16]. External validation of the model was not feasible due to the low data density of key variables in our model in publicly available databases. Therefore, we tested a reduced model in the eICU database [19] (Supplementary Tables S11A-F). The eICU database is a publicly available multi-center database sourced from the eICU Telehealth Program.

### Statistical analysis

Data management was conducted using IBM SPSS Statistics (Version 29.0.1.0 Armonk, NY: IBM Corp). The statistical analysis was performed with R statistical software (R Foundation for Statistical Computing, Vienna, Austria, Version 4.4.1). Identification of phenotypes with Latent Profile Analysis was performed with the *tidyLPA* package [20] on the High-Performance Computing (HPC) infrastructure of Hannover Medical School. To visualize transition of phenotypes on an individual patient level over time, we created Alluvial plots with the *ggalluvial* [21] and *ggplot2* [22] packages. For visualization, two alluvial plots were manually combined into a composite figure. The original alluvial plots are demonstrated in Fig. S7. The characteristics of the subgroups were visualized with chord diagrams using the *circlize* package [23]. Multivariate analyses were performed with generalized linear models using the *glm2* package. We used two-tailed testing with α set to 0.05 to determine statistical significance.

## Results

A total of 637 patients with sepsis and septic shock were identified during the investigation period (Figure S1). The median age of our cohort was 65 years. 84% percent of the patients fulfilled the Sepsis-3 criteria for septic shock. The overall cohort was characterized by a high incidence of bacteremia, and high severity of illness, with a median SOFA score of 11 (IQR 8, 13) and a median SAPSII score of 50 (IQR 43, 60). The ICU mortality was 37% (Table 1).

**Table 1:** Patient characteristics

			Subgroups		
Variable	Overall *N* = 637	1 *N* = 76	2 *N* = 242	3 *N* = 236	4 *N* = 83
Age	65 (54, 74)	62 (51, 70)	64 (53, 74)	65 (52, 77)	70 (63, 77)
Sex					
• Male	420 (66%)	47 (62%)	159 (66%)	157 (67%)	57 (69%)
• Female	217 (34%)	29 (38%)	83 (34%)	79 (33%)	26 (31%)
BMI	27 (24, 31)	28 (24, 33)	27 (24, 32)	26 (23, 31)	26 (24, 31)
*missing*	*6*		*2*	*1*	*3*
CCI	5 (3, 8)	4 (2, 7)	5 (2, 7)	5 (2, 8)	7 (5, 9)
Disease severity at inclusion					
• SOFA	11 (8, 13)	14 (10, 17)	10 (7, 12)	12 (9, 15)	9 (7, 12)
• SAPS II	50 (43, 60)	62 (51, 71)	47 (37, 54)	53 (44, 63)	51 (45, 57)
*missing*	*40*	*9*	*5*	*25*	*1*
Focus					
• Pulmonary	279 (44%)	32 (42%)	132 (55%)	84 (36%)	31 (37%)
• Gastrointestinal	58 (9%)	15 (20%)	16 (7%)	19 (8%)	8 (10%)
• Urogenital	138 (22%)	10 (13%)	36 (15%)	65 (28%)	27 (33%)
• Cardiovascular	102 (16%)	18 (24%)	34 (14%)	35 (15%)	15 (18%)
• Tissue	57 (9%)	3 (4%)	22 (9%)	28 (12%)	4 (5%)
• Other	31 (5%)	1 (1%)	14 (6%)	10 (4%)	6 (7%)
Pathogen					
• Gram positive bacteria	287 (45%)	33 (43%)	115 (48%)	98 (42%)	41 (49%)
• Gram-negative bacteria	320 (50%)	32 (42%)	125 (52%)	114 (48%)	49 (59%)
• Fungus	55 (9%)	6 (8%)	22 (9%)	15 (6%)	12 (14%)
• Virus - SARS-CoV-2	98 (15%) 77 (12%)	9 (12%) 9 (12%)	44 (18%) 37 (15%)	40 (17%) 28 (12%)	5 (6%) 3 (4%)
• Culture-negative	94 (15%)	18 (24%)	28 (12%)	37 (16%)	11 (13%)
Infection characteristics					
• Bacteremia	265 (42%)	35 (46%)	85 (35%)	105 (44%)	40 (48%)
• Candidemia	24 (4%)	4 (5%)	10 (4%)	9 (4%)	1 (1%)
• Secondary sepsis	147 (23%)	16 (21%)	55 (23%)	54 (23%)	22 (27%)
• Multidrug resistant pathogen	134 (21%)	12 (16%)	46 (19%)	52 (22%)	24 (29%)
Organ failure					
• Septic shock	535 (84%)	73 (96%)	192 (79%)	205 (87%)	65 (78%)
• Acute kidney injury*					
- 0	157 (25%)	4 (5%)	98 (41%)	35 (15%)	20 (24%)
- 1	115 (18%)	10 (13%)	45 (19%)	37 (16%)	23 (28%)
- 2	84 (13%)	6 (8%)	37 (15%)	31 (13%)	10 (12%)
- 3 *missing*	280 (44%) *1*	56 (74%)	61 (25%) *1*	133 (56%)	30 (36%)
• ARDS	218 (34%)	34 (45%)	77 (32%)	93 (39%)	14 (17%)
• Thrombocytopenia**	265 (42%)	44 (58%)	68 (28%)	135 (57%)	18 (22%)
• HPD + DIC**	91 (14%)	28 (37%)	9 (4%)	53 (22%)	1 (1%)
• Delirium	205 (32%)	16 (21%)	85 (35%)	73 (31%)	31 (37%)
- Not applicable****	210 (33%)	46 (61%)	67 (28%)	84 (36%)	13 (16%)
*missing*	*2*	*1*	*1*		
• New onset of atrial fibrillation	141 (26%)	12 (20%)	59 (28%)	56 (27%)	14 (24%)
*missing*	*104*	*16*	*32*	*32*	*24*
Outcome					
• In-hospital mortality	*260 (41%)*	*58 (76%)*	*76 (31%)*	*101 (43%)*	*25 (30%)*

*according to KDIGO urine output or creatinine criteria ** platelets < 100.000/µl, *** Hepatobiliary dysfunction (HPD) and disseminated intravascular coagulopathy (DIC) = Bilirubin > 20 µmol/l, INR > 1.5 and platelets < 100.000/µl. **** Testing for delirium not possible due to continuous heavy sedation during the observation period. Thrombocytopenia = platelet count < 100.000/µl. Where variables had missing values, these were listed in the table

*Abbreviations: BMI = body mass index*,* CCI = Charlson Comorbidity Index*,* SOFA = Sequential organ failure assessment*,* SAPS II = Simplified Acute Physiology Score II*,* ARDS = Acute respiratory distress syndrome*,* ECMO = Extracorporeal membrane oxygenation.*

### Latent profile analysis

Based on goodness-of-fit criteria, the optimal model comprised four profiles and was estimated with class-varying unrestricted parameterization (Table S2A). Average latent class probabilities for the most likely class membership by assigned class, as well as entropy, indicated a low risk of classification uncertainty for this model (Table S2A). The median probability of class membership was high in all subgroups, with 1.00 (IQR 0.99–1) in subgroup 1, 0.99 (IQR 0.94–0.99) in subgroup 2, 1.0 (IQR 0.99–1.0) in subgroup 3, and 0.99 (IQR 0.91–1.0) in subgroup 4 (Figure S2A). 76 patients (12%) were assigned to subgroup 1, 242 (38%) to subgroup 2, 236 (37%) to subgroup 3, and 83 (13%) to subgroup 4 (Table 1). The variables included in the model showed different distributions between the identified subgroups (Figures S3A-F).

At the 120–144-hour time point, 84 (13%) of the patients in the initial analysis were dead, 82 (13%) had been transferred to the normal ward, and 25 (4%) were excluded from the repeated latent profile analysis due to missing values in daily routine variables. 446 cases remained in the analysis. The fit statistics of the repeated latent profile analysis indicated three subgroups at the late time point that had comparable characteristics to the original four subgroups (hepatobiliary dysfunction, inflammatory and less severely affected). 91 (20%) patients were assigned to subgroup 1, 203 (46%) to subgroup 2, 152 (34%) to subgroup 3 in the late LPA. The median probabilities of class membership were high (Figure S2B).

### Characterization of the identified subgroups

**Fig. 1:**
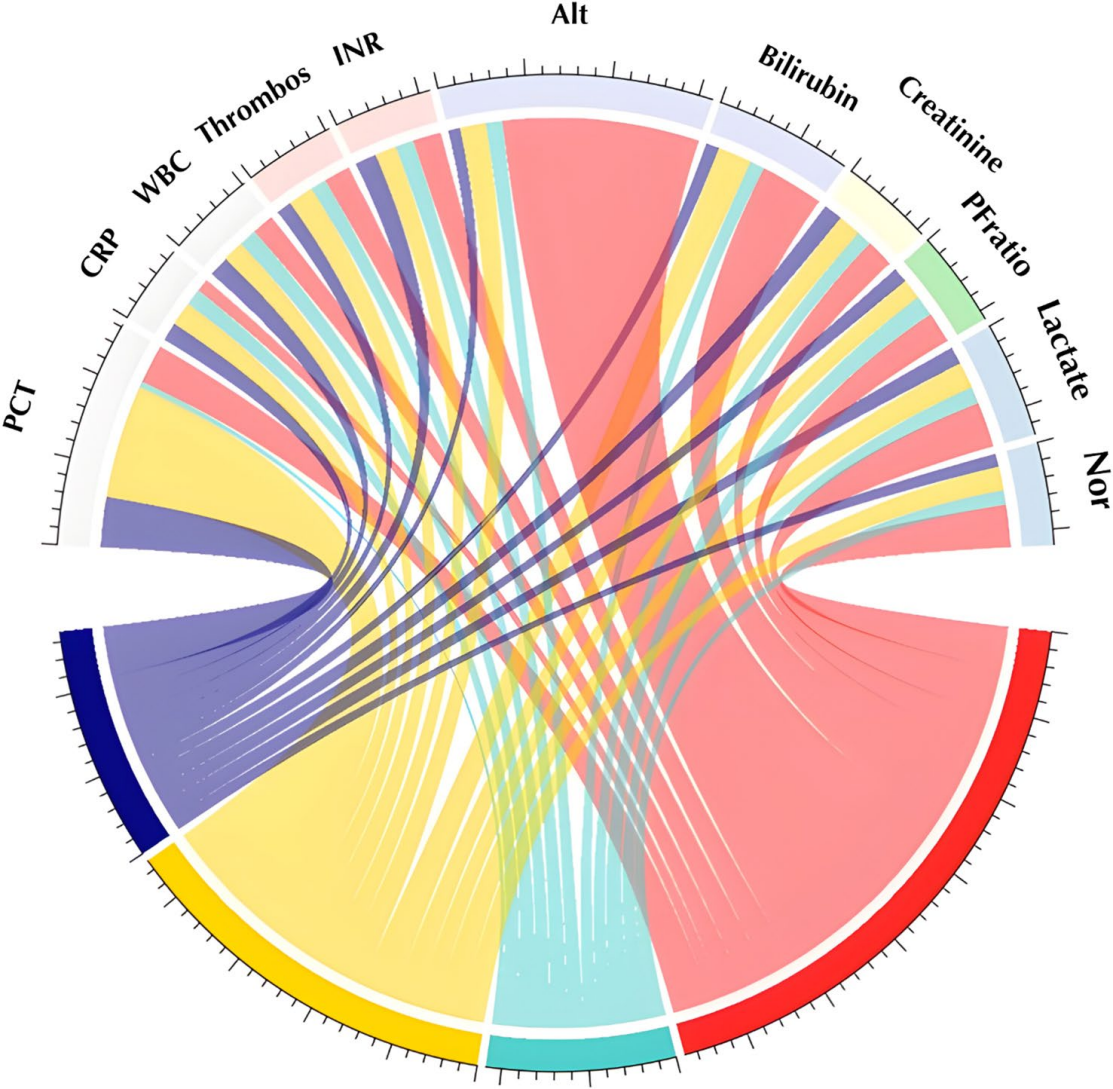
Characteristics of the identified subgroups at the early time point. *Chord plot to show relative differences in class-defining variables between subgroups. The width of the individual traces is determined by the deviation from the median for the individual variable in each subgroup. More pathological values result in wider traces. Abbreviations: PCT = procalcitonin*,* CRP = c-reactive protein*,* WBC = white blood cell count*,* PLT = platelets*,* INR = international normalized ratio of the thrombin time*,* ALT = alanine aminotransferase*,* P/F ratio = ratio of partial pressure of oxygen in the arterial blood to inhaled fraction of oxygen*

The characteristics of the overall patient cohort and identified subgroups are described in Table 1. The subgroups had different baseline characteristics (Fig. 1). Subgroup 1 was defined by elevated ALT, bilirubin, INR, and lactate, and a decreased P/F ratio (Table S8). Patients in subgroup 1 were younger and had fewer comorbidities, but had the highest disease severity. The incidence of septic shock and cardiovascular dysfunction was highest in this group, as indicated by the highest use of inotropes, norepinephrine rate, and serum lactate level (Table S5). They had by far the highest bilirubin and transaminase levels, and the most pronounced adverse effects on hematological parameters such as platelet count and INR. 28 patients (37%) fulfilled the criteria for hepatobiliary dysfunction and disseminated coagulopathy [24]. Subgroup 2 was defined by lower inflammatory markers (WBC, PCT and CRP), creatinine, lactate, ALT and bilirubin levels, as well as low INR (Table S8). Patients in subgroup 2 were most likely to have a pulmonary focus (Table 1). Patients in subgroup 3 were defined by elevated levels of inflammatory markers (WBC, PCT and CRP), creatinine and lactate, low platelets, low ALT levels, and low INR (Table S8). They exhibited a comparable pattern of organ failure to that of subgroup 1, yet were less likely to meet the criteria for HPD + DIC. Subgroup 3 showed the most pronounced elevation in inflammatory marker levels. Subgroup 4 was defined by older age, higher P/F ratio and higher INR, but low levels of markers of hepatobiliary dysfunction and low serum lactate (Table S8). They had more comorbidities (Table 1 and Table S4), and had a higher incidence of infections with multidrug-resistant bacteria. They had the least severe organ failure and required the least invasive organ support (Table S5). In summary, the cohort exhibited distinct clinical manifestations, with SG1 presenting marked hepatobiliary and cardiovascular dysfunction, SG3 displaying hyperinflammation, and SG2 and SG4 demonstrating less severe involvement. Notably, SG4 exhibited a high PCT and INR, distinguishing it from SG2.

### Outcome

**Fig. 2:**
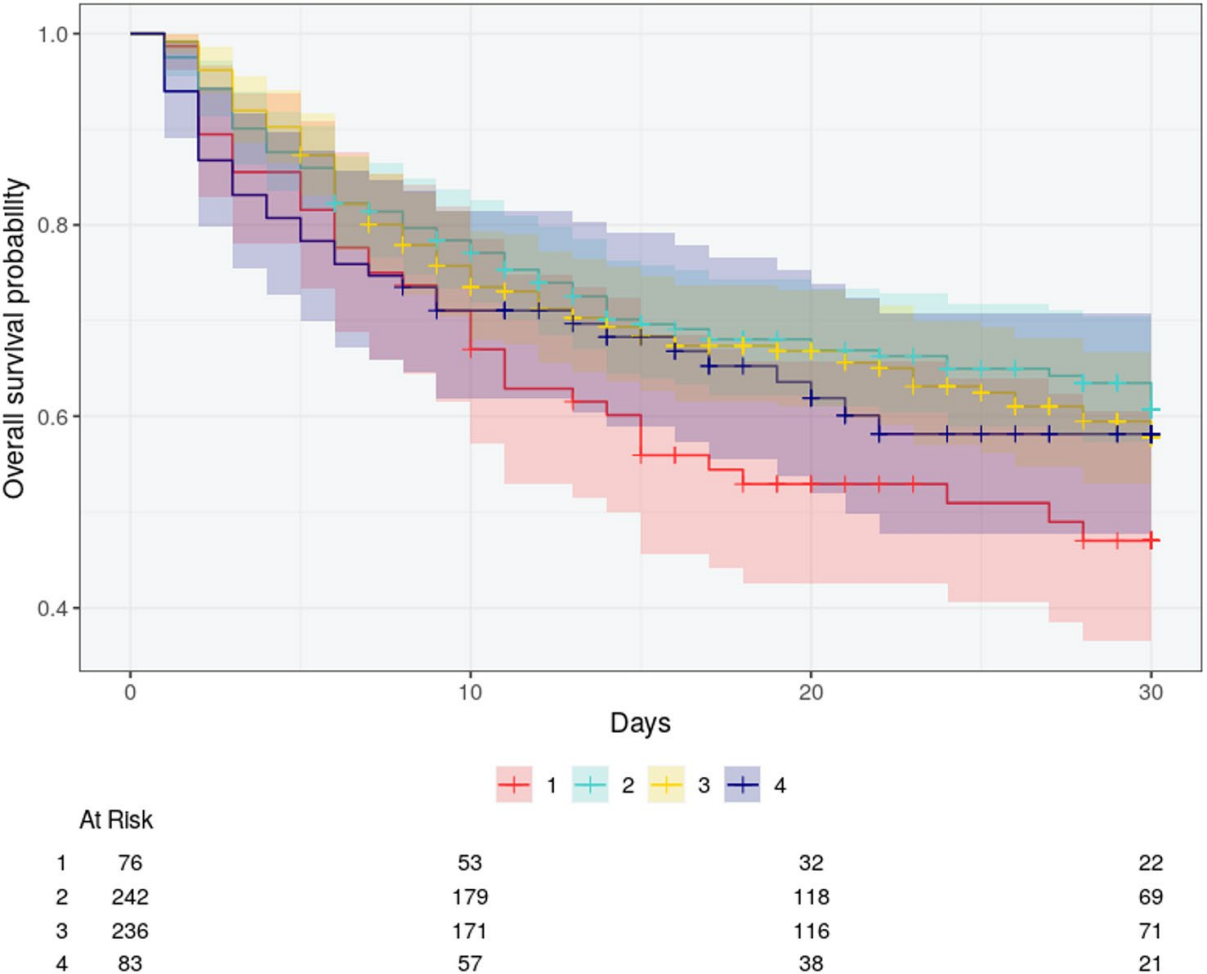
30-day mortality of the identified subgroups. + = censored events

The subgroups demonstrated differences in the outcome (Fig. 2). Subgroups 2 and 4 exhibited the lowest in-hospital mortality, while Subgroup 1 was associated with an in-hospital mortality of 76% (Table 1). Multivariate regression analysis (Table S7) identified subgroups 3 (OR 2.29, 95% CI 1.44, 3.7) and 1 (OR 10.8, 95% CI 5.45, 22.5) as independent risk factors for ICU mortality.

### Time stability

**Fig. 3:**
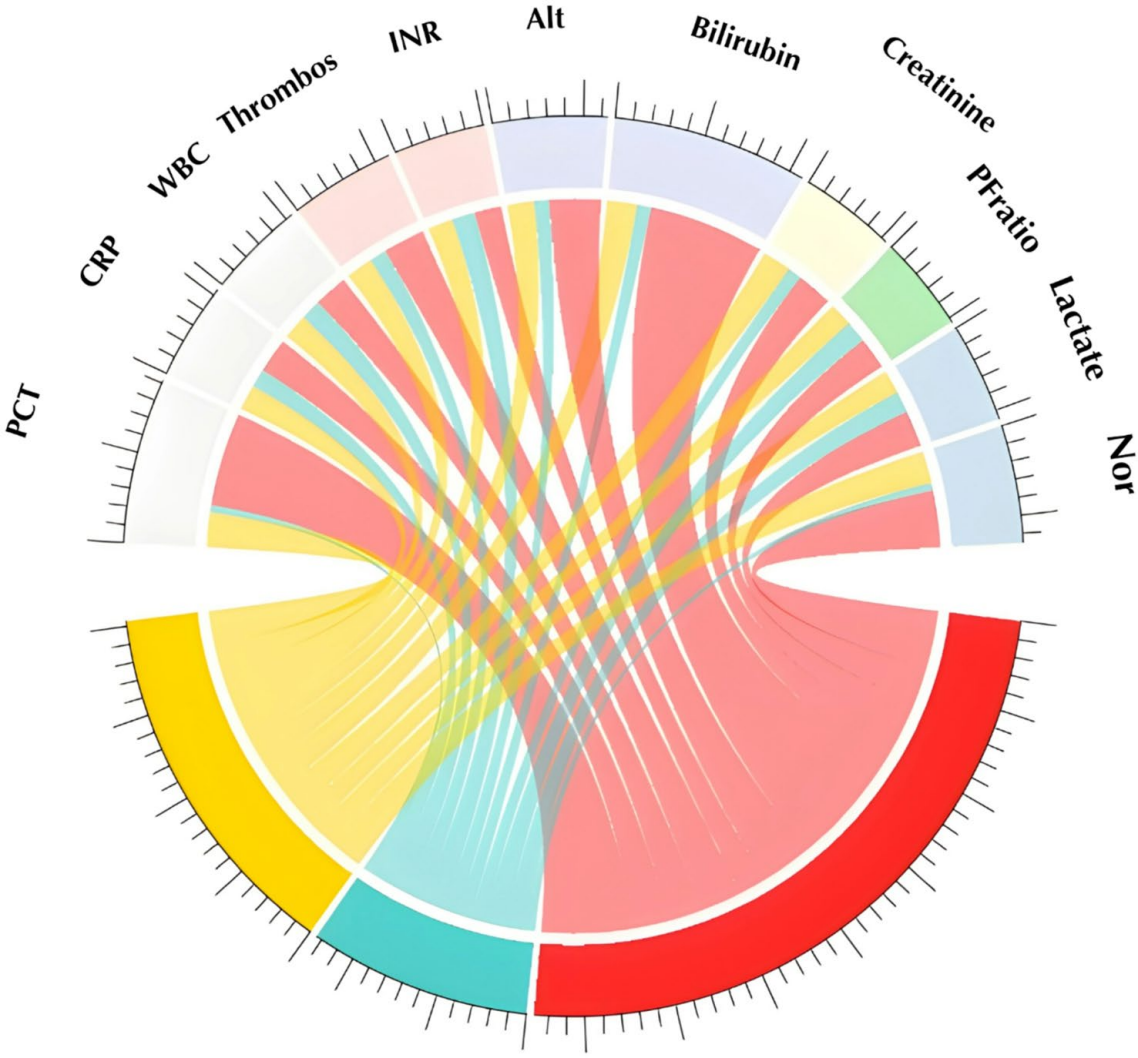
Characteristics of the identified subgroups at the late time point. *Abbreviations: Chord plot to show relative differences in class-defining variables between subgroups. The width of the individual traces is determined by the deviation from the median for the individual variable in each subgroup. More pathological values result in wider traces. PCT = procalcitonin*,* CRP = c-reactive protein*,* WBC = white blood cell count*,* PLT = platelets*,* INR = international normalized ratio of the thrombin time*,* ALT = alanine aminotransferase*,* P/F ratio = ratio of partial pressure of oxygen in the arterial blood to inhaled fraction of oxygen*

**Fig. 4:**
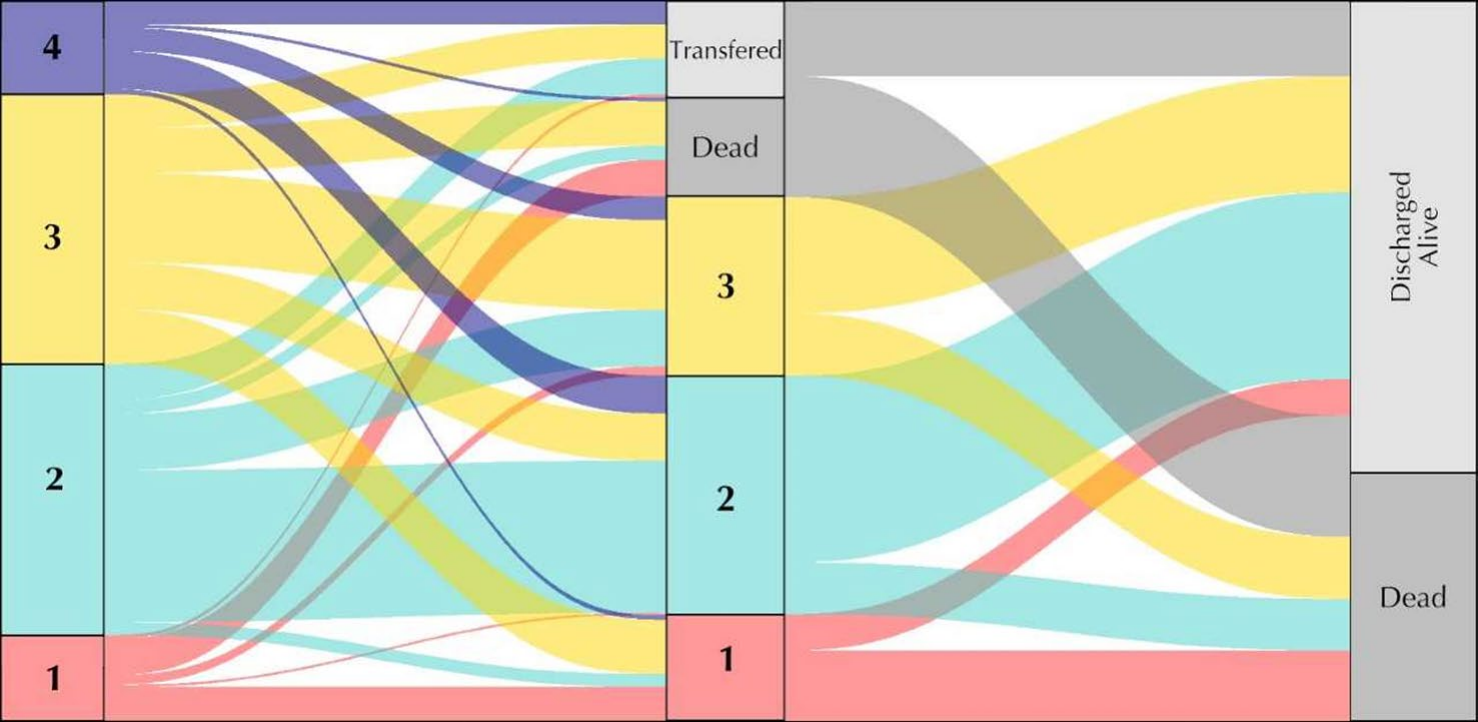
Trajectory of the identified subgroups

**Fig. 5:**
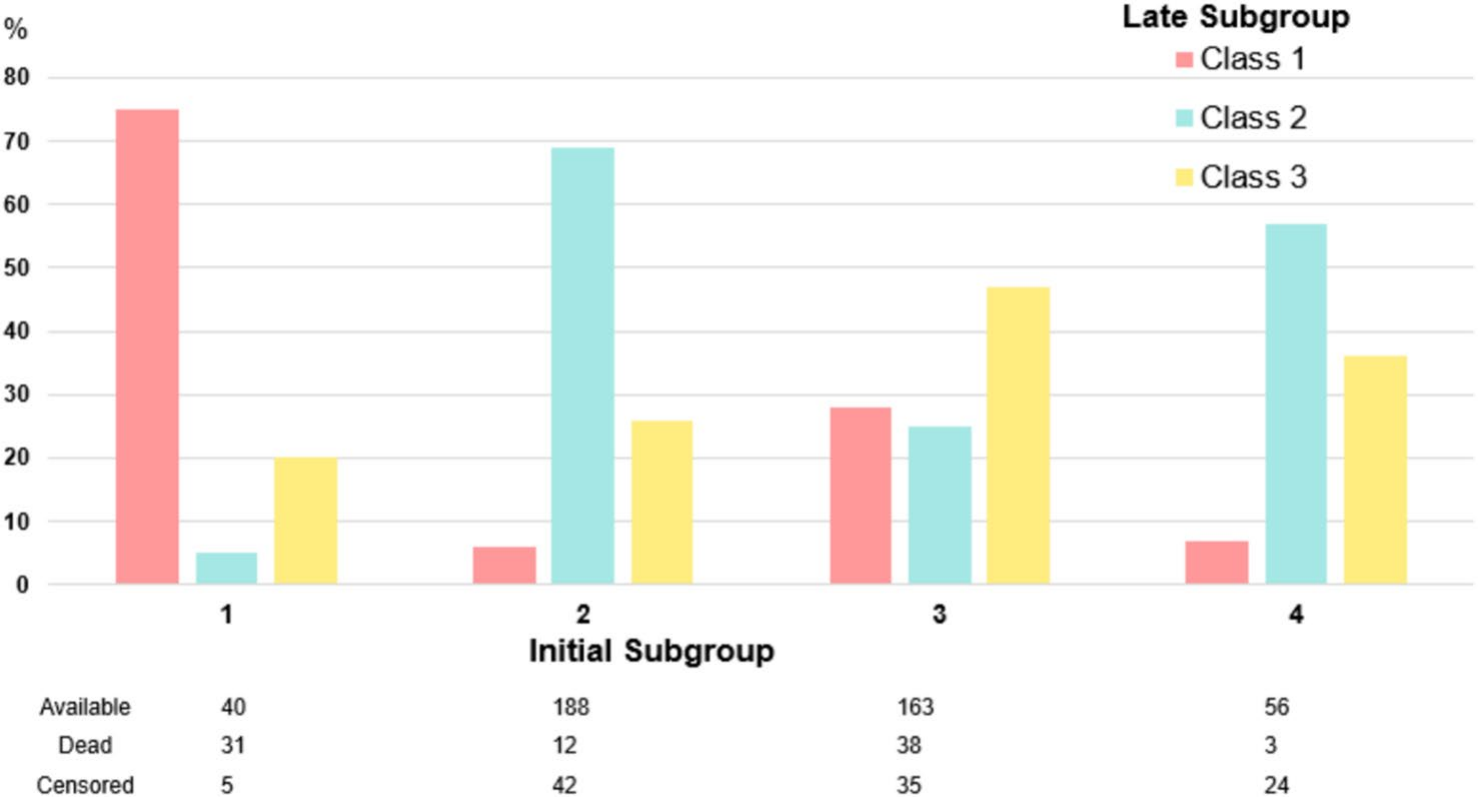
Stability of subgroup assignment over time

In the cohort of patients who remained in the ICU 120–144 h after initiation of sepsis treatment and had not died or been transferred to the regular ward, three subgroups could be distinguished by LPA. The subgroups identified at the late time point showed patterns comparable to those identified during the first 48 h (Fig. 3). SG 1 was characterized by hepatobiliary dysfunction, SG 2 by less severe impairment, and SG 3 by high inflammation and moderate organ failure (Table S8B). There was some mobility between the class assignments from early to late LPA. Figure 4 presents the trajectory of class assignment from the early time point (left column) to the late time point (middle column) and with regard to in-hospital mortality (right column). Subgroup 1 demonstrated good time stability, with the majority of individuals initially assigned to this subgroup either keeping their assigned subgroup or dying. Individuals in other subgroups who changed their assignment to subgroup 1, predominantly did so from subgroup 3 (46/61 (75%)). Inter-subgroup mobility was stronger between subgroups 2–4. Subgroups at the late time point showed a similar pattern regarding mortality as subgroups assigned at the early time point. Figure 5 displays the probability of subgroup assignment depending on the assigned subgroup at the early time point. Individuals who were assigned to subgroup 1 at the early time point had an almost 80% probability of being assigned to subgroup 1 at the late time point. Nearly 30% of individuals assigned to subgroup 3 in the early LPA changed to subgroup 1 in the late LPA. 47–69% of cases in the subgroups 2 to 4 remained in their initially assigned subgroups. The subgroup with the least temporal stability was the hyperinflammatory SG 3. The characteristics of individuals who remained in or left their original subgroup are shown in Tables S6A-D.

Alluvial plot demonstrating the trajectory of subgroup assignment with the repeated measures latent profile analysis. Left column: Subgroup assignment in the early latent profile analysis. Middle column: Subgroup assignment in the late latent profile analysis. Right column: In-hospital mortality. Transferred = individuals were transferred to the normal ward or discharged from the hospital.

This figure illustrates the proportion of individuals assigned to subgroups in the late latent profile analysis. The initial subgroup assignment in the early latent profile analysis is depicted on the x-axis.

## Discussion

Our retrospective study identified four subgroups of sepsis in critically ill patients treated in intensive care from routine clinical data available within the first 48 h of sepsis diagnosis. At 120–144 h after initiation of sepsis treatment, three subgroups persisted among the cohort of patients who had remained in intensive care treatment until that time. The three subgroups of the late model exhibited a similar characteristic to the initial four subgroups (hepatobiliary dysfunction, hyperinflammatory, and less severely affected). Subgroup 1 demonstrated longitudinal stability. There was a significant association between in-hospital mortality and subgroups 1 and 3. The results of the logistic regression modelling demonstrated that the “traditional” subgroups, which were based on the focus of infection, pathogen or pre-existing conditions, showed little association with the new subgroups (Table S9).

The four subgroups identified in our study are highly concordant with the four sepsis phenotypes identified by Seymour and colleagues in the SENECA study with data from patients with sepsis in the emergency department [5]. This finding may be considered unexpected, given that our data were collected in a different treatment setting and healthcare environment. Moreover, our data set encompassed cases of both community-acquired and nosocomial sepsis, as well as a diverse range of pathogens, including viral sepsis caused by the SARS-CoV-2 virus. Subgroup 1 in our dataset is analogous to the δ-phenotype identified in the SENECA cohort [5]. This phenotype is characterized by a high degree of multi-organ failure, a distinctive hepatobiliary dysfunction, and a markedly elevated mortality rate compared to other subgroups. In order to ascertain the stability of these subgroups over time, we conducted an analysis of transition between groups on an individual patient basis during the initial 48 h of critical care treatment and at the 4–5-day mark following the start of critical care treatment. Our longitudinal assessment demonstrated remarkable consistency in the subgroup 1 over time. The subgroups identified in the late model differ from those in the early model in terms of their absolute values. A decrease in inflammation and organ damage markers over the course of 4 days is to be expected clinically and is an expression of the intensive care therapy. Nevertheless, in the late model there are relationally similar subgroups, namely again subgroups characterized by hepatobiliary and cardiovascular dysfunction, persistent hyperinflammation or comparatively low impairment. Interestingly, the late model subgroups, which have significantly lower absolute levels of damage markers, show a comparably poor prognosis as the early model subgroups (Table S10). The transition to subgroup 1 during the course of observation was mitigated primarily by a worsening of hepatobiliary function and was associated with a similar poor prognosis as the initial assignment to SG 1 (Table S10). The pathobiological basis for this phenomenon is currently unclear and warrants further investigation. As our clinical subgroups primarily reflect organ failure and inflammation, it is possible that this is merely a reflection of initial disease severity. However, a multivariate model for the prediction of subgroup assignment for the late model suggests that markers of hepatobiliary dysfunction, procalcitonin and lactate are the main drivers of assignment to SG 1 (Table S8B). Furthermore, a mediation analysis examining the effect of SG1 classification on in-hospital mortality with the SOFA score as a potential mediator showed that the SOFA score did not account for the majority of this effect (Figure S8). This suggests that the constellation of hepatobiliary and cardiovascular dysfunction has a prognostic value beyond that of the SOFA score. The longitudinal analysis of our data suggests that although unsupervised machine learning suggested a model with four subgroups, a model comprising only two to three subgroups may demonstrate superior longitudinal stability. Most patients who switched to subgroup 1 were originally assigned to subgroup 3, which was characterized by the most pronounced elevations of inflammation markers and mild elevations of biomarkers reflecting hepatobiliary dysfunction. There was little mobility from subgroups 2 and 4 towards subgroup 1. While SG1 and 3 as well as SG2 and 4 share some common characteristics, it seems reasonable that other inter-individual differences such as comorbidities and disease severity may mediate the subdivision into smaller subgroups in this real-world ICU cohort. This interpretation would be consistent with the findings of Sihna et al. [6] and DeMerle et al. [7], who identified two distinct phenotypes of sepsis, characterized by hyper- and hypoinflammatory responses, respectively [6]. In both studies, hepatobiliary dysfunction, as evidenced by elevated bilirubin levels, and low platelets were identified as key differentiating factors between those two phenotypes. The combination of hepatobiliary dysfunction and an acquired coagulopathy has lately gained some attention as a marker of a severe prognosis in septic patients [24]. Although subphenotyping approaches incorporating routine clinical data have shown good reproducibility, clinical data are only indirectly mirroring an underlying pathomechanism. To address potential underlying pathomechanisms, the incorporation of biomarkers appears to be the most promising approach [25]. Unfortunately, attempts to combine phenotyping approaches incorporating clinical, biomarker and transcriptomic data have so far shown only low to moderate concordance between different phenotyping approaches [26]. The reduction of complexity that comes with models incorporating only two subgroups may offer a pragmatic way forward. The sepsis subphenotype characterized by hepatobiliary dysfunction and coagulopathy has consistently stood out in various sepsis suphenotyping approaches [5–7, 27]. In view of the high mortality rate observed in this subgroup and the fact that the pathomechanism of sepsis-associated liver injury is still incompletely understood [28], future research should investigate whether this subgroup reflects an underlying pathomechanism or simply the final stage of disease progression.

Our study has several strengths. First, we included a broad spectrum of patients with both nosocomial and community-acquired sepsis and a diverse range of pathogens, thereby reflecting a representative cohort of patients with septic shock in everyday intensive care medicine. Second, we were able to assess longitudinal healthcare data due to the automatic electronic documentation of clinical data in our intensive care unit. Third, the subgroups were identified using a parsimonious set of variables obtained from clinical routine data, which should allow for easy replication of these subgroups. Cohort validation of the identified subgroups was achieved by replicating the subgroups at a different time point, identifying predictors of subgroup assignment (Table S8), and reporting differences across the different subgroups regarding relevant outcomes. Our study has also several limitations. Due to the retrospective design of this study, we were restricted to data collected during the clinical routine. In order to minimize the number of missing values and increase the internal validity of the data due to a lower need of imputation, we extended the timeframe for data collection of class-defining variables to 48 h. Consequently, the identified subgroups may reflect a disease severity that may not yet be evident upon the hospital admission of the individual patient. As other retrospective studies on sepsis phenotyping also used data collected over the first 48 h after admission [6], our data should be comparable. The single-center design of our study may be a limitation in terms of external validity. A key limitation of the present study is the absence of external validation of the full model, which is essential to confirm the generalizability of the findings. Publicly available databases (MIMIC III and IV, eICU) offer insufficient data density in some of the variables that were incorporated into our model. A comparison of our subgroups to an external validation cohort was only possible for a reduced model that lacked key discriminating variables, such as PCT (see Table S11A-F). The absence of accessible, high-resolution external datasets remains a significant challenge. Still, we derived some external validation by identifying subgroups comparable to those of the SENECA cohort [5] in data from a real-world ICU that mirrors the complexities and dynamics of everyday intensive care. To substantiate the external validity of these clinical subphenotypes, their replication is essential in external cohorts. A further limitation regarding external validity is the high number of patients in septic shock in our cohort compared to other sepsis cohorts [29]. This is most likely a center effect, as our cohort represents a European ICU cohort from an academic tertiary referral center. Due to organizational differences in health care systems, European ICU populations have a higher disease severity on average [29]. A number of decisions regarding data selection and preprocessing had to be made for the analysis. We chose to include norepinephrine dose rather than mean arterial pressure in the LPA model, given that the sepsis bundle at our ICU requests a MAP target of 60-65mmHg. As a result, septic shock was reflected by a variance in norepinephrine rate rather than MAP. We furthermore had to make several choices to manage the competing risks of death, discharge and dialysis on biomarkers and endpoints. To allow the application of the same class-defining variables at both time points, PCT was maintained as a variable for the late LPA despite an amount of missing data of 33%. Unfortunately, we lack randomized treatment data to assess heterogeneous treatment effects in the identified subgroups. Latent profile analysis is a probabilistic method in which subgroups are identified based on statistical assumptions. Subgroup identification with such algorithms has been shown to produce more consistent profiles than other unsupervised machine learning algorithms [25]. However, a high degree of freedom regarding modelling choices still results in a variety of identifiable subgroups. Taking this into consideration, appropriate caution should be taken when interpreting these explorative data. To minimize arbitrariness, we carefully adhered to proposed standards for subphenotyping strategies [16] and transparently documented data pre-processing and modelling choices. Lastly, the here identified subgroups represent clinical subphenotypes, not biological endotypes. Correlating these clinical subphenotypes with biological endotypes is a future objective.

## Conclusion

Retrospective latent profile analysis identified four sepsis subgroups from clinical routine data of critically ill septic patients. Subgroup one is characterized by hepatobiliary dysfunction, high mortality and longitudinal stability over time. This subphenotype correlates well with published studies. Given the high mortality of this subgroup and the fact that its specific characteristics have consistently emerged as key differentiators in sepsis phenotyping studies, future research should investigate the underlying pathomechanism of this phenomenon.

## Electronic supplementary material

Below is the link to the electronic supplementary material.

Supplementary Material 1

Supplementary Material 2

## Data Availability

No datasets were generated or analysed during the current study.

## Abbreviations

ALT: Alanine aminotransferase
ARDS: Acute Respiratory Distress Syndrome
BMI: Body mass index
CCI: Charlson Comorbidity Index
CRP: C-reactive protein
DIC: Disseminated intravascular coagulopathy
ECMO: Extracorporeal membrane oxygenation
ICU: Intensive care unit
IQR: Interquartile range
INR: International normalized ratio of prothrombin time
HPD: Hepatobiliary dysfunction
LPA: Latent profile analysis
PCT: Procalcitonin
P/F ratio: Ratio of PaO2 to the fractional inspired oxygen
SAPS II: Simplified Acute Physiology Score II
SARS-CoV-2: Severe acute respiratory syndrome corona virus type 2
SOFA: Sequential organ failure assessment
WBC: White blood cell count
