# Supplementary figures and images for: Identification and longitudinal assessment of sepsis phenotypes derived from routine clinical data in critically ill patients: a retrospective repeated measures latent profile analysis
Source: Infection. 2025 Jul 23;53(6):2633–44. doi: 10.1007/s15010-025-02607-8 (PMC12675699; doi:10.1007/s15010-025-02607-8)

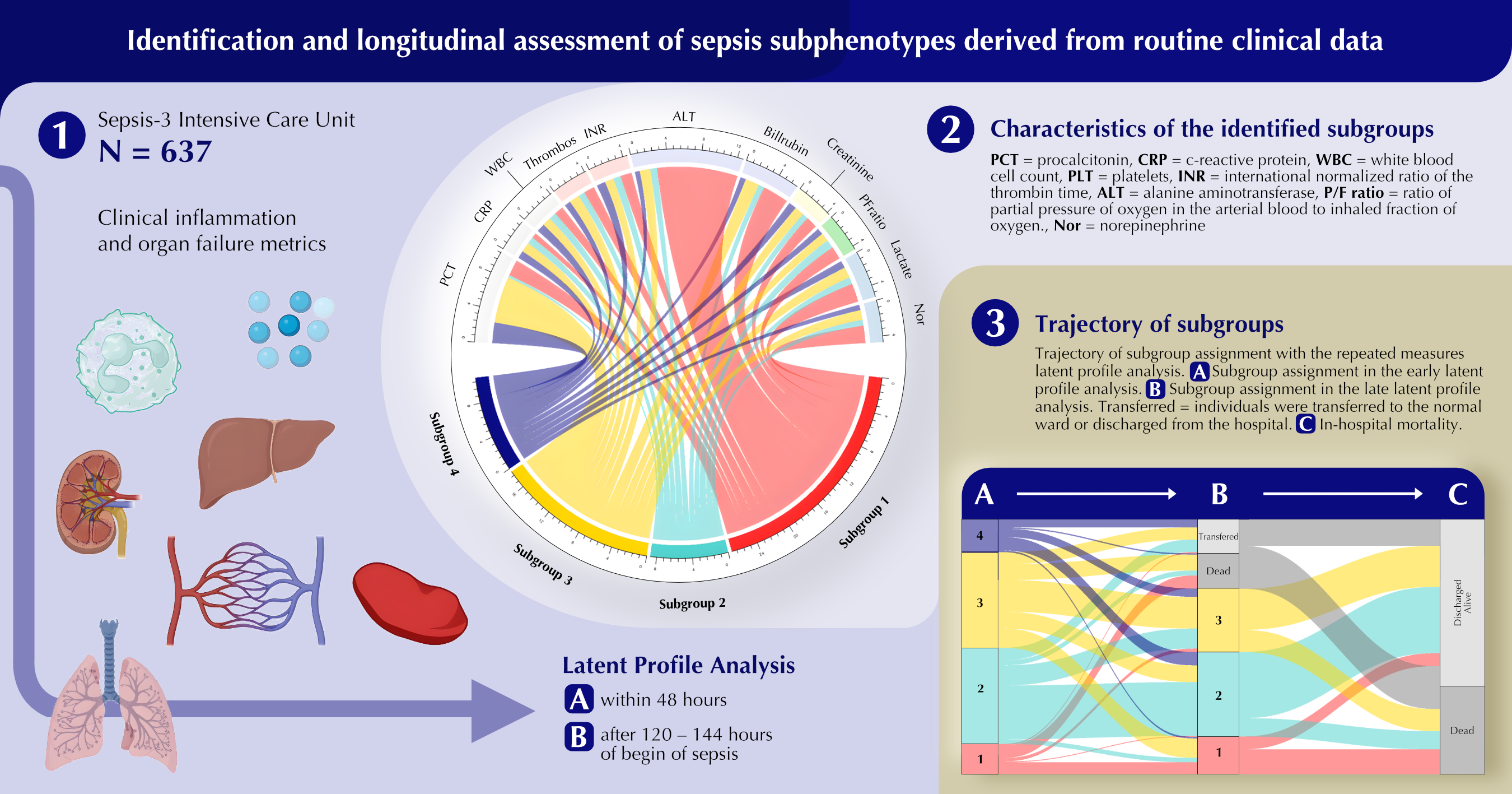

Supplement: Supplementary file 1 — Supplementary Material 1 [file 15010_2025_2607_MOESM1_ESM.png]
