# Supplementary material for: Identification and longitudinal assessment of sepsis phenotypes derived from routine clinical data in critically ill patients: a retrospective repeated measures latent profile analysis
Source: Infection. 2025 Jul 23;53(6):2633–44. doi: 10.1007/s15010-025-02607-8 (PMC12675699; doi:10.1007/s15010-025-02607-8)
Supplement: Supplementary file 2 — Supplementary Material 2 [file 15010_2025_2607_MOESM2_ESM.docx]

**Table of Contents page**

1. **Detailed description of data collection and preprocessing 2-3**
2. **Figure S1 – Consort diagram 4**
3. **Missing data management 5-6**

- Tables S1A-B: Missing data in variables used for LPA
- Figure S1A: Distribution of variables before and after imputation

1. **Table S2A-G: Model fit values of latent profile analyses 7-10**
2. **Figures 2A-B: Probability of subgroup assignments 12**
3. **Figures S3-F: Density plots of class-defining variables in the LPA 13-18**
4. **Table S3: Variables not included in the LPA 19**
5. **Figure S4: Correlation matrix 21**
6. **Figure S5: Distribution of variables reflecting disease severity 22**
7. **Figures S6A-B: Distribution of class-defining variables in LPA 23**
8. **Table S4: History of pre-existing conditions 24-25**
9. **Table S5: Baseline characteristics 26-27**
10. **Tables S6A-D: Longitudinal characteristics of SG 1-4 28-31**
11. **Table S7. Multivariate regression analysis: Independent risk factors for
    in-hospital mortality 32**
12. **Table S8A-B. Multivariate regression analysis: Predictors of subgroup assignment for the early and late model 33-34**
13. **Table S9. Multivariate regression analysis: Risk factors for subgroup assignment based on patient and infection characteristics 35**
14. **Table S10. Outcome of the individual subgroups 35**
15. **Figure S7: Original alluvial plots 37**
16. **Figure S8: Mediation analysis 38**
17. **Tables S11A-F: Validation of a reduced model in the eICU database 39-43**

*Data collection*

An investigator screened all electronic files of patients with the documented diagnosis of “sepsis” or “septic shock”. The first time point when patients met the sepsis-3 criteria was recorded as the time point of study inclusion. We extracted a comprehensive trajectory of clinical and biomarker values over a course of seven days. All patients were followed up until death or hospital discharge, whichever came first. Clinical data of all these patients were extracted manually into a Microsoft Excel (Microsoft Corporation, Redmond, USA) study worksheet. The data was stored on a secure hospital server. After completion of the database, an independent physician reviewed all cases. In case of discrepancies between those two reviewers, a third reviewer investigated those cases. In-house mortality data, as well as data concerning the length of hospital stay were obtained from the Enterprise Clinical Research Data Warehouse (ECRDW) at Hannover Medical School (Hannover, Germany). After data validation and merging of clinical and outcome data, the data base was anonymized for further investigations.

*Data preprocessing*

For the phenotyping, we obtained clinical and biomarker values at two different time points: a) within 48 hours of inclusion (early), and 120 – 144 hours after inclusion (late). The intervals for data collection were extended to 48 hours to minimize the amount of missing data. In these early and late 48 hour time periods, the worst value for each variable was collected. This corresponded to the highest value in most variables, except for the ratio of partial pressure of arterial oxygen to the fractional inspired oxygen (P/F ratio) and platelets, for which the lowest available value was obtained. For all patients receiving invasive or noninvasive ventilation, a P/F ratio was available. If no respiratory support (oxygen supplementation, noninvasive, or invasive ventilation) was required, a P/F ratio of 300 was encoded. In cases where a minimum of 4 liters of oxygen was administered, a P/F ratio of 200 was assigned. If VV-ECMO was required, a P/F ratio of 80 was encoded. We took the highest documented noradrenaline rate (in µg/kg/min) in the electronic health database during the respective time frame. We assigned a value of zero if no noradrenaline was administered at the respective time frame. In accordance with the established protocol of the ICU, the administration of norepinephrine was regulated to assure a mean arterial pressure within the target range of 60 – 65 mmHg. Hyperbilirubinemia was defined as a serum bilirubin level > 20 µmol/l. Hepatobiliary dysfunction and disseminated intravascular coagulopathy (HPD + DIC) was defined as in previous studies (5) as the combination of bilirubin > 20 µmol/l, INR > 1,5, and a platelet count of < 100.000/ µl. Thrombocytopenia was defined as a platelet count < 100.000/ µl. Acute kidney injury was defined according to KDIGO urine output or creatinine criteria (6). The Charlson Comorbiditiy Index (CCI) is a widely utilized scoring system for comorbidities. CCI scores had to be calculated retrospectively based on chart review as they were not available in electronic health database. The Sequential Organ Failure Assessment (SOFA) and the Simplified Acute Physiology Score II (SAPSII) are severity of illness risk scores that were assessed during daily routine. Information on septic foci, pathogens, infection and organ failure characteristics was obtained by chart review. The focus category “other” includes cases with meningoencephalitis, otorhinolaryngological or maxillofacial foci. Secondary sepsis was defined as nosocomial sepsis acquired more than 48 hours after admission to the ICU. “Culture negative” includes all cases, where no pathogen was found.

**Figure S1. Consort diagram.**

**
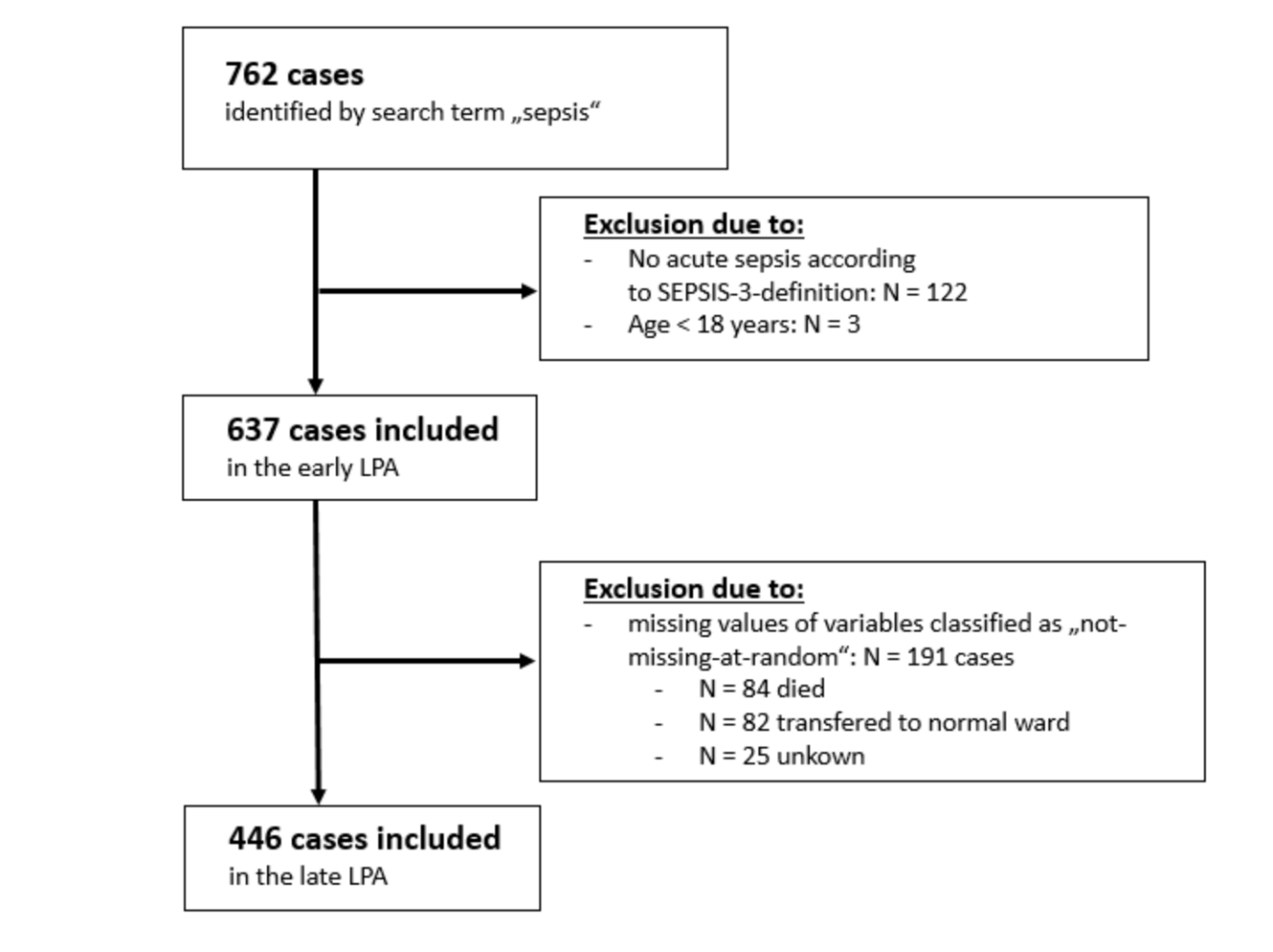
**

LPA = latent profile analysis. The „early“ LPA comprises data of the first 48 hours within study inclusion. The “late” LPA comprises data of the time point of 120 – 144 hours after study inclusion. Missing values of variables available during daily clinical routine in the intensive care unit, were considered to be not missing at random.

**Table S1A.** Assessment of missing data for variables used in the latent profile analysis – early model (N = 637)

|  |  |  | Missingness in subgroups |  |  |
| --- | --- | --- | --- | --- | --- |
| Variable | Overall  missingness | 1  N = 76 | 2  N = 242 | 3  N = 236 | 4  N = 83 |
| Age | 0 | - | - | - | - |
| CRP | 0 | - | - | - | - |
| PCT | 63 | 11 | 20 | 18 | 14 |
| White blood cell count | 0 | - | - | - | - |
| Platelets | 0 | - | - | - | - |
| ALT | 27 | 1 | 5 | 18 | 3 |
| INR | 0 | - | - | - | - |
| Bilirubin | 1 | - | 1 | - | - |
| Creatinine | 0 | - | - | - | - |
| Norepinephrine rate | 0 | - | - | - | - |
| P/F ratio | 0 | - | - | - | - |
| Lactate | 0 | - | - | - | - |

*Abbreviations: CRP = c-reactive protein, PCT = procalcitonin, WBC = white blood cell count, INR = international normalized ratio of prothrombin time, ALT = alanine aminotransferase, P/F ratio = ratio of partial pressure of arterial oxygen to fraction of inhaled oxygen.*

**Figure S1A: Distribution of variables before and after imputation**


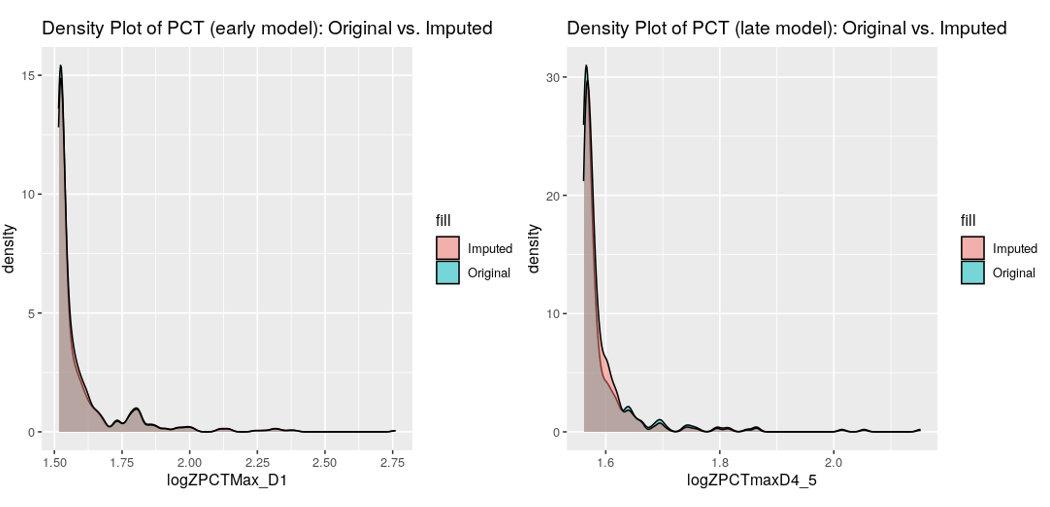


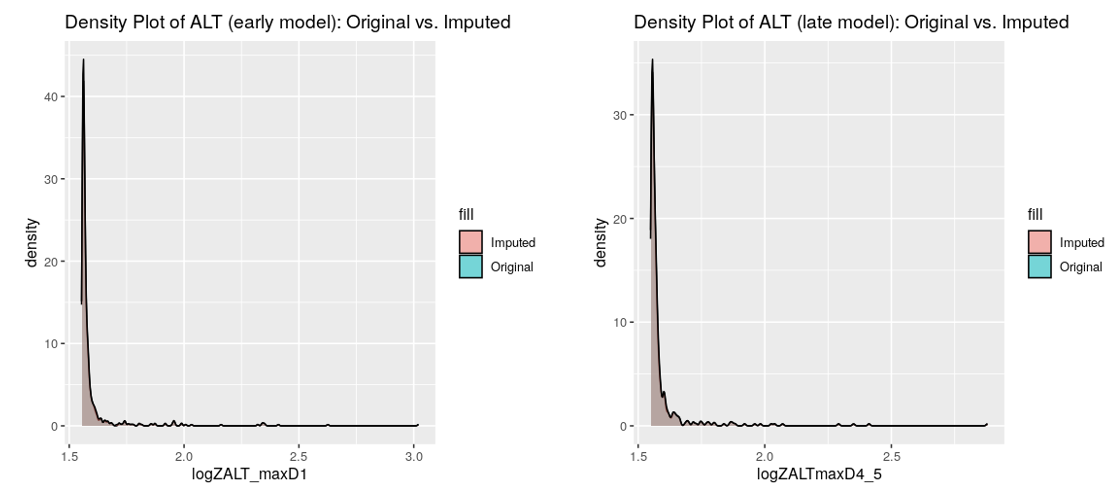


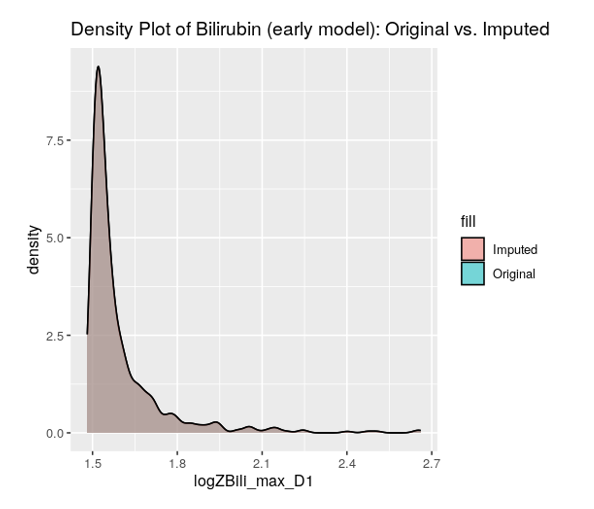


*Abbreviations: PCT = procalcitonin, ALT = alanine aminotransferase, Bili = bilirubin*

**Table S1B.** Assessment of missing data for variables used in the latent profile analysis - late model

|  |  |  |  | Imputation in subgroups |  |
| --- | --- | --- | --- | --- | --- |
| Variable | Overall  Missingness* (N = 637) | Overall imputed missing values in late LPA  (N = 446) | 1  N = 91 | 2  N = 203 | 3  N = 152 |
| Age | 0 |  | - | - | - |
| CRP | 127 |  | - | - | - |
| PCT | 307 | 148 | 18 | 86 | 44 |
| White blood cell count | 131 |  | - | - | - |
| Platelets | 126 |  | - | - | - |
| ALT | 171 | 21 | 2 | 13 | 6 |
| INR | 147 |  | - | - | - |
| Bilirubin | 156 |  | - |  | - |
| Creatinine | 134 |  | - | - | - |
| Norepinephrine rate | 159 |  | - | - | - |
| P/F ratio | 159 |  | - | - | - |
| Lactate | 160 |  | - | - | - |
|  |  |  |  |  |  |

* Missing values at 120 – 144 hours after study inclusion in the overall cohort, before exclusion of cases with missing values not-at-random (MNAR). **Missing values of cases left in the late latent profile analysis after exclusion of MNAR. *Abbreviations: CRP = c-reactive protein, PCT = procalcitonin, WBC = white blood cell count, INR = international normalized ratio of prothrombin time, ALT = alanine aminotransferase, P/F ratio = ratio of partial pressure of arterial oxygen to fraction of inhaled oxygen.*

**Table S2A.** Model fit values of the latent profile analysis – Early model

|  | LL | AIC | BIC | Entropy | | Prob (min) | Prob (max) | N (Min) | N (Max) | BLRT(p) |
| --- | --- | --- | --- | --- | --- | --- | --- | --- | --- | --- |
| EEI - 1 Profile | 3125 | -6203 | -6096 | | 1 | 1 | 1 | 1 | 1 |  |
| EEI - 2 Profiles | 3410 | -6746 | -6581 | | 0.93 | 0.93 | 0.99 | 0.17 | 0.83 | .01 |
| EEI - 3 Profiles | 3600 | -7099 | -6876 | | 0.95 | 0.92 | 0.99 | 0.05 | 0.80 | .01 |
| EEI- 4 Profiles | 3643 | -7161 | -6880 | | 0.85 | 0.70 | 0.96 | 0.05 | 0.68 | .01 |
| EEI – 5 Profiles | 3852 | -7552 | -7213 | | 0.87 | 0.72 | 0.96 | 0.05 | 0.65 | *NA* |
| EEI – 6 Profiles | 4056 | -7935 | -7538 | | 0.90 | 0.73 | 1.0 | 0.02 | 0.61 | *NA* |
| VVV- 1 Profile | 3450 | -6719 | -6318 | | 1 | 1 | 1 | 1 | 1 |  |
| VVV- 2 Profiles | 5874 | -11387 | -10580 | | 0.96 | 0.98 | 0.99 | 0.37 | 0.63 | .01 |
| VVV- 3 Profiles | 6565 | -12586 | -11374 | | 0.94 | 0.97 | 0.98 | 0.12 | 0.46 | .01 |
| VVV- 4 Profiles | 6875 | -13024 | -11406 | | 0.93 | 0.89 | 0.98 | 0.12 | 0.38 | .01 |
| VVV- 5 Profiles | *NA* | *NA* | *NA* | | *NA* | *NA* | *NA* | *NA* | *NA* | *NA* |
| VVV- 6 Profiles | *NA* | *NA* | *NA* | | *NA* | *NA* | *NA* | *NA* | *NA* | *NA* |

*Abbreviations: EEI = Equal variances and covariances fixed to 0, VVV = varying variances and varying covariances, LL = Log Likelihood, AIC = Akaike Information criterion, BIC = Bayesian information criterion, N (min) = proportion of the sample assigned to the smallest profile, N(max) = proportion of the sample assigned to the largest profile. BLRT(p) = p-value for the bootstrapped likelihood ratio test, SALPP = smallest average latent profile posterior probability, Prob(Min): Minimal average latent class probability for most likely class membership. Prob(Max) Maximal average latent class probability for most likely class membership. With VVV, no more than four profiles converged.*

**Table S2B.** Model fit values of the latent profile analysis – Late model

|  | LL | AIC | BIC | Entropy | | Prob (min) | Prob (max) | N (Min) | N (Max) | BLRT(p) |
| --- | --- | --- | --- | --- | --- | --- | --- | --- | --- | --- |
| VVV- 1 Profile | 2916 | -5651 | -5282 | | 1 | 1 | 1 | 1 | 1 |  |
| VVV- 2 Profiles | 4422 | -8482 | -7740 | | 0.97 | 0.99 | 0.99 | 0.39 | 0.61 | *NA* |
| VVV- 3 Profiles | 4762 | -8979 | -7864 | | 0.94 | 0.97 | 0.99 | 0.24 | 0.39 | *NA* |
| VVV- 4 Profiles | 4956 | -9186 | -7698 | | 0.94 | 0.97 | 0.98 | 0.20 | 0.30 | *NA* |
| VVV- 5 Profiles | 5147 | -9385 | -7524 | | 0.96 | 0.97 | 0.99 | 0.12 | 0.24 | *NA* |
| VVV- 6 Profiles | 5198 | -9306 | -7072 | | 0.97 | 0.97 | 1.0 | 0.05 | 0.24 | *NA* |

*Abbreviations: VVV = varying variances and varying covariances, LL = Log Likelihood, AIC = Akaike Information criterion, BIC = Bayesian information criterion, N (min) = proportion of the sample assigned to the smallest profile, N(max) = proportion of the sample assigned to the largest profile. BLRT(p) = p-value for the bootstrapped likelihood ratio test, SALPP = smallest average latent profile posterior probability, Prob(Min): Minimal average latent class probability for most likely class membership. Prob(Max) Maximal average latent class probability for most likely class membership*

**Table S2C.** Model fit values of the sensitivity analyses: early LPA without ALT

|  | LL | AIC | BIC | Entropy | | Prob (min) | Prob (max) | N (Min) | N (Max) | BLRT(p) |
| --- | --- | --- | --- | --- | --- | --- | --- | --- | --- | --- |
| VVV- 1 Profile | 2959 | -5764 | -5421 | | 1 | 1 | 1 | 1 | 1 |  |
| VVV- 2 Profiles | 4701 | -9092 | -8401 | | 0.90 | 0.98 | 0.99 | 0.37 | 0.63 | .01 |
| VVV- 3 Profiles | 5185 | -9905 | -8866 | | 0.88 | 0.92 | 0.98 | 0.08 | 0.50 | .01 |
| VVV- 4 Profiles | 5524 | -10426 | -9040 | | 0.88 | 0.92 | 0.97 | 0.09 | 0.34 | .01 |
| VVV- 5 Profiles | 5679 | -10580 | -8847 | | 0.87 | 0.88 | 0.97 | 0.07 | 0.29 | .01 |
| VVV- 6 Profiles | 5794 | -10653 | -8572 | | 0.87 | 0.92 | 0.96 | 0.06 | 0.23 | .01 |

Latent profile analysis with the set of variables included in the early model, but without ALT. Missing values were imputed. *Abbreviations: VVV = varying variances and varying covariances, LL = Log Likelihood, AIC = Akaike Information criterion, BIC = Bayesian information criterion, N (min) = proportion of the sample assigned to the smallest profile, N(max) = proportion of the sample assigned to the largest profile. BLRT(p) = p-value for the bootstrapped likelihood ratio test, SALPP = smallest average latent profile posterior probability, Prob(Min): Minimal average latent class probability for most likely class membership. Prob(Max) Maximal average latent class probability for most likely class membership*

**Table S2D.** Model fit values of the sensitivity analyses: early LPA without PCT

|  | LL | AIC | BIC | Entropy | | Prob (min) | Prob (max) | N (Min) | N (Max) | BLRT(p) |
| --- | --- | --- | --- | --- | --- | --- | --- | --- | --- | --- |
| VVV- 1 Profile | 3055 | -5956 | -5613 | | 1 | 1 | 1 | 1 | 1 |  |
| VVV- 2 Profiles | 5329 | -10349 | -9658 | | 0.95 | 0.98 | 0.99 | 0.25 | 0.75 | .01 |
| VVV- 3 Profiles | 5825 | -11183 | -10145 | | 0.91 | 0.94 | 0.98 | 0.12 | 0.51 | .01 |
| VVV- 4 Profiles | 6073 | -11525 | -10139 | | 0.89 | 0.91 | 0.97 | 0.1 | 0.33 | .01 |
| VVV- 5 Profiles | 6301 | -11823 | -10090 | | 0.88 | 0.89 | 0.96 | 0.08 | 0.32 | .01 |
| VVV- 6 Profiles | 6445 | -11955 | -9874 | | 0.89 | 0.88 | 0.98 | 0.06 | 0.30 | .01 |

Latent profile analysis with the set of variables included in the early model, but without PCT. Missing values were imputed. *Abbreviations: VVV = varying variances and varying covariances, LL = Log Likelihood, AIC = Akaike Information criterion, BIC = Bayesian information criterion, N (min) = proportion of the sample assigned to the smallest profile, N(max) = proportion of the sample assigned to the largest profile. BLRT(p) = p-value for the bootstrapped likelihood ratio test, SALPP = smallest average latent profile posterior probability, Prob(Min): Minimal average latent class probability for most likely class membership. Prob(Max) Maximal average latent class probability for most likely class membership*

**Table S2E.** Model fit values of the sensitivity analyses: Early LPA without norepinephrine rate

|  | LL | AIC | BIC | Entropy | | Prob (min) | Prob (max) | N (Min) | N (Max) | BLRT(p) |
| --- | --- | --- | --- | --- | --- | --- | --- | --- | --- | --- |
| VVV- 1 Profile | 2717 | -5279 | -4936 | | 1 | 1 | 1 | 1 | 1 |  |
| VVV- 2 Profiles | 4524 | -8739 | -8048 | | 0.96 | 0.98 | 1 | 0.40 | 0.60 | .01 |
| VVV- 3 Profiles | 4875 | -9285 | -8246 | | 0.91 | 0.95 | 0.97 | 0.33 | 0.35 | .01 |
| VVV- 4 Profiles | 5242 | -9863 | -8477 | | 0.91 | 0.92 | 0.97 | 0.17 | 0.34 | .01 |
| VVV- 5 Profiles | 5502 | -10226 | -8492 | | 0.91 | 0.93 | 0.96 | 0.14 | 0.24 | .01 |
| VVV- 6 Profiles | 5629 | -10324 | -8243 | | 0.92 | 0.93 | 0.97 | 0.1 | 0.22 | .01 |

Latent profile analysis without missing values and without the following variables: Norepinephrine rate. *Abbreviations: VVV = varying variances and varying covariances, LL = Log Likelihood, AIC = Akaike Information criterion, BIC = Bayesian information criterion, N (min) = proportion of the sample assigned to the smallest profile, N(max) = proportion of the sample assigned to the largest profile. BLRT(p) = p-value for the bootstrapped likelihood ratio test, SALPP = smallest average latent profile posterior probability, Prob(Min): Minimal average latent class probability for most likely class membership. Prob(Max) Maximal average latent class probability for most likely class membership*

**Table S2F.** Model fit values of the sensitivity analyses: Early LPA without norepinephrine rate and ALT

|  | LL | AIC | BIC | Entropy | | Prob (min) | Prob (max) | N (Min) | N (Max) | BLRT(p) |
| --- | --- | --- | --- | --- | --- | --- | --- | --- | --- | --- |
| VVV- 1 Profile | 2226 | -4323 | -4033 | | 1 | 1 | 1 | 1 | 1 |  |
| VVV- 2 Profiles | 3342 | -6423 | -5839 | | 0.86 | 0.91 | 0.98 | 0.44 | 0.56 | .01 |
| VVV- 3 Profiles | 3628 | -6863 | -5985 | | 0.80 | 0.91 | 0.98 | 0.25 | 0.40 | .01 |
| VVV- 4 Profiles | 3822 | -7118 | -5945 | | 0.82 | 0.88 | 0.94 | 0.20 | 0.27 | .01 |
| VVV- 5 Profiles | 4047 | -7437 | -5971 | | 0.87 | 0.88 | 0.97 | 0.15 | 0.31 | .01 |
| VVV- 6 Profiles | 4086 | -7383 | -5623 | | 0.88 | 0.88 | 0.98 | 0.11 | 0.31 | 1 |

Latent profile analysis without missing values and without the following variables: Norepinephrine rate and alanine aminotransferase. *Abbreviations: VVV = varying variances and varying covariances, LL = Log Likelihood, AIC = Akaike Information criterion, BIC = Bayesian information criterion, N (min) = proportion of the sample assigned to the smallest profile, N(max) = proportion of the sample assigned to the largest profile. BLRT(p) = p-value for the bootstrapped likelihood ratio test, SALPP = smallest average latent profile posterior probability, Prob(Min): Minimal average latent class probability for most likely class membership. Prob(Max) Maximal average latent class probability for most likely class membership*

**Table S2G.** Model fit values of the sensitivity analyses: early LPA without norepinephrine rate, ALT and PCT, no missings

|  | LL | AIC | BIC | Entropy | | Prob (min) | Prob (max) | N (Min) | N (Max) | BLRT(p) |
| --- | --- | --- | --- | --- | --- | --- | --- | --- | --- | --- |
| VVV- 1 Profile | 1829 | -3549 | -3309 | | 1 | 1 | 1 | 1 | 1 |  |
| VVV- 2 Profiles | 2578 | -4938 | -4452 | | 0.86 | 0.94 | 0.98 | 0.37 | 0.63 | .01 |
| VVV- 3 Profiles | 2772 | -5215 | -4485 | | 0.80 | 0.84 | 0.93 | 0.28 | 0.43 | .01 |
| VVV- 4 Profiles | 2882 | -5327 | -4351 | | 0.82 | 0.84 | 0.94 | 0.20 | 0.33 | .01 |
| VVV- 5 Profiles | 3022 | -5496 | -4275 | | 0.85 | 0.86 | 0.98 | 0.08 | 0.37 | .01 |
| VVV- 6 Profiles | 3115 | -5573 | -4107 | | 0.85 | 0.87 | 0.94 | 0.1 | 0.28 | .01 |

Latent profile analysis without missing values and without the following variables: Norepinephrine rate, procalcitonin and alanine aminotransferase. *Abbreviations: LL = Log Likelihood, AIC = Akaike Information criterion, BIC = Bayesian information criterion, N (min) = proportion of the sample assigned to the smallest profile, N(max) = proportion of the sample assigned to the largest profile. BLRT(p) = p-value for the bootstrapped likelihood ratio test, SALPP = smallest average latent profile posterior probability, Prob(Min): Minimal average latent class probability for most likely class membership. Prob(Max) Maximal average latent class probability for most likely class membership*

**Figure S2A.** Probability of subgroup assignment in the early model.


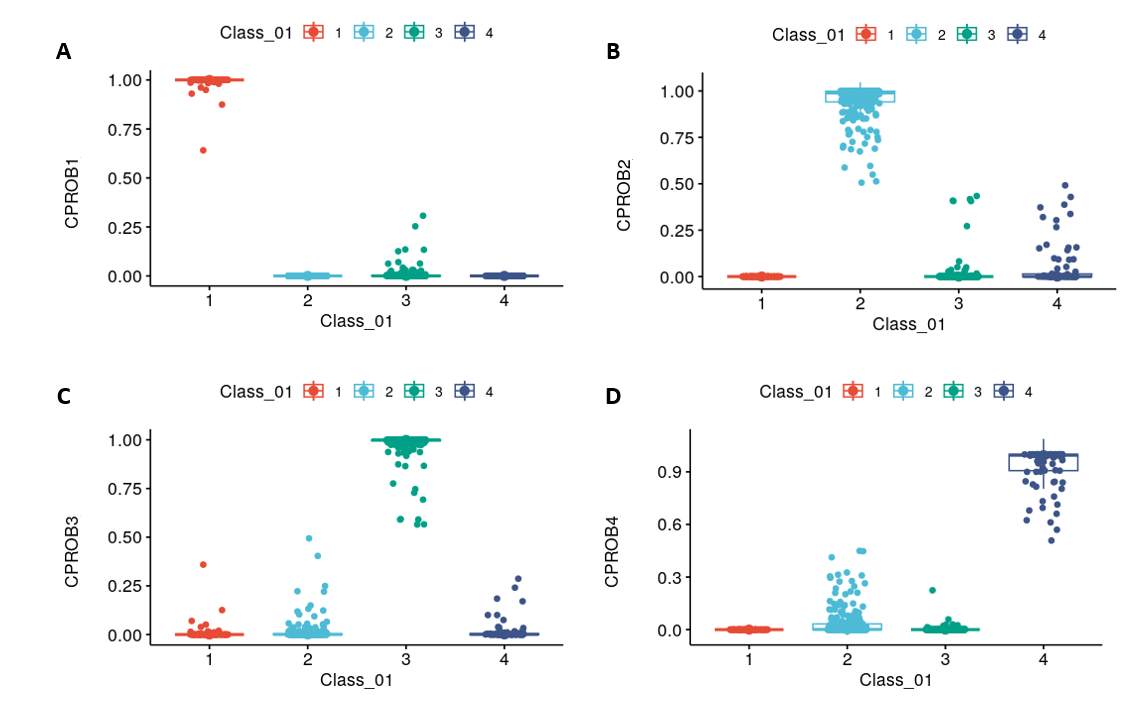


Probabilities of assignment to subgroup for each subgroup. A = Probability of assignment to Subgroup 1, B = probability of assignment to subgroup 2, C = probability of assignment to subgroup 3, D = probability of assignment to subgroup 4.

**Figure S2B.** Probability of subgroup assignment in the late model.


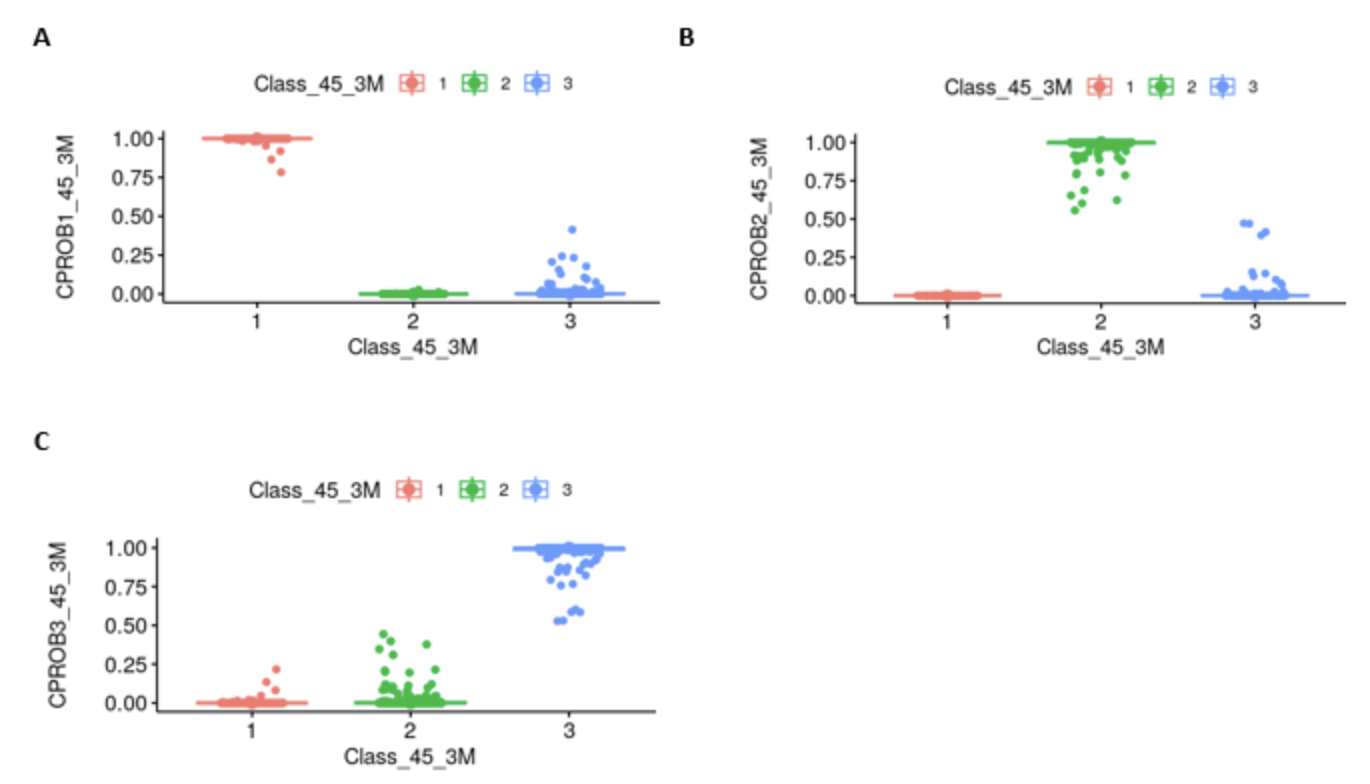


Probabilities of assignment to subgroup for each subgroup. A = Probability of assignment to Subgroup 1, B = probability of assignment to subgroup 2, C = probability of assignment to subgroup 3

**Figure S3A.** Density plot for class-defining variables of the latent profile analysis

**
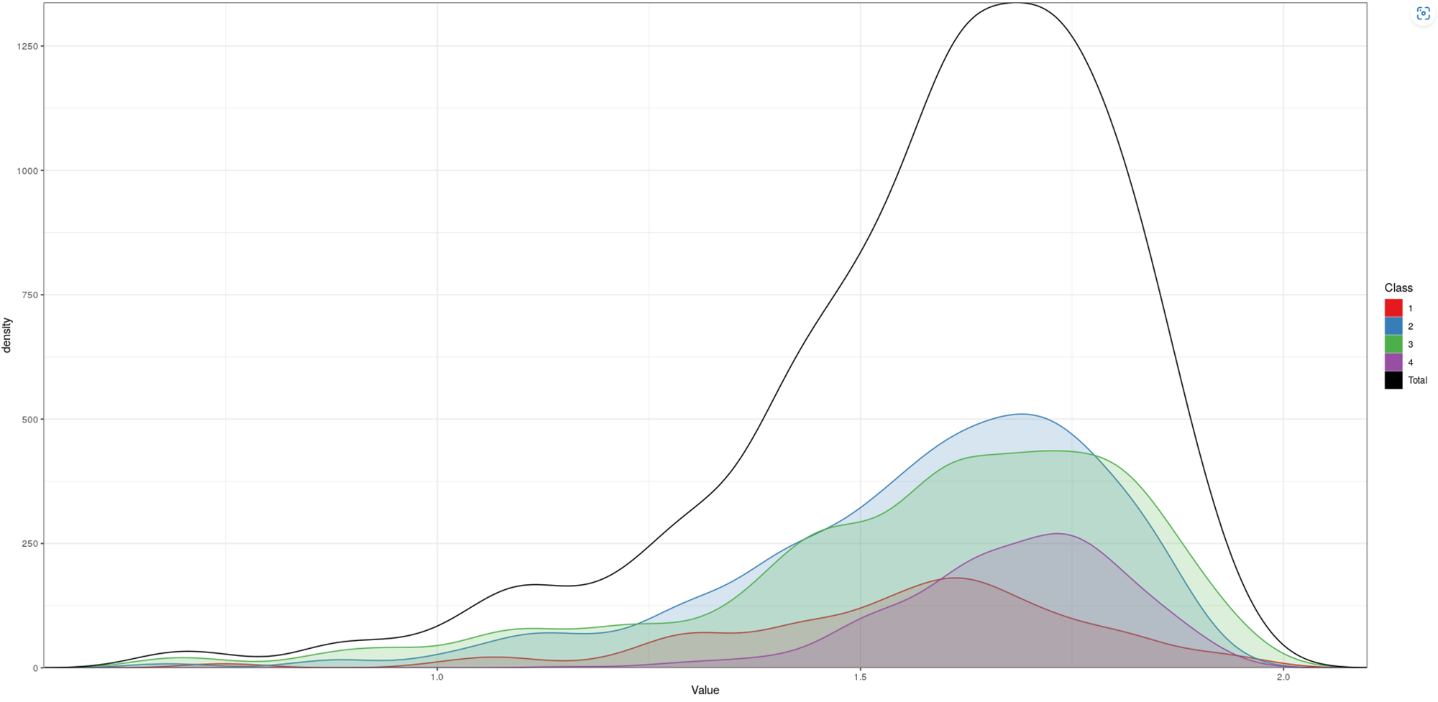
**

**Age**

**
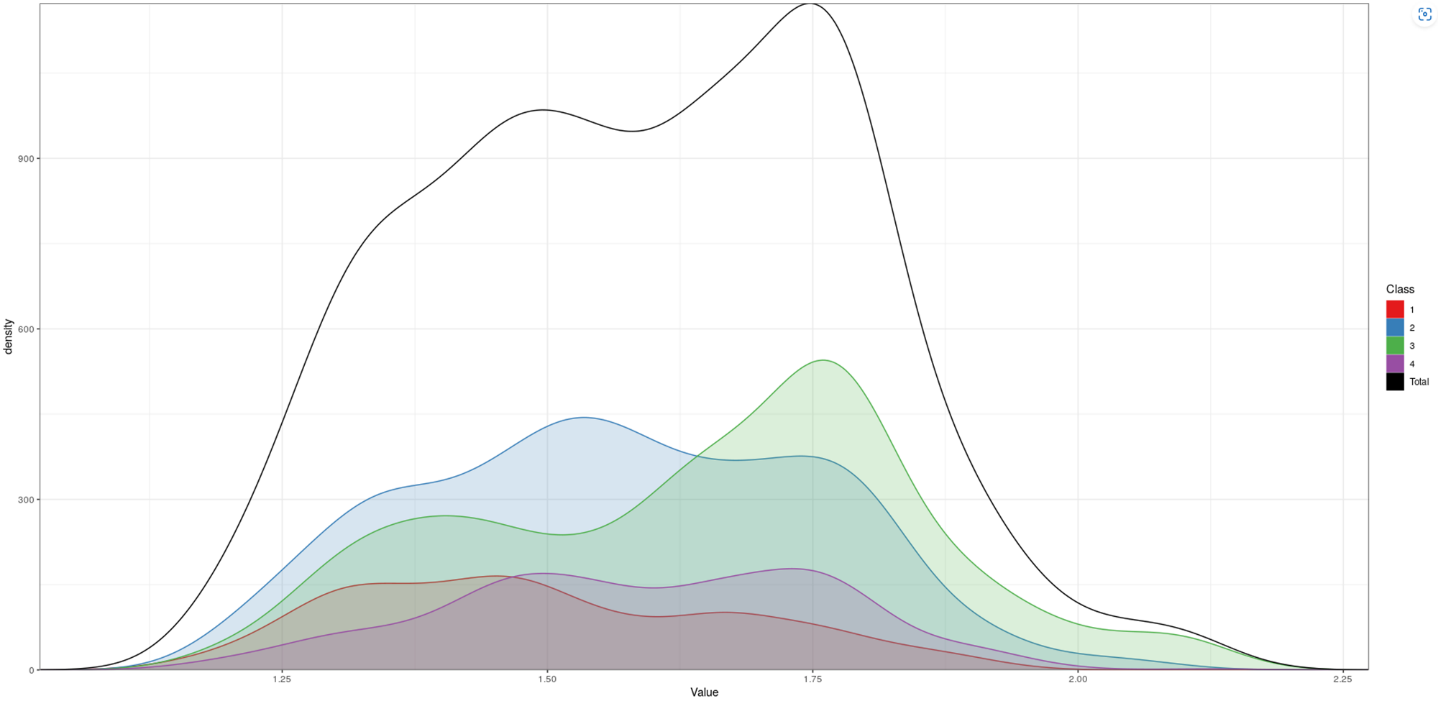
**

**CRP**

**CRP**

Black = density plot for the overall cohort; Coloured: separate density plots for each latent profile with cases weighted by the posterior propability of assignment to the respective profile. Variables were scaled and log-transformed.

**Figure S3B.** Density plot for class-defining variables of the latent profile analysis


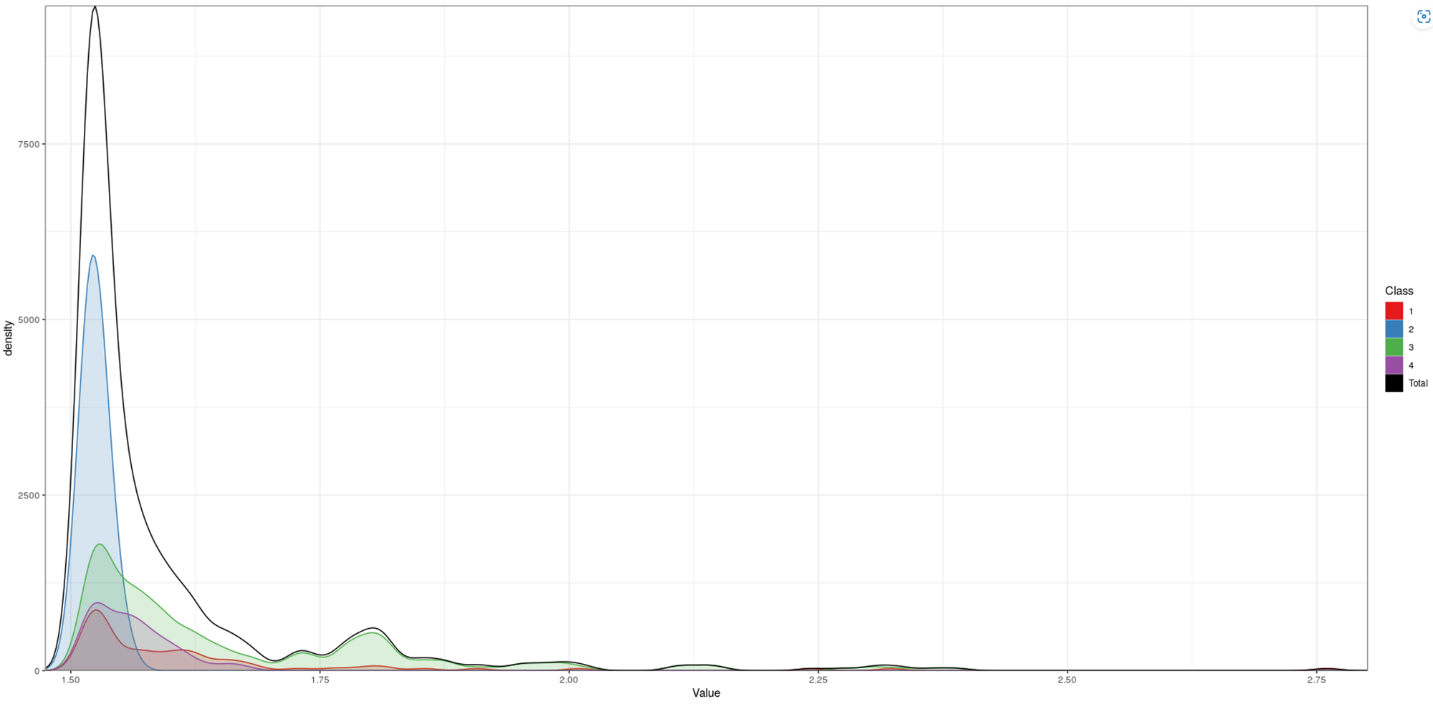


**PCT**

**
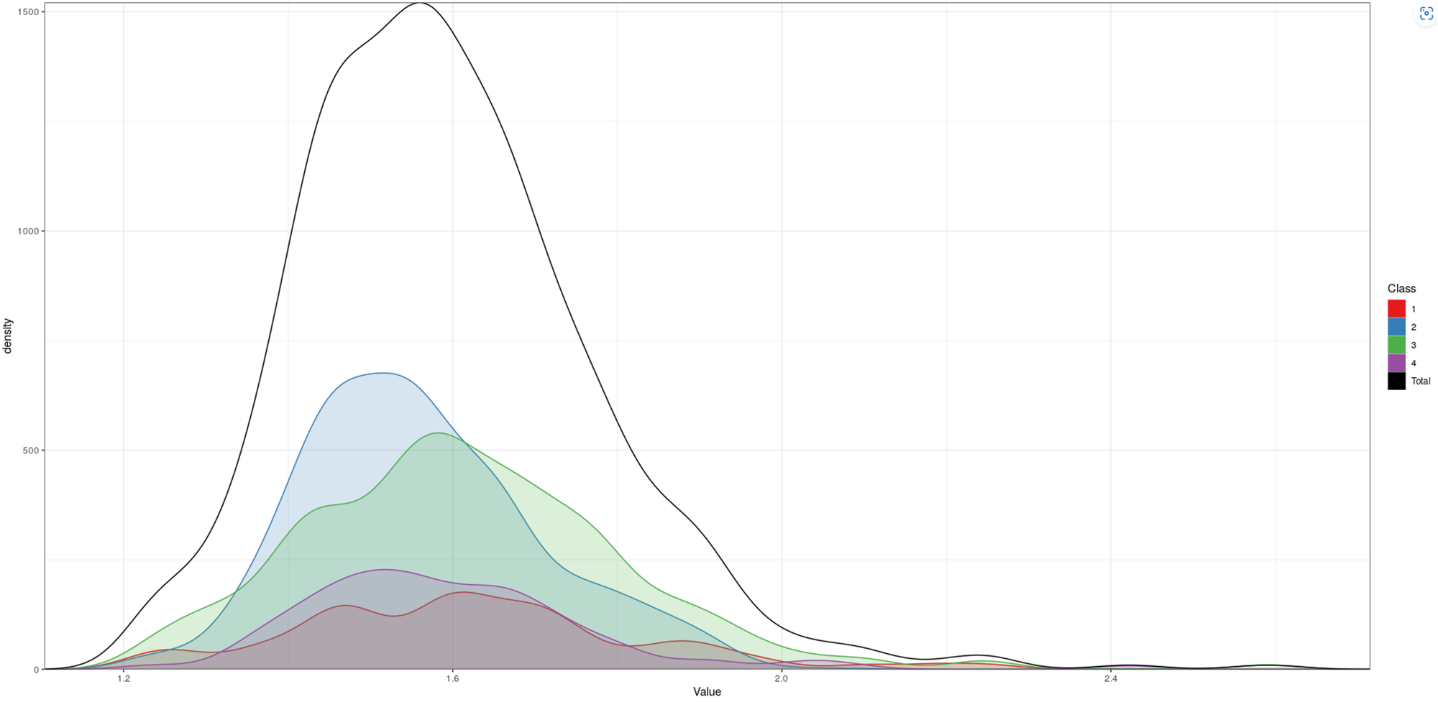
**

**WBC**

Black = density plot for the overall cohort; Coloured: separate density plots for each latent profile with cases weighted by the posterior propability of assignment to the respective profile. Variables were scaled and log-transformed.

**Figure S3C.** Density plot for class-defining variables of the latent profile analysis **
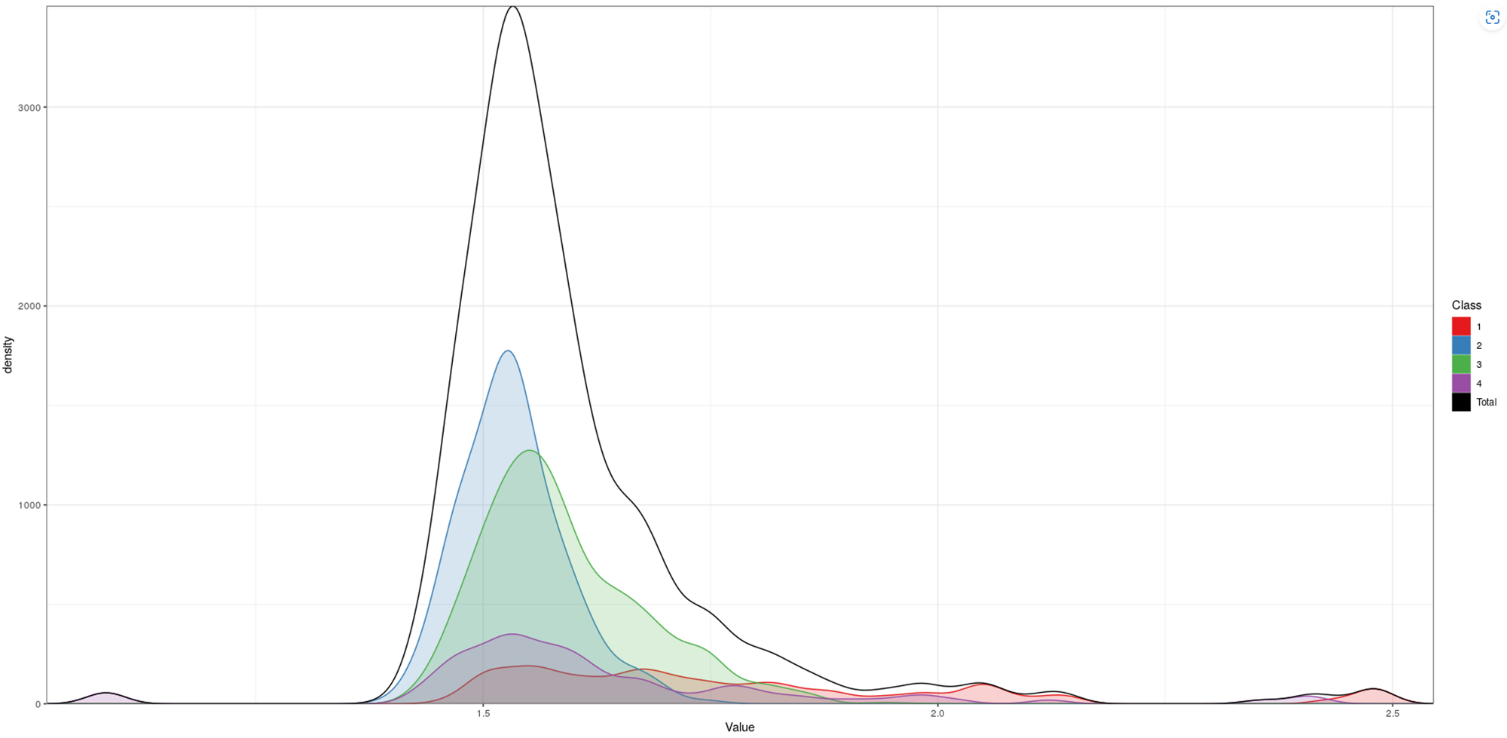
**

**INR**

**
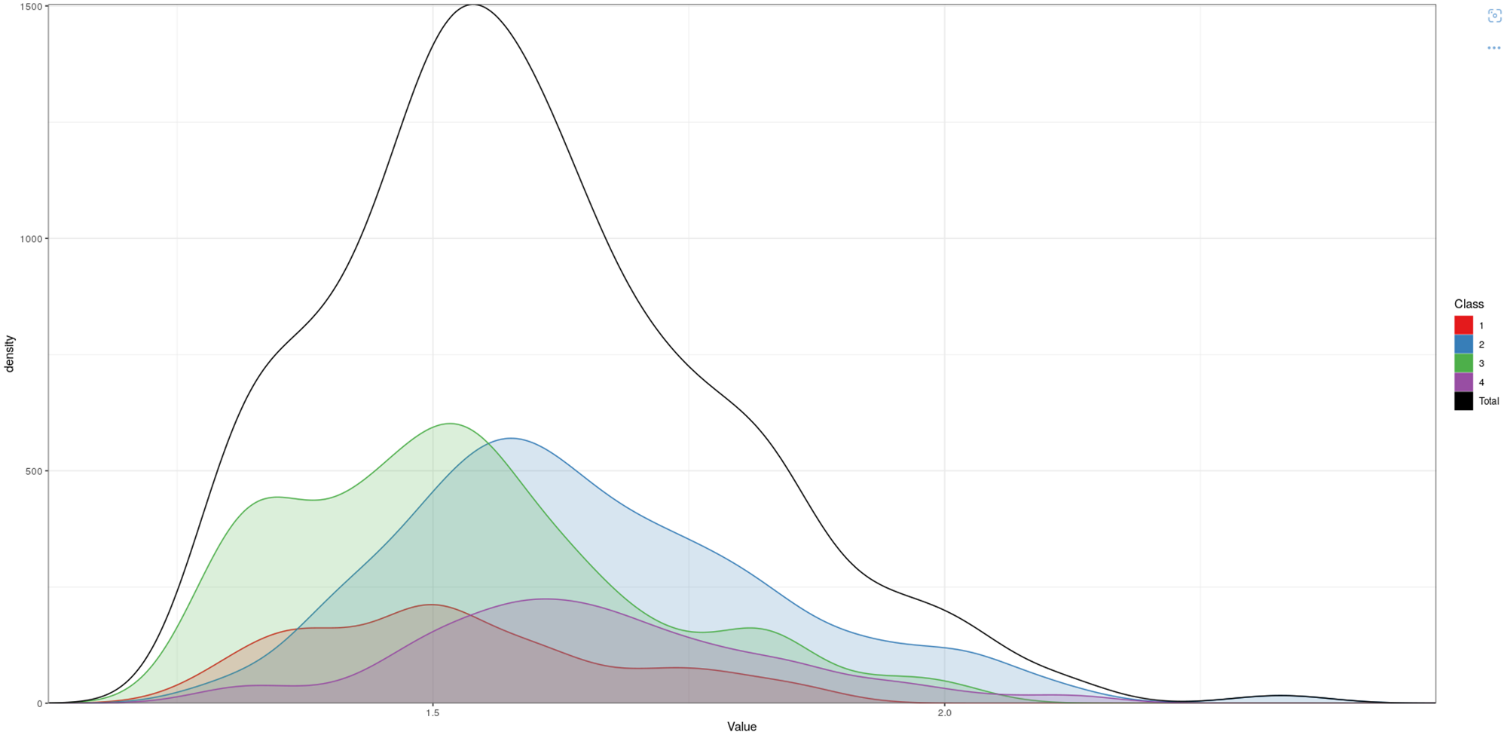
**

**Platelets**

Black = density plot for the overall cohort; Coloured: separate density plots for each latent profile with cases weighted by the posterior propability of assignment to the respective profile. Variables were scaled and log-transformed.

**Figure S3D.** Density plot for class-defining variables of the latent profile analysis


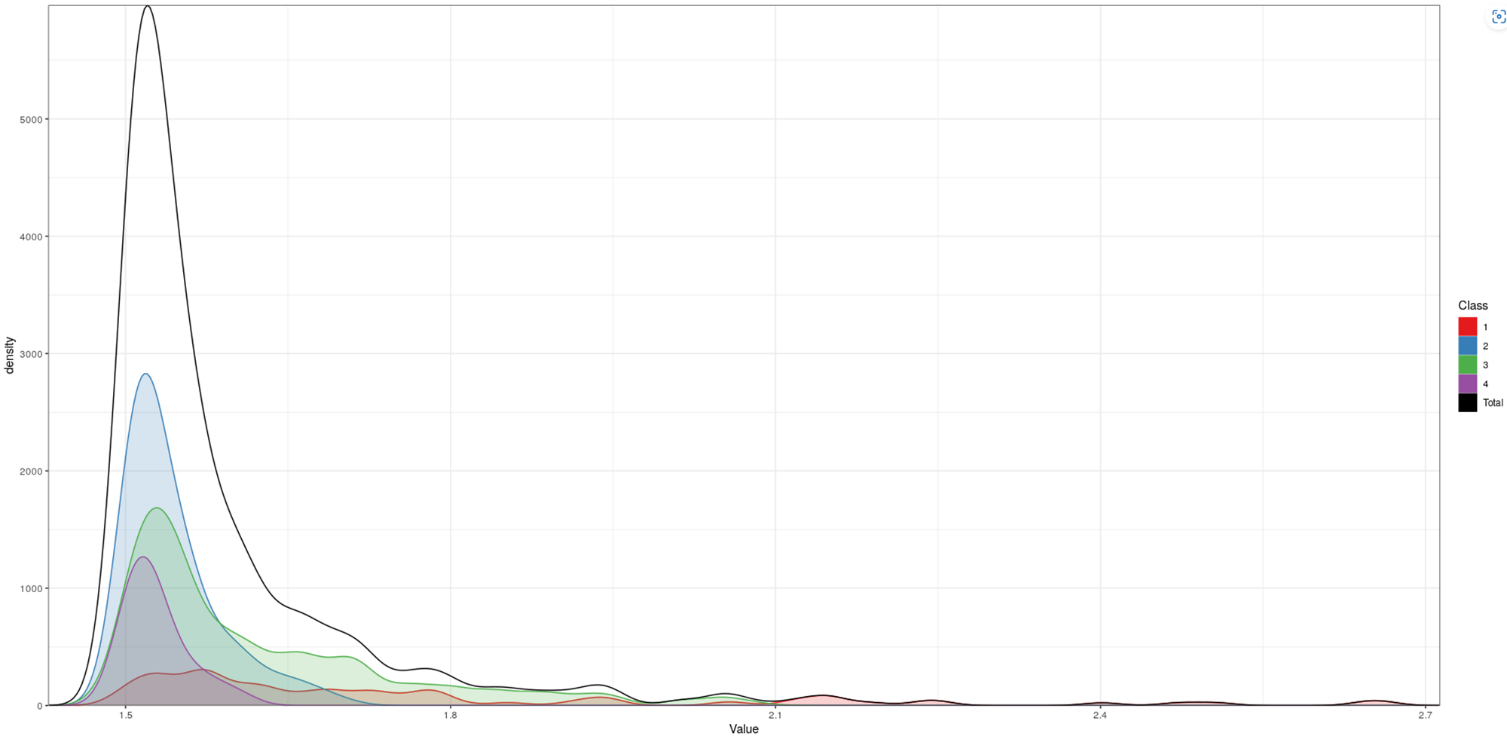


**Bilirubin**


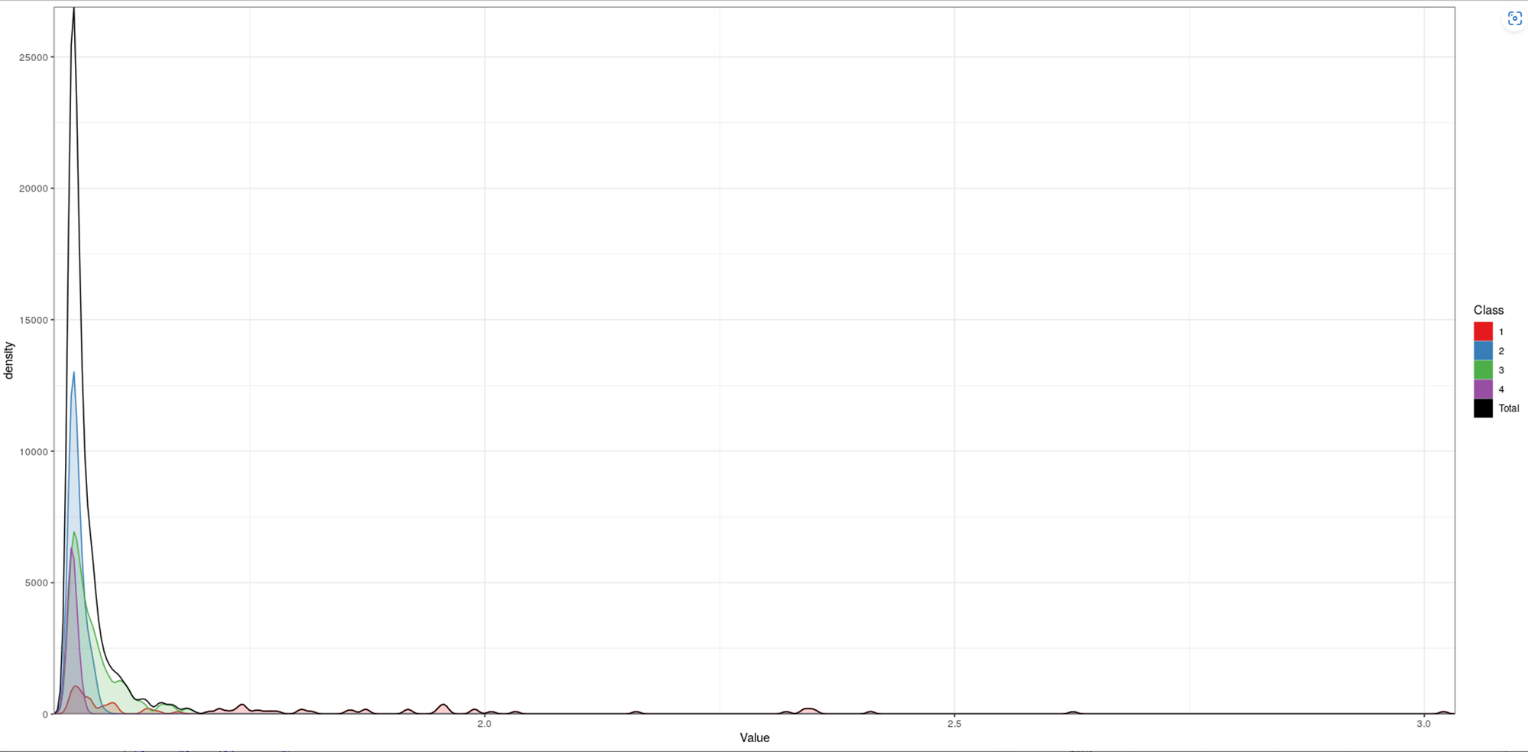


**ALT**

Black = density plot for the overall cohort; Coloured: separate density plots for each latent profile with cases weighted by the posterior propability of assignment to the respective profile. Variables were scaled and log-transformed.

**Figure S3E.** Density plot for class-defining variables of the latent profile analysis


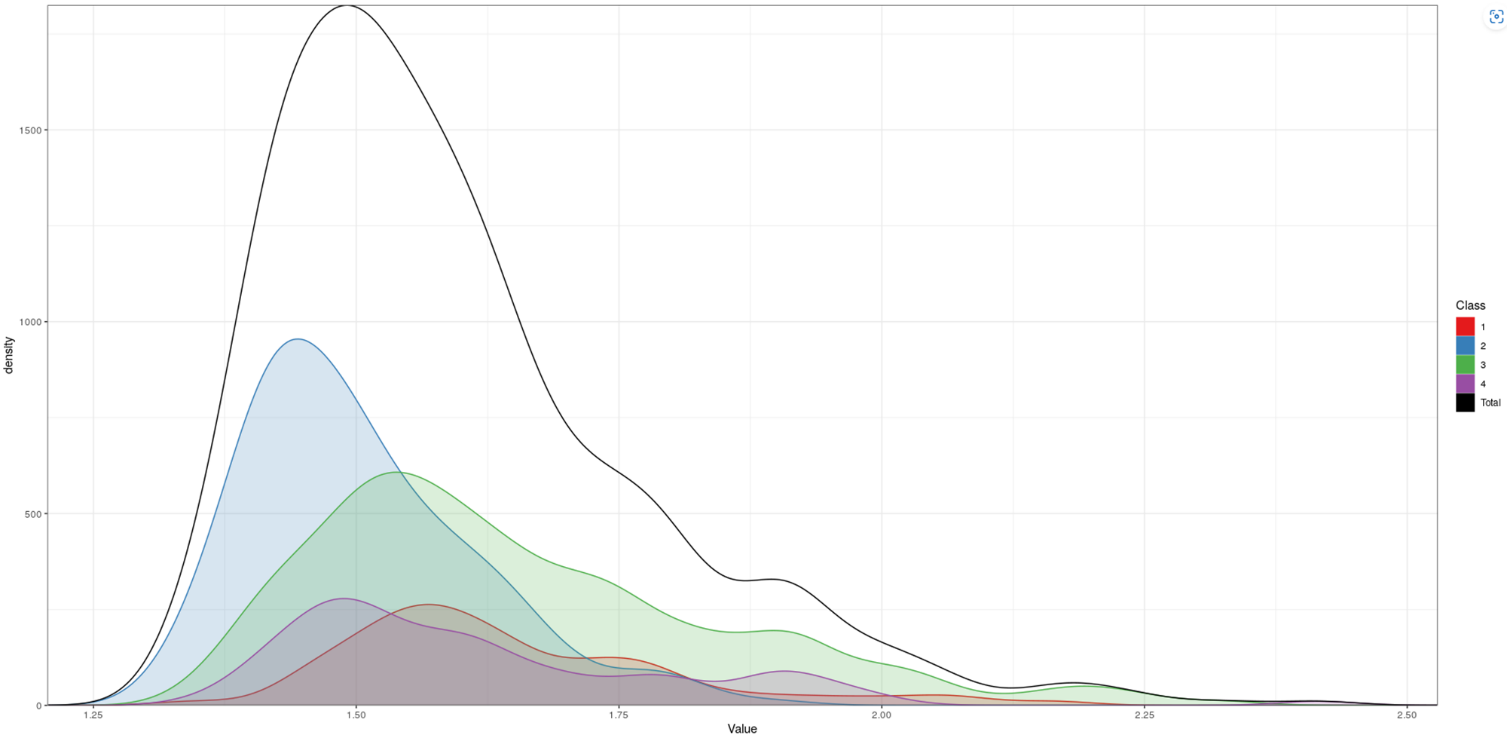


**Creatinine**


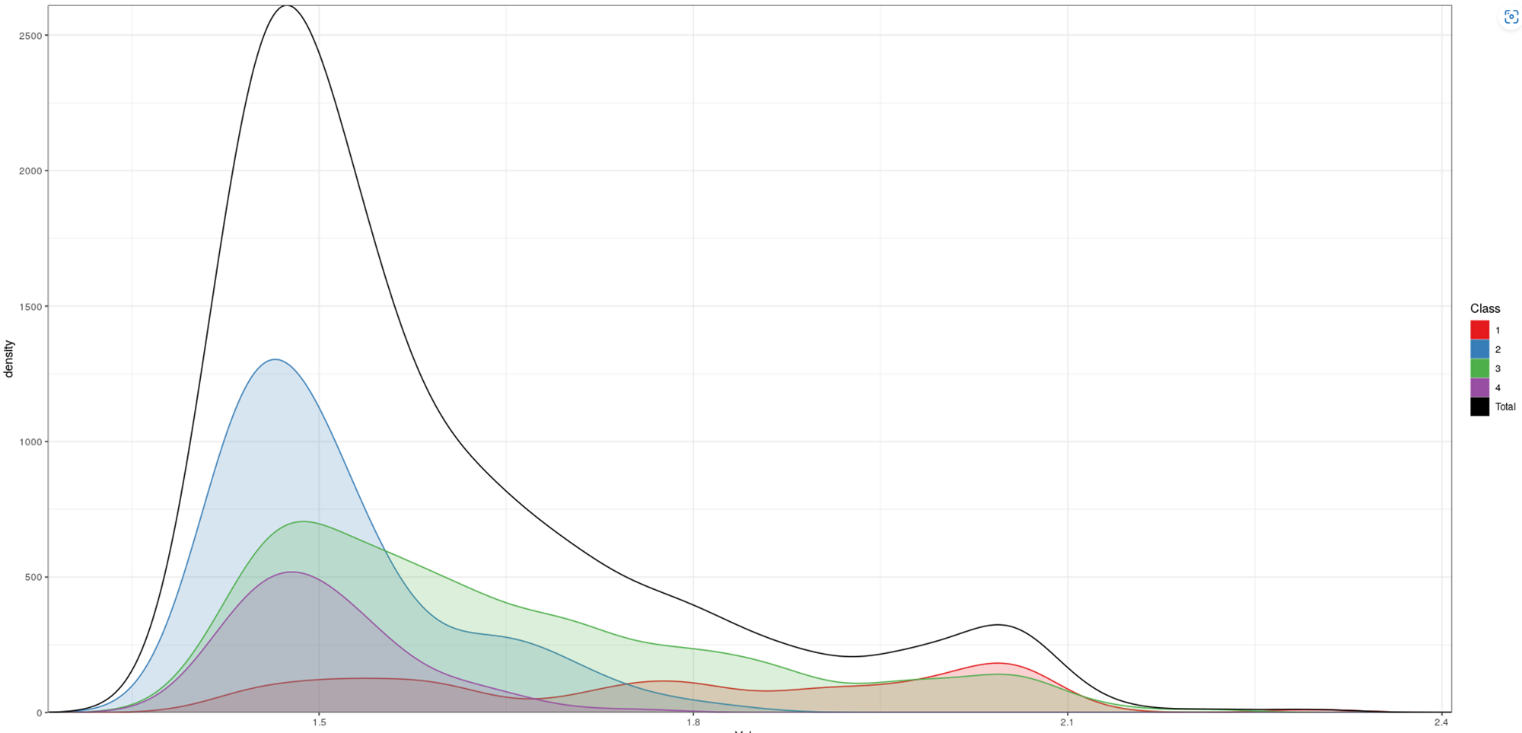


**Lactate**

Black = density plot for the overall cohort; Coloured: separate density plots for each latent profile with cases weighted by the posterior propability of assignment to the respective profile. Variables were scaled and log-transformed.

**Figure S3F.** Density plot for class-defining variables of the latent profile analysis


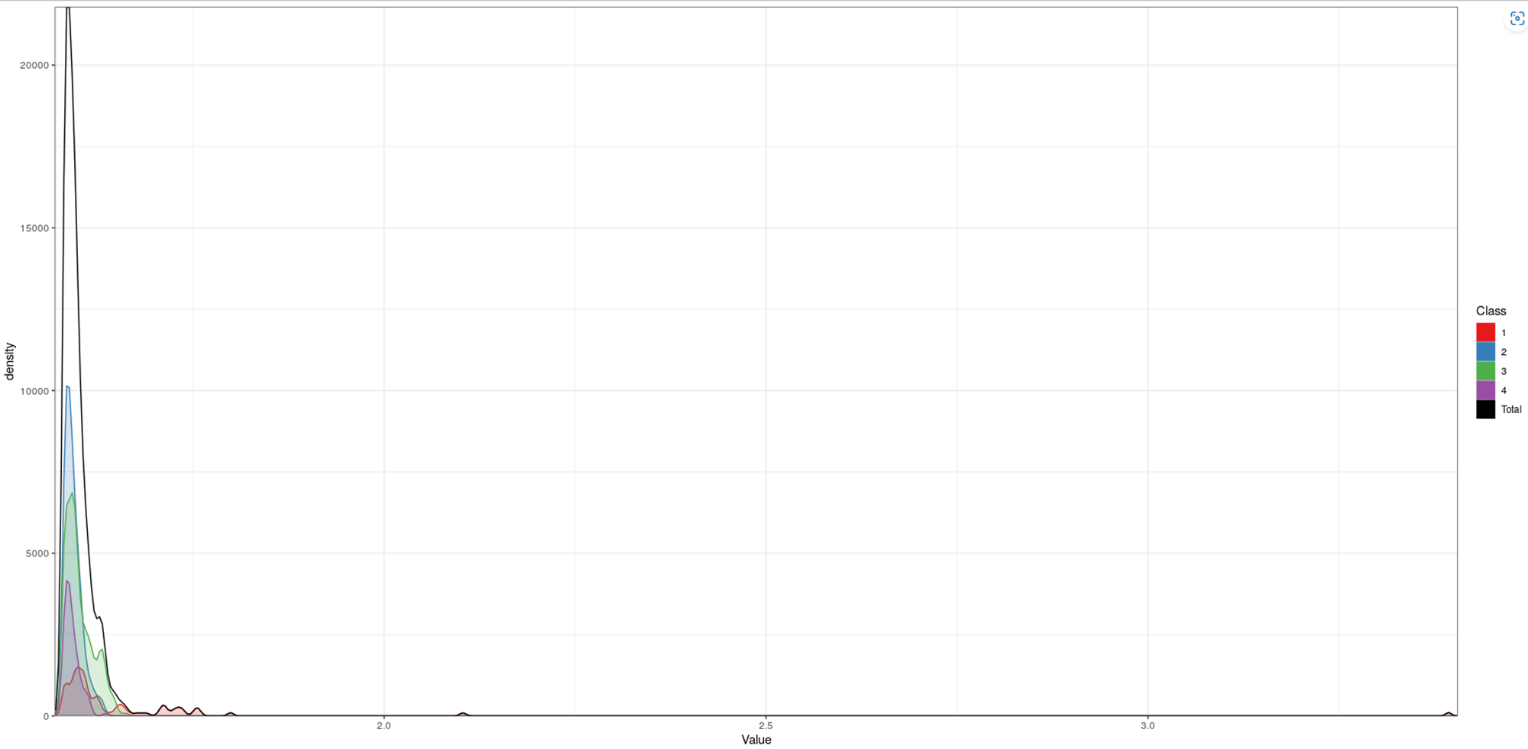


**Norepinephrine**

**rate**


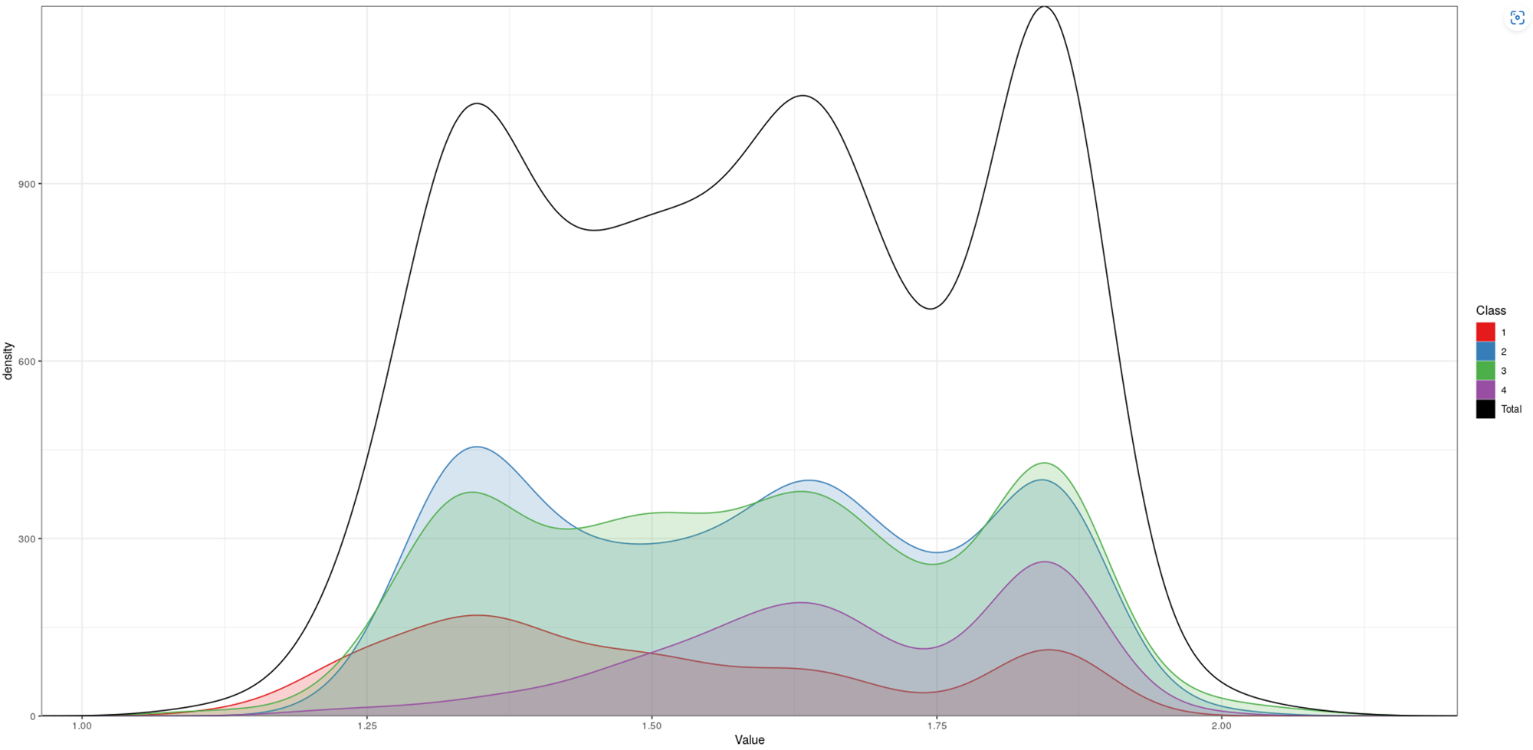


**P/F ratio**

Black = density plot for the overall cohort; Coloured: separate density plots for each latent profile with cases weighted by the posterior propability of assignment to the respective profile. Variables werere scaled and log-transformed.

**Table S3.** Variables not included in latent profile analysis

|  |  |  |  |  |  |
| --- | --- | --- | --- | --- | --- |
| Variable | Available values | 1  N = 76 | 2  N = 242 | 3  N = 236 | 4  N = 83 |
| IL-6 (max)  MD (IQR) | N = 118 | N = 17  501  (180 – 1703) | N = 47  185  (71 – 527) | N = 47  865 (277 – 9834) | N = 7  249 (145 – 379) |
| AST (max)  MD (IQR) | N = 522 | N = 73  1183  (235 - 3025) | N = 181  60  (34 -104) | N = 204  109  (51 – 332) | N = 64  33  (24 – 61) |
| Creatinine kinase (max)  MD (IQR) | N = 430 | N = 55  495  (155 – 1835) | N = 156  278 (81 – 930) | N = 171  348 (115 – 1461) | N = 48 81  (44 – 351) |
|  |  |  |  |  |  |

Variables are presented as Median (Interquartile range). *Abbreviations: IL-6 = interleukine-6, AST = aspartate aminotransferase, max = maximum value during the observation period of seven days*

**Figure S4.** Correlation matrix of candidate variables for latent profile analysis


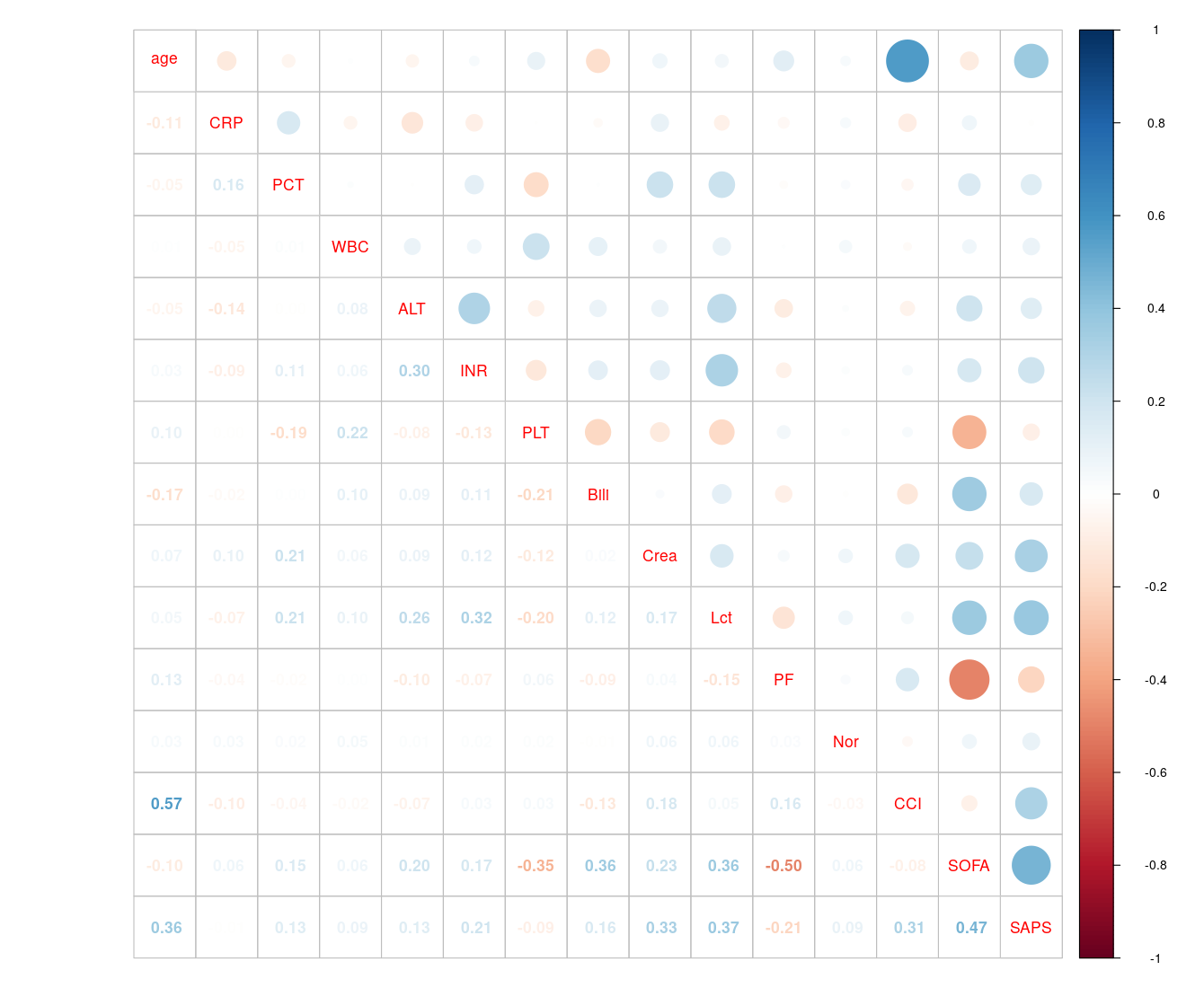


For the correlation matrix, the Pearson correlation coefficient of the maximum score within the first 48 hours after inclusion was calculated for variables that were candidates for latent profile analysis. *Abbreviations: CRP = c-reactive protein, PCT = procalcitonin, WBC = white blood cell count, ALT = alanine aminotransferase, INR = international normalized ratio of prothrombin time, PLT = platelets, Bili = bilirubin, Crea = creatinine, Lct = lactate, PF = ratio of arterial oxygen partial pressure and inspired fraction of oxygen, Nor = norepinephrine rate, CCI = Charlson comorbidity index, SOFA = sequential organ failure assessment, SAPS = simplified acute physiology score II*

**Figure S5.** Distribution of variables reflecting disease severity


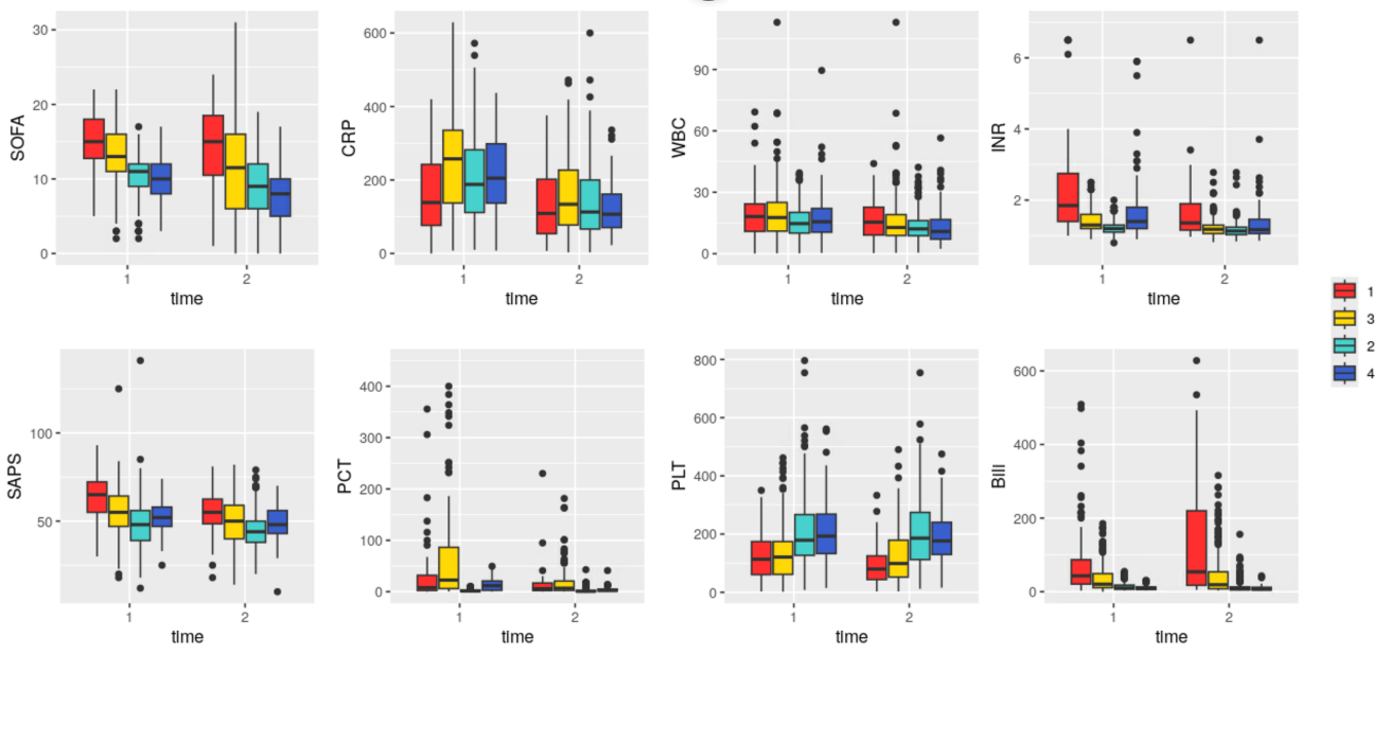


**
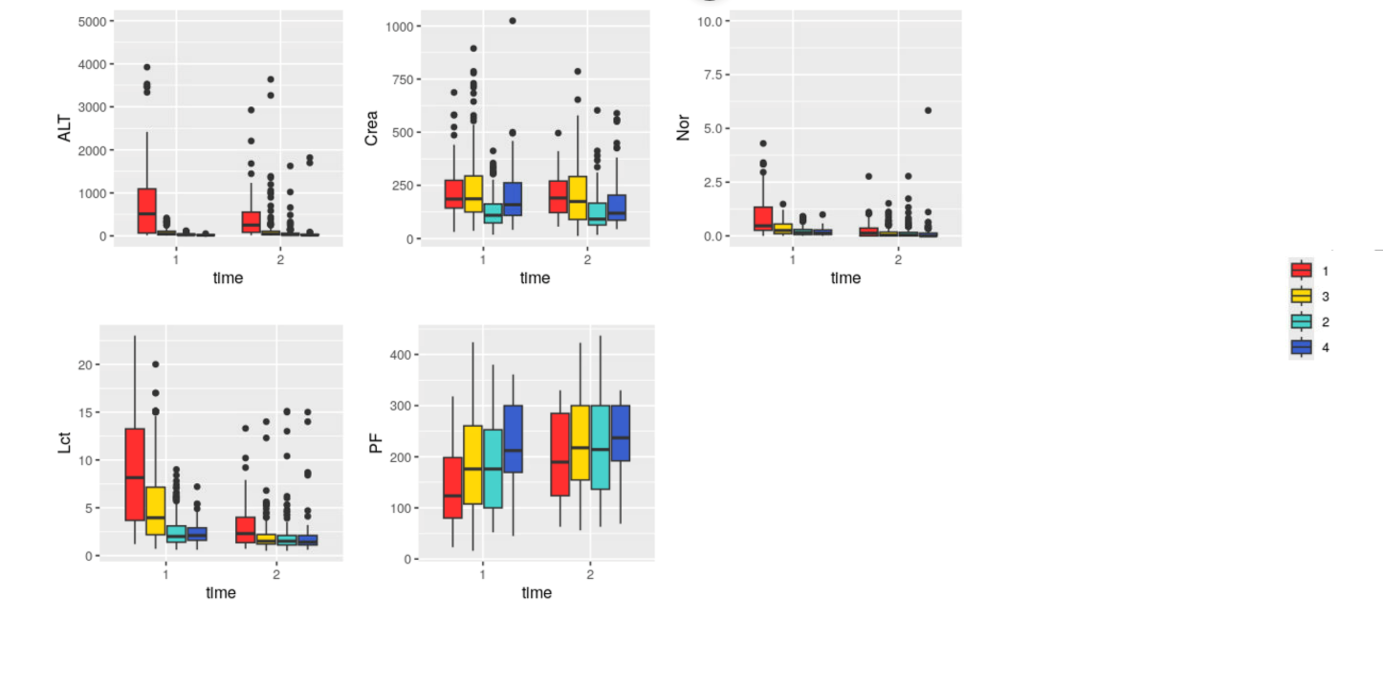
**

Distribution of class-defining variables and composite variables reflecting disease severity across the two time periods in which data were extracted, stratified by subgroups identified with the early model. 1 = within 48 hours of inclusion, 2 = within 120 – 144 hours of inclusion. *Abbreviations: CRP = c-reactive protein, PCT = procalcitonin, WBC = white blood cell count, ALT = alanine aminotransferase, INR = international normalized ratio of prothrombin time, PLT = platelets, Bili = bilirubin, Crea = creatinine, Lct = lactate, PF = ratio of arterial oxygen partial pressure and inspired fraction of oxygen, Nor = norepinephrine rate, SOFA = sequential organ failure assessment, SAPS = simplified acute physiology score II*

**Figure S6A.** Distribution of class-defining variables in the early model


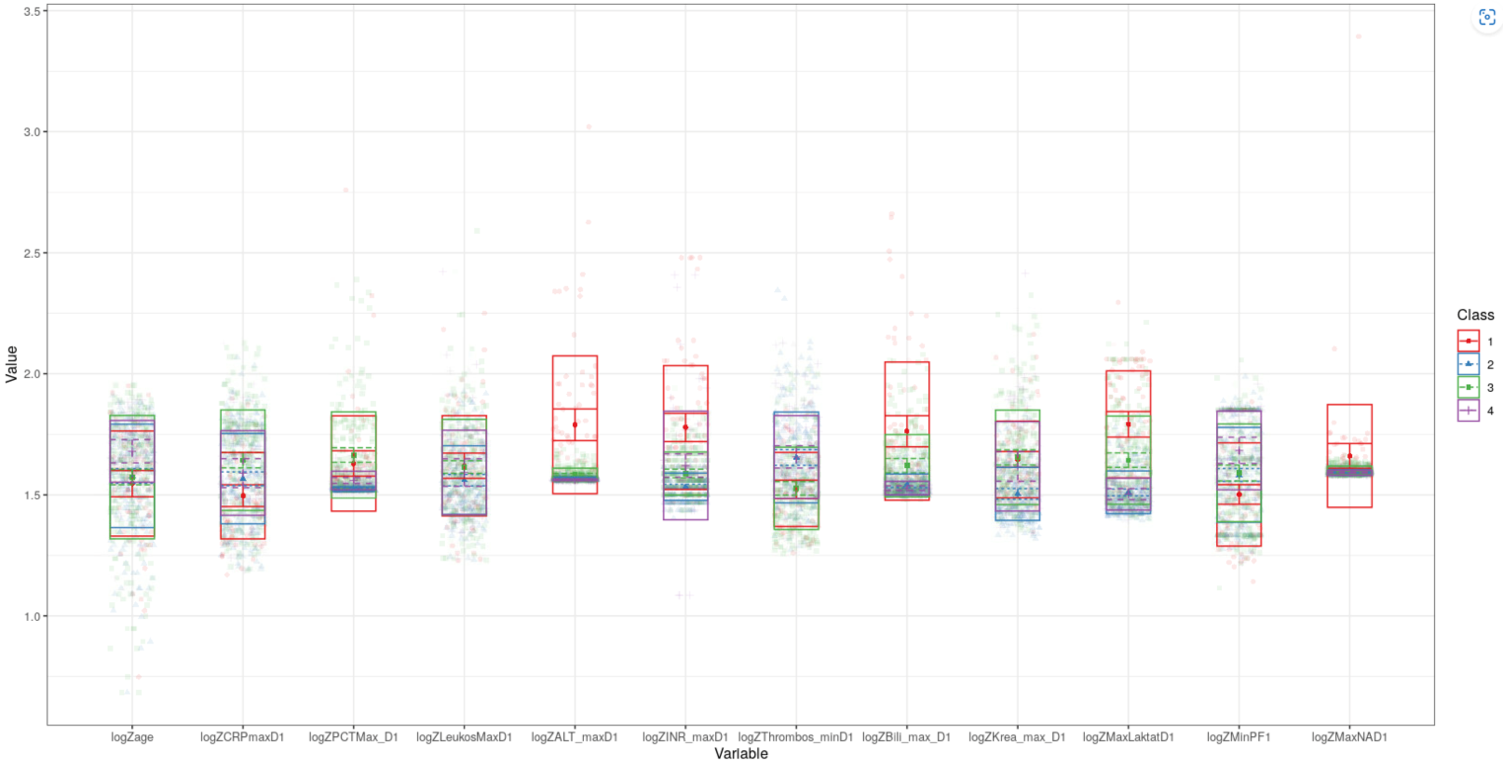


Variables were scaled and log-transformed.

**Figure S6B.** Distribution of class-defining variables in the late model


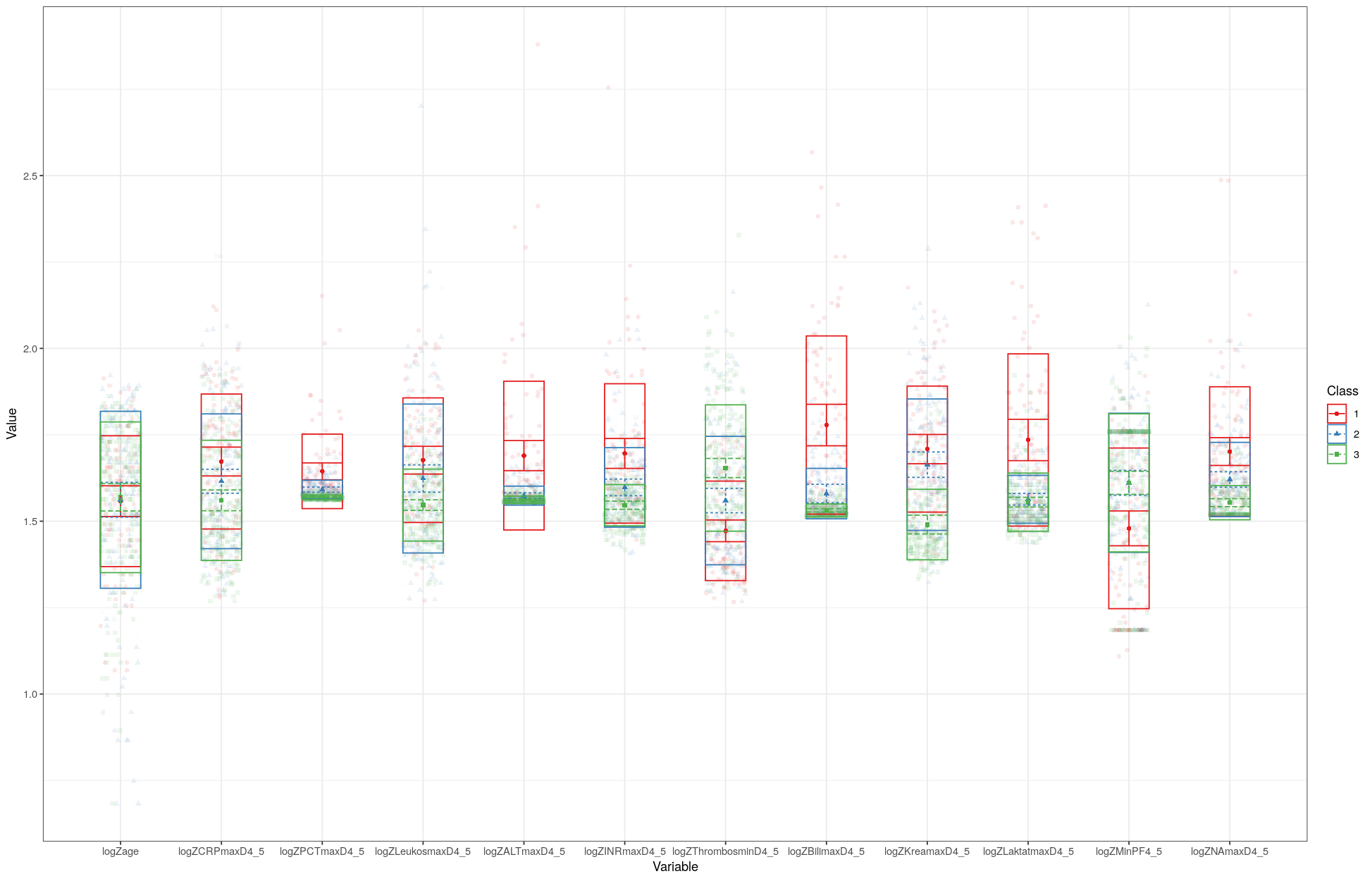


Variables were scaled and log-transformed. The numeration of the classes is randomly determined by the latent profile algorithm.

**Table S4. Detailed description of the history of pre-existing conditions**

|  |  |  | Subgroups |  |  |
| --- | --- | --- | --- | --- | --- |
| Variable | Overall  N = 637 | 1  N = 76 | 2  N = 242 | 3  N = 236 | 4  N = 83 |
| Coronary heart disease | 156 (24%) | 21 (28%) | 67 (28%) | 38 (16%) | 30 (36%) |
| Peripheral artery disease | 79 (12%) | 10 (13%) | 28 (12%) | 26 (11%) | 15 (18%) |
| Cerebrovascular disease | 122 (19%) | 10 (13%) | 51 (21%) | 41 (17%) | 20 (24%) |
| Dementia | 37 (6%) | 3 (4%) | 12 (5%) | 16 (7%) | 6 (7%) |
| Chronic kidney disease | 201 (32%) | 29 (38%) | 55 (23%) | 77 (33%) | 40 (48%) |
| Chronic Dialysis | 47 (7%) | 9 (12%) | 7 (3%) | 19 (8%) | 12 (14%) |
| Diabetes mellitus  Uncomplicated  With end organ damage | 49 (8%)  120 (19%) | 3 (4%)  10 (13%) | 31 (13%)  51 (21%) | 8 (3%)  35 (15%) | 7 (8%)  24 (29%) |
| COPD | 70 (11%) | 11 (14%) | 36 (15%) | 14 (6%) | 9 (11%) |
| Liver cirrhosis  mild  severe | 17 (2%)  15 (2%) | 1 (1%)  5 (7%) | 6 (3%)  2 (1%) | 7 (3%)  8 (3%) | 3 (4%)  0 |
| Metastatic neoplasm | 37 (6%) | 3 (4%) | 13 (5%) | 10 (4%) | 11 (13%) |
| Leukemia | 12 (2%) | 1 (1%) | 4 (2%) | 5 (2%) | 2 (2%) |
| Lymphoma | 25 (4%) | 2 (3%) | 6 (3%) | 11 (5%) | 6 (7%) |
| Solid neoplasm | 110 (17%) | 15 (20%) | 37 (15%) | 40 (17%) | 18 (22%) |
| Collagenosis | 12 (2%) | 2 (3%) | 5 (2%) | 4 (2%) | 1 (1%) |
| AIDS | 6 (1%) | 0 | 3 (1%) | 3 (1%) | 0 |
| Chronic steroid medication | 79 (12%) | 7 (9%) | 23 (10%) | 37 (16%) | 12 (14%) |
| Immunosuppression | 78 (12%) | 7 (9%) | 23 (10%) | 38 (16%) | 10 (12%) |
| Post Organ transplantation | 43 (7%) | 6 (8%) | 8 (3%) | 22 (9%) | 7 (8%) |
| Chronic heart failure  *missing* | 206 (32%)  *1* | 29 (38%) | 78 (32%) | 65 (28%)  *1* | 34 (41%) |
| Gastric ulcer | 33 (5%) | 2 (3%) | 11 (5%) | 15 (6%) | 5 (6%) |
| Hemiplegia | 68 (11%) | 5 (7%) | 28 (12%) | 25 (11%) | 10 (12%) |
| Neutropenia | 7 (1%) | 0 | 3 (1%) | 3 (1%) | 1 (1%) |
| Left-ventricular assist device | 16 (3%) | 5 (7%) | 4 (2%) | 4 (2%) | 3 (4%) |
|  |  |  |  |  |  |

Categorical variables are presented as Frequency (%). *Abbreviations: COPD = chronic obstructive pulmonary disease, AIDS = Acquired immune deficiency syndrome.* Where variables had missing values, these were listed in the table.

**Table S5. Baseline Characteristics**

|  |  |  |  |  | Subgroups |  |  |  |  |
| --- | --- | --- | --- | --- | --- | --- | --- | --- | --- |
|  | Overall |  | 1 |  | 2 |  | 3 |  | 4 |
|  | Early N = 637 | Late  N = 446 | Early  N = 76 | Late  N = 91 | Early  N = 242 | Late  N = 203 | Early  N = 236 | Late  N = 152 | Early  N = 83 |
| Age | 65 (54, 74) | 63 (52, 73) | 62 (52, 69) | 62 (52, 71) | 64 (53, 74) | 64 (52, 73) | 65 (52, 77) | 64 (53, 74) | 70 (63, 77) |
| Sex |  |  |  |  |  |  |  |  |  |
| - Male | 420 (66%) | 296 (66%) | 47 (62%) | 61 (67%) | 159 (66%) | 130 (64%) | 157 (67%) | 105 (69%) | 57 (69%) |
| BMI | 27 (24, 31) *missing: 6* | 28 (24, 31)  *missing: 6* | 28 (24, 33)  *missing: 0* | 28 (25, 32)  *missing: 0* | 27 (24, 32)  *missing: 2* | 28 (24, 32)  *Missing: 5* | 26 (23, 31) *missing: 1* | 26 (24, 31)  *missing: 1* | 26 (24, 31) *missing: 3* |
| CCI | 5 (3, 8) | 5 (2, 8) | 4 (2, 7) | 4 (1, 8) | 5 (2, 7) | 45(2, 7) | 5 (2, 8) | 6 (2, 9) | 7 (5, 9) |
| Disease severity at inclusion |  |  |  |  |  |  |  |  |  |
| - SOFA | 11 (8, 13) | 12 (10, 14) | 14 (10, 17) | 15 (12, 16) | 10 (7, 12) | 10 (8, 12) | 12 (9, 15) | 12 (9, 14) | 9 (7, 12) |
| - SAPS II | 50 (43, 60)  *missing: 40* | 52 (45, 61)  *missing: 3* | 62 (51, 71)  *missing: 9* | 56 (49, 66)  *1* | 47 (37, 54)  *missing: 5* | 47 (39, 53)  *2* | 53 (44, 63)  *missing: 25* | 53 (45, 60) | 51 (45, 57)  *missing: 1* |
| Focus |  |  |  |  |  |  |  |  |  |
| - Pulmonary | 279 (44%) | 228 (51%) | 32 (42%) | 46 (51%) | 132 (55%) | 116 (57%) | 84 (36%) | 66 (43%) | 31 (37%) |
| - Gastrointestinal | 58 (9%) | 40 (9%) | 15 (20%) | 12 (13%) | 16 (7%) | 16 (8%) | 19 (8%) | 12 (8%) | 8 (10%) |
| - Urogenital | 138 (22%) | 75 (17%) | 10 (13%) | 14 (15%) | 36 (15%) | 33 (16%) | 65 (28%) | 28 (18%) | 27 (33%) |
| - Cardiovascular | 102 (16%) | 72 (16%) | 18 (24%) | 14 (15%) | 34 (14%) | 29 (14%) | 35 (15%) | 29 (19%) | 15 (18%) |
| - Tissue | 57 (9%) | 35 (8%) | 3 (4%) | 6 (7%) | 22 (9%) | 15 (7%) | 28 (12%) | 14 (9%) | 4 (5%) |
| - Other* | 31 (5%) | 24 (5%) | 1 (1%) | 4 (4%) | 14 (6%) | 13 (6%) | 10 (4%) | 7 (5%) | 6 (7%) |
| Pathogen |  |  |  |  |  |  |  |  |  |
| - Gram positive bacteria | 287 (45%) | 215 (48%) | 33 (43%) | 43 (47%) | 115 (48%) | 95 (47%) | 98 (42%) | 77 (51%) | 41 (49%) |
| - Gram-negative bacteria | 320 (50% | 218 (49% | 32 (42%) | 38 (42%) | 125 (52%) | 108 (53%) | 114 (48%) | 72 (47%) | 49 (59%) |
| - Virus - SARS-CoV-2 | 98 (15%)  77 (12%) | 9072 (16%) (20%) | 9 (12%)  9 (12%) | 26 (29%)  23 (25%) | 44 (18%)  37 (15%) | 36 (18%)  25 (12%) | 40 (17%)  28 (12%) | 28 (18%)  24 (16%) | 5 (6%)  3 (4%) |
| - Culture negative | 94 (15%) | 18 (24%) | 18 (24%) | 13 (14%) | 28 (12%) | 26 (13%) | 37 (16%) | 21 (14%) | 11 (13%) |
| - Fungus | 55 (9%) | 48 (11%) | 6 (8%) | 14 (15%) | 22 (9%) | 21 (10%) | 15 (6%) | 13 (9%) | 12 (14%) |
| Infection characteristics |  |  |  |  |  |  |  |  |  |
| - Bacteremia | 265 (42%) | 188 (42%) | 35 (46%) | 42 (46%) | 85 (35%) | 73 (35%) | 105 (44%) | 74 (49%) | 40 (48%) |
| - Candidemia | 24 (4%) | 21 (5%) | 4 (5%) | 9 (20%) | 10 (4%) | 6 (3%) | 9 (4%) | 6 (4%) | 1 (1%) |
| - Secondary sepsis | 147 (23%) | 110 (25%) | 16 (21%) | 23 (25%) | 55 (23%) | 43 (21%) | 54 (23%) | 44 (29%) | 22 (27%) |
| - Multidrug resistent pathogen | 134 (21%) | 95 (21%) | 12 (16%) | 19 (21%) | 46 (19%) | 42 (21%) | 52 (22%) | 34 (22%) | 24 (29%) |
| Organ failure characteristics |  |  |  |  |  |  |  |  |  |
| - Septic shock | 535 (84%) | 386 (87%) | 73 (96%) | 89 (98%) | 192 (79%) | 159 (78%) | 205 (87%) | 138 (91%) | 65 (78%) |
| - Acute kidney injury* |  |  |  |  |  |  |  |  |  |
| - 0 | 157 (25%) | 111 (25%) | 4 (5%) | 3 (3%) | 98 (41%) | 87 (43%) | 35 (15%) | 21 (14%) | 20 (24%) |
| - 1 | 115 (18%) | 82 (18%) | 10 (13%) | 6 (7%) | 45 (19%) | 47 (23%) | 37 (16%) | 29 (19%) | 23 (28%) |
| - 2 | 84 (13%) | 61 (14%) | 6 (8%) | 9 (10%) | 37 (15%) | 34 (17%) | 31 (13%) | 18 (12%) | 10 (12%) |
| - 3   *missing* | 280 (44%)  1 | 191 (43%)  1 | 56 (74%) | 73 (80%) | 61 (25%)  1 | 34 (17%)  1 | 133 (56%) | 84 (55%) | 30 (36%) |
| - ARDS | 218 (34%) | 185 (41%) | 34 (45%) | 58 (64%) | 77 (32%) | 60 (30%) | 93 (39%) | 67 (44%) | 14 (17%) |
| - Thrombocyto-penia** | 265 (42%) | 200 (45%) | 44 (58%) | 65 (71%) | 68 (28%) | 56 (28%) | 135 (57%) | 79 (52%) | 18 (22%) |
| - Hyper-bilirubinemia | 288 (45%) | 213 (48%) | 63 (83%) | 73 (80%) | 70 (29%) | 57 (28%) | 140 (59%) | 83 (55%) | 15 (18%) |
| - HPD+DIC | 91 (14%) | 64 (14%) | 28 (37%) | 39 (43%) | 9 (4%) | 5 (3%) | 53 (22%) | 20 (13%) | 1 (1%) |
| - Delirium | 205 (32%) | 172 (39%) | 16 (21%) | 28 (31%) | 85 (35%) | 81 (40%) | 73 (31%) | 63 (41%) | 31 (37%) |
| - ContinuouslyRASS ≤ -4 | 210 (33%) | 141 (32%) | 46 (61%) | 47 (52%) | 67 (28%) | 48 (24%) | 84 (36%) | 46 (30%) | 13 (16%) |
| *missing* | *2* | *2* | *1* | *1* | *1* | *1* |  |  |  |
| - New onset of atrial fibrillation | 141 (26%) | 110 (29%) | 12 (20%) | 26 (36%) | 59 (28%) | 47 (27%) | 26 (27%) | 37 (28%) | 14 (24%) |
| *missing* | *104* | *72* | *16* | *19* | *32* | *31* | *32* | *22* | *24* |
|  |  |  |  |  |  |  |  |  |  |

Categorical variables are presented as Frequency (%). Continuous variables are presented as Median (Interquartile range). *Abbreviations: BMI = body mass index, CCI = Charlson Comorbidity Index, SOFA = Sequential organ failure assessment, SAPS II = Simplified Acute Physiology Score II, CRP = c-reactive protein, PCT = procalcitonin, WBC = white blood cell count, INR = international normalized ratio of prothrombin time, ALT = alanine aminotransferase, P/F ratio = ratio of partial pressure of arterial oxygen to fraction of inhaled oxygen. *“Other” included cases with meningoencephalitis, otorhinolaryngological or maxillofacial foci.* Where variables had missing values, these were listed in the table.

**Table S6A. Longitudinal characteristics of Subgroup 1**

|  |  |  |  |  | Subgroup |  |
| --- | --- | --- | --- | --- | --- | --- |
|  | Overall |  | 1 |  |  |  |
|  | Early  N = 76 | Late*  N = 91 | Stayed  N = 30 | Changed to 2  N = 2 | Changed to 3  N = 8 | Died  N = 31 |
| Subgroup-defining variables at the late time point |  |  |  |  |  |  |
| - CRP | 139 (75, 244) | 198 (100, 242) | 121 (51, 212) | 67 (58, 75) | 138 (68, 220) | - |
| - PCT   *missing* | 8 (2, 34)  *11* | 12 (5, 41)  *18* | 8 (4, 27)* | 5 (2, 8) | 2 (1, 4)* | - |
| - WBC | 18 (11, 24) | 19 (11, 25) | 19 (11, 25)* | 15 (8, 23) | 9 (6, 14)* | - |
| - PLT | 114 (61, 177) | 71 (44, 156) | 77 (32, 166) | 175 (71, 278) | 77 (69, 112) | - |
| - INR | 1.85 (1.40, 2.80) | 1.34 (1.2, 1.6) | 1.38 (1.17, 2.15) | 1.22 (1.04, 1.4) | 1.2 (1.1, 1.6) | - |
| - ALT   *missing* | 514 (67, 1,278)  *1* | 113( 50, 581)*4*  *4* | 305( 91, 608) | 61 (37, 85)* | 203 (32, 252) | - |
| - Bilirubin | 43 (21, 92) | 67 (14, 196) | 154 (37, 289)* | 9 (5, 12) | 18 (13, 33)* | - |
| - Creatinine | 186 (143, 278) | 219 (147, 334) | 202 (124, 265) | 67 (57, 77) | 171 (146, 267) | - |
| - PF ratio | 124 (80, 199) | 176 (86, 267) | 183 (117, 243)* | 245 (189, 300) | 296 (234, 300)* | - |
| - Lactate | 8.2 (3.7, 13.3) | 2.5 (1.4, 5.3) | 2.8 (1.6, 5.4)* | 2.8 (1.2, 4.4) | 1.35 (1,0, 2.1)* | - |
| - Norepinephrine rate | 0.48 (0.27, 1.40) | 0.23 (0.11, 0.49) | 0.23 (0.1, 0.56) | 0.1 (0.06, 0.08) | 0.08 (0, 0.25) | - |
|  |  |  |  |  |  |  |

Categorical variables are presented as Frequency (%). Continuous variables are presented as Median (Interquartile range). *p-value < .05, tested with Wilcoxon rank sum test, Fisher’s exact test or Wilcoxon rank sum exact test. 5 cases were missing in the repeated measures LPA due to right censoring. *Abbreviations: CRP = c-reactive protein, PCT = procalcitonin, WBC = white blood cell count, INR = international normalized ratio of prothrombin time, ALT = alanine aminotransferase, P/F ratio = ratio of partial pressure of arterial oxygen to fraction of inhaled oxygen.*

**Table S6B. Longitudinal characteristics of Subgroup 2**

|  |  |  |  |  | Subgroup |  |
| --- | --- | --- | --- | --- | --- | --- |
|  | Overall |  | 2 |  |  |  |
|  | Early  N = 242 | Late  N = 203 | Stayed N = 129 | Changed to 1 N = 11 | Changed to 3 N = 48 | Died N = 12 |
| Class-defining variables at day 4/5 |  |  |  |  |  |  |
| - CRP | 188 (111, 282) | 103 (63, 185) | 103 (63,182)* | 270 (204, 317)* | 122 (74, 219) | - |
| - PCT   *missing* | 1 (1, 3)  *20* | 2 (1, 10)  *27* | 0.5 (0.3 ,1)* | 7.4 (3.2,17)* | 3.2 (1.1, 5.1)* | - |
| - WBC | 15 (10, 20) | 11 (8, 16) | 11 (9 ,16) | 19 (13 ,26)* | 14 (11, 21) | - |
| - PLT | 179 (127, 268) | 180 (117, 270) | 208 (127 ,283) | 138 (45, 228)* | 142 (71, 261) | - |
| - INR | 1.20 (1.10, 1.30) | 1.12(1.03, 1.20) | 1.011(1.02,1.2)* | 1.46(1.26,2.43)* | 1.21 (1.06, 1.39) | - |
| - ALT   *missing* | 27 (18, 45)  *5* | 28 (19, 452  *10* | 31 (19 ,59) | 193 (48 661)* | 29 (19, 71) | - |
| - Bilirubin   *missing* | 10 (7, 18)  *1* | 7 (4, 12) | 7 (4 ,11) | 13 (11,66)* | 11 (7, 35)* | - |
| - Creatinine | 109 (73, 163) | 84 (61, 132) | 80 (57, 125)* | 229 (130, 260)* | 175 (93, 231)* | - |
| - PF ratio | 176 (100, 253) | 257 (186, 300) | 249 (178, 300) | 131 (80, 247)* | 214 (155, 297) | - |
| - Lactate | 2.0 (1.4, 3.1) | 1.40 (1.10, 1.90) | 1.4 (1.1, 1.9) | 5.3 (3, 13)* | 1.7 (1.2, 2.2) | - |
| - Norepinephrine rate | 0.16 (0.06, 0.30) | 0.01 (0.00, 0.1) | 0.03 (0.00,0.12) | 0.32 (0.2, 1.33)* | 0.19 (0.10, 0.35)* | - |
|  |  |  |  |  |  |  |

Categorical variables are presented as Frequency (%). Continuous variables are presented as Median (Interquartile range). *p-value < .05, tested with Wilcoxon rank sum test, Fisher’s exact test or Wilcoxon rank sum exact test. 30 cases were missing in the repeated measures LPA due to right censoring. *Abbreviations: CRP = c-reactive protein, PCT = procalcitonin, WBC = white blood cell count, INR = international normalized ratio of prothrombin time, ALT = alanine aminotransferase, P/F ratio = ratio of partial pressure of arterial oxygen to fraction of inhaled oxygen.*

**Table S6C. Longitudinal characteristics of Subgroup 3**

|  |  |  |  |  | Subgroup |  |
| --- | --- | --- | --- | --- | --- | --- |
|  | Overall |  | 3 |  |  |  |
|  | Early  N = 236 | Late  N = 152 | Stayed  N = 76 | Changed to 1  N = 46 | Changed to 2  N = 40 | Died  N = 38 |
| Class-defining variables at day 4/5 |  |  |  |  |  |  |
| - CRP | 258 (137, 336) | 134 (78, 234 | 160 (79, 256) | 202 (121, 242)* | 106 (62, 186)* | - |
| - PCT   *missing* | 23 (6, 87)  *18* | 4 (2, 12)  44 | 8 (2, 17) | 17 (7, 58)* | 3 (2, 4)* | - |
| - WBC | 18 (11, 25) | 13 (8, 20) | 13 (8, 120 | 17 (10, 24)* | 11 (8, 15) | - |
| - PLT | 121 (61, 176) | 114 (68, 193) | 88 (50, 165) | 56 (45, 111)* | 126 (80, 205)* | - |
| - INR | 1.30 (1.20, 1.60) | 1.2 (1.1, 1.4) | 1.17 (1.06, 1.30) | 1.29 (1.16, 1.49)* | 1.14 (1.05, 1.21) | - |
| - ALT   *missing* | 56 (26, 111)  *18* | 42 (21, 81)  *6* | 55 (28, 120) | 83 (43, 394)* | 29 (20, 48)* | - |
| - Bilirubin | 21 (11, 50) | 15 (7, 41) | 26 (10, 54) | 113 (14, 170)* | 12 (6, 19)* | - |
| - Creatinine | 187 (125, 295) | 189 (113, 294) | 198 (107, 323) | 258 (174, 397)* | 83 (64, 139)* | - |
| - PF ratio | 176 (107, 261) | 247 (180, 300) | 256 (200, 300) | 188 (80, 295)* | 277 (193, 300) | - |
| - Lactate | 4.0 (2.2, 7.2) | 1.60 (1.20, 2.10) | 1.6 (1.2, 2.1) | 1.8 (1.2, 4.0)* | 1.4 (1.0, 1.95) | - |
| - Norepinephrine rate | 0.25 (0.10, 0.56) | 0.13 (0.02, 0.28) | 0.1 (0.0, 0.3) | 0.19 (0.08, 0.38)* | 0.0 (0.0, 0.03)* | - |
|  |  |  |  |  |  |  |

Categorical variables are presented as Frequency (%). Continuous variables are presented as Median (Interquartile range). *p-value < .05, tested with Wilcoxon rank sum test, Fisher’s exact test or Wilcoxon rank sum exact test. 36 cases were missing in the repeated measures LPA due to right censoring. *Abbreviations: CRP = c-reactive protein, PCT = procalcitonin, WBC = white blood cell count, INR = international normalized ratio of prothrombin time, ALT = alanine aminotransferase, P/F ratio = ratio of partial pressure of arterial oxygen to fraction of inhaled oxygen.*“Other” included cases with meningoencephalitis, otorhinolaryngological or maxillofacial foci.*

**Table S6D. Longitudinal characteristics of Subgroup 4**

|  |  |  | Subgroup |  |  |
| --- | --- | --- | --- | --- | --- |
|  | Overall |  |  |  |  |
|  | Early  N = 83 | Changed to 1  N = 4 | Changed to 2  N = 32 | Changed to 3  N = 20 | Died  N = 3 |
| Class-defining variables at day 4/5 |  |  |  |  |  |
| - CRP | 205 (137, 302) | 169 (114, 248)* | 180 (72, 185) | 107 (74, 170) | - |
| - PCT | 12 (3, 21)  *14* | 2.8 (1.3, 12.3) | 2.85(1.1, 2.9) | 3.2 (1.4, 5)* | - |
| - WBC | 16 (10, 22) | 27 (17, 37) | 11 (7, 15) | 15 (7, 23) | - |
| - PLT | 193 (131, 270) | 162 (91, 223) | 165 (128, 241) | 192 (156, 265) | - |
| - INR | 1.40 (1.10, 1.80) | 1.85 (1.42, 2.2)* | 1.14 (1.05, 1.18) | 1.35 (1.1, 1.83) | - |
| - ALT | 18 (13, 29)  *3* | 38 (15, 940)* | 22 (17, 34) | 23 (15, 36)* | - |
| - Bilirubin | 9 (6, 13) | 15 (13, 28)* | 6 (5, 8) | 8 (5, 12) | - |
| - Creatinine | 159 (108, 264) | 226 (104, 423) | 94 (77, 145)* | 214 (146, 403)* | - |
| - PF ratio | 212 (168, 300) | 164 (93, 264) | 266 (199, 300)* | 263 (206, 300) | - |
| - Lactate | 2.1 (1.6, 2.9) | 8.7 (8.5, 11.4)* | 1.8 (1.4, 2.1) | 1.4 (1.2, 2.2) | - |
| - Norepinephrine rate | 0.13 (0.06, 0.29) | 0.53 (0.41, 0.87)* | 0.02 (0.0, 0.09) | 0.1 (0.01, 0.26) | - |
|  |  |  |  |  |  |

Categorical variables are presented as Frequency (%). Continuous variables are presented as Median (Interquartile range). *p-value < .05, tested with Wilcoxon rank sum test, Fisher’s exact test or Wilcoxon rank sum exact test. 24 cases were missing in the repeated measures LPA due to right censoring. *Abbreviations: CRP = c-reactive protein, PCT = procalcitonin, WBC = white blood cell count, INR = international normalized ratio of prothrombin time, ALT = alanine aminotransferase, P/F ratio = ratio of partial pressure of arterial oxygen to fraction of inhaled oxygen.* ** “Other” included cases with meningoencephalitis, otorhinolaryngological or maxillofacial foci.*

**Table S7. Multivariate regression analysis: Independent risk factors for in-hospital mortality**

| Variable | OR | 95% CI | p-value |
| --- | --- | --- | --- |
| Focus: Urogenital | 0.36 | 0.20, 0.60 | < .001 |
| Pathogen: gram-negative | 1.55 | 1.02, 2.38 | .042 |
| Age | 1.04 | 1.03, 1.06 | < .001 |
| Comorbidity: PAD | 2.86 | 1.56, 5.32 | < .001 |
| Comorbidity: CAD | 0.59 | 0.35, 0.96 | .037 |
| Comorbidity: COPD | 2.14 | 1.12, 4.12 | .033 |
| Subgroup 3 | 1.98 | 1.26, 3.13 | .003 |
| Subgroup 1 | 10.2 | 5.09, 21.7 | <.001 |
|  |  |  |  |

Variables included in the model: Age, body mass index, sex; focus: pulmonary, gastrointestinal, urogenital, head, or tissue; pathogen: grampositive, gramnegative, virus, culture negative, multidrug-resistent pathogen; subgroup assignments 1-4, comorbidities: Charlson comorbidity index, chronic heart failure, immunosuppression, liver cirrhosis, chronic obstructive pulmonary disease, coronary heart disease, peripheral artery disease, stroke, dementia; calculated antiinfective therapy with multidrug resistant coverage. *Abbreviations: CAD = coronary artery disease, PAD = peripheral arterial disease, COPD = chronic*

**Table S8A. Multivariate regression analysis: Predictors of subgroup assignment**

|  | OR | 95% CI | p-value |  |
| --- | --- | --- | --- | --- |
| Subgroup 1 |  |  |  | Pseudo-R^2^ = 0.83 |
| INR | 2.61 | 1.81, 3.92 | < .001 |  |
| Bilirubin | 3.38 | 2.12, 6.16 | < .001 |  |
| ALT | 487 | 72.4, 5,617 | < .001 |  |
| Lactate | 2.15 | 1.38, 4.42 | < .001 |  |
| PCT | 1.41 | 1.05, 1.97 | .030 |  |
| Subgroup 2 |  |  |  | Pseudo-R^2^ = 0.87 |
| WBC | 0.59 | 0.37, 0.93 | .025 |  |
| Lactate | 0.50 | 0.29, 0.83 | .009 |  |
| Creatinine | 0.23 | 0.14, 0.38 | < .001 |  |
| Bili | 0.17 | 0.06, 0.40 | < .001 |  |
| INR | 0.20 | 0.09, 0.40 | < .001 |  |
| ALT | 0.00 | 0.00, 0.00 | < .001 |  |
| PCT | 0.00 | 0.00, 0.00 | < .001 |  |
| Subgroup 3 |  |  |  | Pseudo-R^2^ = 0.57 |
| CRP | 1.49 | 1.18, 1.87 | <.001 |  |
| WBC | 1.49 | 1.17, 1.90 | .001 |  |
| PCT | 2.46 | 1.67, 3.84 | <.001 |  |
| Platelets | 0.53 | 0.40, 0.71 | <.001 |  |
| Creatinine | 1.61 | 1.28, 2.07 | < .001 |  |
| Lactate | 1.79 | 1.39, 2.35 | <.001 |  |
| INR | 0.43 | 0.26, 0.64 | <.001 |  |
| ALT | 0.32 | 0.13, 0.59 | .003 |  |
| SOFA Score D0/1 | 1.08 | 1.01, 1.17 | .035 |  |
| Subgroup 4 |  |  |  | Pseudo-R^2^= 0.58 |
| Age | 1.03 | 1.00, 1.05 | .049 |  |
| INR | 2.36 | 1.63, 3.62 | < .001 |  |
| Bilirubin | 0.08 | 0.01, 0.36 | .004 |  |
| ALT | 0.00 | 0.00, 0.00 | < .001 |  |
| Lactate | 0.24 | 0.10, 0.50 | < .001 |  |
| Creatinine | 1.45 | 1.07, 1.99 | .017 |  |
| P/F ratio | 1.86 | 1.28, 2.76 | .001 |  |

Multivariate logistic regression analysis with forward variable selection. All variables that were incorporated in the early latent profile analysis and the maximal SOFA score in the first 48 hours were included in this model. *Abbreviations: CRP = c-reactive protein, PCT = procalcitonin, WBC = white blood cell count, INR = international normalized ratio of prothrombin time, ALT = alanine aminotransferase, P/F ratio = ratio of partial pressure of arterial oxygen to fraction of inhaled oxygen.*

**Table S8B. Multivariate regression analysis: Predictors of subgroup assignment for the late model**

|  | OR | 95% CI | p-value |  |
| --- | --- | --- | --- | --- |
| Subgroup 1 |  |  |  | Pseudo-R^2^ = 0.99 |
| INR | 23.1 | 4.53, 215 | < .001 |  |
| PCT | 213,011 | 2,986, 77,674,135 | < .001 |  |
| Bilirubin | 221 | 28.3, 4,486 | < .001 |  |
| ALT | 3,890,937 | 16,921, 11,238,109,741 | < .001 |  |
| Lactate | 41.7 | 10, 289 | < .001 |  |
| Creatinine | 3.72 | 1.58, 10.6 | .006 |  |
| Age | 1.13 | 1.04, 1.25 | .005 |  |
| P/F ratio | 0.14 | 0.03, 0.45 | .003 |  |
| ∆SOFA | 0.67 | 0.48, 0.87 | .005 |  |
| Subgroup 2 |  |  |  | Pseudo-R^2^ = 0.99 |
| Age | 1.05 | 1.02, 1.09 | .003 |  |
| PCT | 0.00 | 0.00, 0.00 | < .001 |  |
| WBC | 0.2 | 0.08, 0.39 | <.001 |  |
| INR | 0.00 | 0.00, 0.03 | <.001 |  |
| Bilirubin | 0.00 | 0.00, 0.02 | <.001 |  |
| ALT | 0.00 | 0.0, 0.0 | <.001 |  |
| Creatinine | 0.08 | 0.03, 0.18 | < .001 |  |
| ∆SOFA | 0.56 | 0.42, 0.70 | < .001 |  |
| Lactate | 2.58 | 1.25, 4.95 | .005 |  |
| P/F ratio | 0.31 | 0.15, 0.57 | < .001 |  |
| Subgroup 3 |  |  |  | Pseudo-R^2^ = 0.89 |
| WBC | 1.85 | 1.44, 2.44 | <.001 |  |
| PCT | 0.08 | 0.03, 0.20 | < .001 |  |
| INR | 1.76 | 1.29, 2.41 | <.001 |  |
| Platelets | 0.58 | 0.44, 0.77 | <.001 |  |
| Bilirubin | 0.57 | 0.39, 0.77 | < .001 |  |
| ALT | 0.23 | 0.10, 0.46 | <.001 |  |
| Creatinine | 1.99 | 1.54, 2.61 | <.001 |  |
| Lactate | 0.31 | 0.19, 0.48 | < .001 |  |
| P/F ratio | 1.47 | 1.14, 1.9 | .003 |  |
| ∆SOFA | 1.16 | 1.07, 1.27 | < .001 |  |
|  |  |  |  |  |

Multivariate logistic regression analysis with forward variable selection. All variables that were incorporated in the late latent profile analysis and ∆SOFA were included in this model. *Abbreviations: CRP = c-reactive protein, PCT = procalcitonin, WBC = white blood cell count, INR = international normalized ratio of prothrombin time, ALT = alanine aminotransferase, P/F ratio = ratio of partial pressure of arterial oxygen to fraction of inhaled oxygen,* ∆SOFA = Sequential organ failure score at the late time point minus the corresponding score at the early time point.

**Table S9. Multivariate regression analysis: Risk factors for subgroup assignment based on patient and infection characteristics**

|  | OR | 95% CI | p-value |  |
| --- | --- | --- | --- | --- |
| Subgroup 1 |  |  |  | Pseudo-R^2^ = 0.14 |
| CCI | 0.88 | 0.77, 0.99 | 0.05 |  |
| Gastrointestinal focus | 2.89 | 1.28, 6.42 | 0.01 |  |
| Liver cirrhosis | 1.77 | 1.10, 2.79 | 0.015 |  |
| Chronic hemodialysis | 2.63 | 1.03, 6.34 | 0.035 |  |
| Subgroup 2 | 2.03 |  |  | Pseudo-R^2^ = 0.14 |
| Urogenital focus | 0.56 | 0.32, 0.97 | 0.040 |  |
| COPD | 2.03 | 1.16, 3.57 | 0.013 |  |
| Chronic hemodialysis | 0.25 | 0.10, 0.57 | 0.002 |  |
| Subgroup 3 |  |  |  | Pseudo-R^2^ = 0.14 |
| Pulmonary focus | 0.61 | 0.38, 0.95 | 0.032 |  |
| Tissue focus | 1.97 | 1.02, 3.82 | 0.043 |  |
| COPD | 0.47 | 0.24, 0.89 | 0.025 |  |
| CAD | 0.47 | 0.28, 0.79 | 0.005 |  |
| Subgroup 4 |  |  |  | Pseudo-R^2^= 0.20 |
| CCI | 1.21 | 1.09, 1.34 | <0.001 |  |
| Chronic Hemodialysis | 2.32 | 1.00, 5.17 | 0.043 |  |

Multivariate logistic regression analysis with forward variable selection. Variables included in the model were: Patient characteristics: age, CCI, sex, body mass index; Infection characteristics: pulmonary focus, gastrointestinal focus, urogenital focus, tissue focus, other focus, secondary sepsis, pathogen: grampositive bacterium, gramnegative bacterium, viral infection, culture negative, SARS-CoV-2, infection with multidrug resistant bacteria; comorbidities: chronic heart failure, immunosuppression, liver cirrhosis, chronic obstructive pulmonary disease, chronic hemodialysis, coronary artery disease, peripheral artery disase, stroke, dementia. *Abbreviations: CCI = Charlson Comorbidity Index, COPD = Chronic obstructive pulmonary disease, CAD = Coronary artery disease*

**Table S10. Outcome of the individual subgroups**

|  | n | In-hospital mortality | OR | 95% CI |
| --- | --- | --- | --- | --- |
| Early model |  |  |  |  |
| SG1 | 76 | 58 (76%) | 5.73 | 3.35, 10.2 |
| SG2 | 242 | 76 (31%) | 0.53 | 0.37, 0.73 |
| SG3 | 236 | 101 (43%) | 1.14 | 0.82, 1.58 |
| SG4 | 83 | 25 (30%) | 0.59 | 0.35, 0.95 |
| Late model | 2.03 |  |  |  |
| SG 1 | 91 | 60 (66%) | 5.15 | 3.17, 8.51 |
| SG2 | 203 | 44 (22%) | 0.32 | 0.21, 0.48 |
| SG3 | 152 | 53 (35%) | 0.98 | 0.65, 1.47 |
| Transitioned to other SG |  |  |  | Pseudo-R^2^ = 0.14 |
| To SG1 | 61 | 41 (67%) | 3.43 | 1.98, 6.13 |
| To SG2 | 42 | 10 (24%) | 0.44 | 0.2, 0.88 |
| To SG 3 | 76 | 30 (39%) | 0.96 | 0.58, 1.56 |

Categorical variables are presented as Frequency (%). *Abbreviations: SG = subgroup, OR = Odds ratio, CI = confidence interval*

**Figure S7. Original alluvial plots**

**A
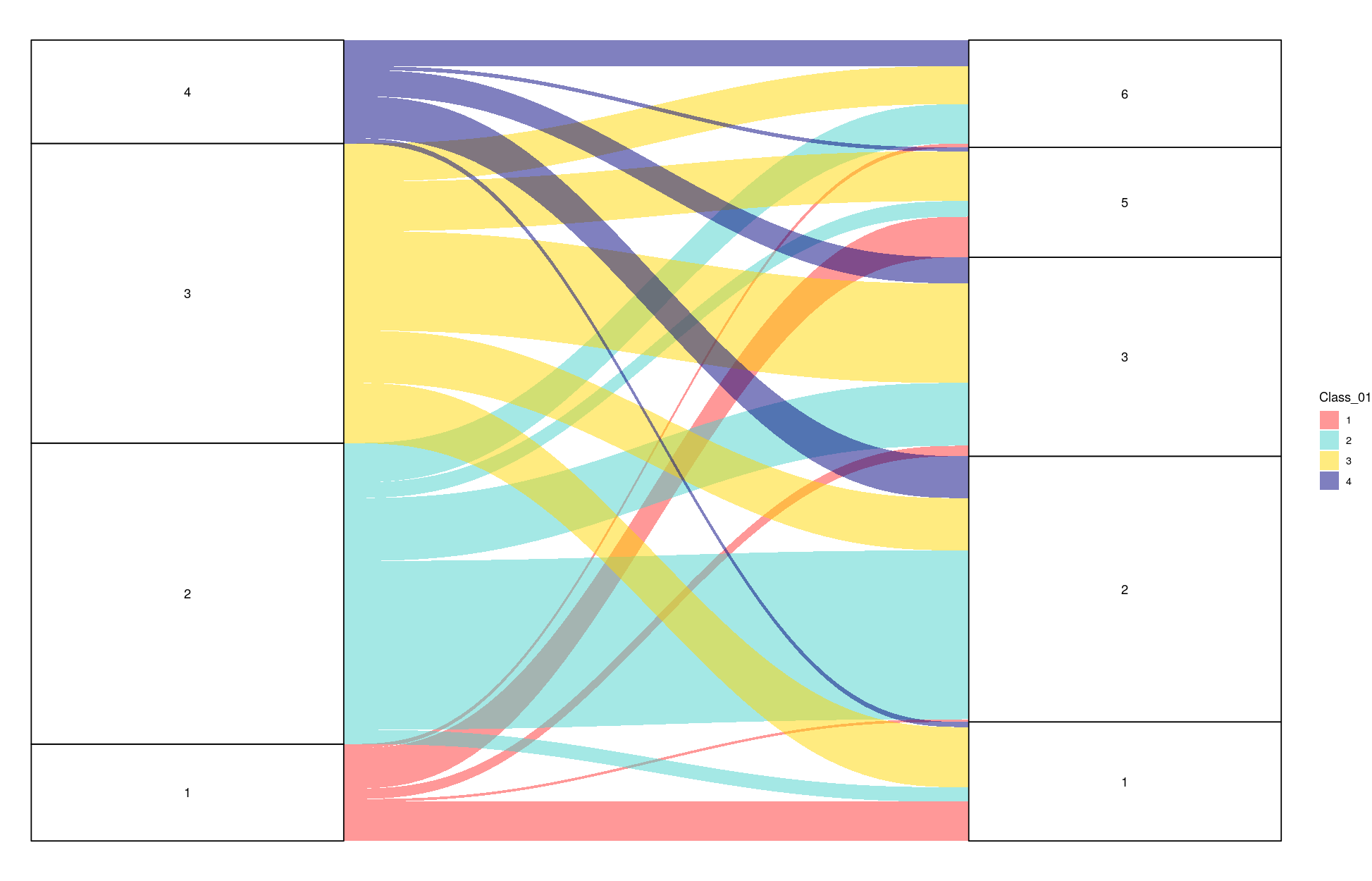
**

**B
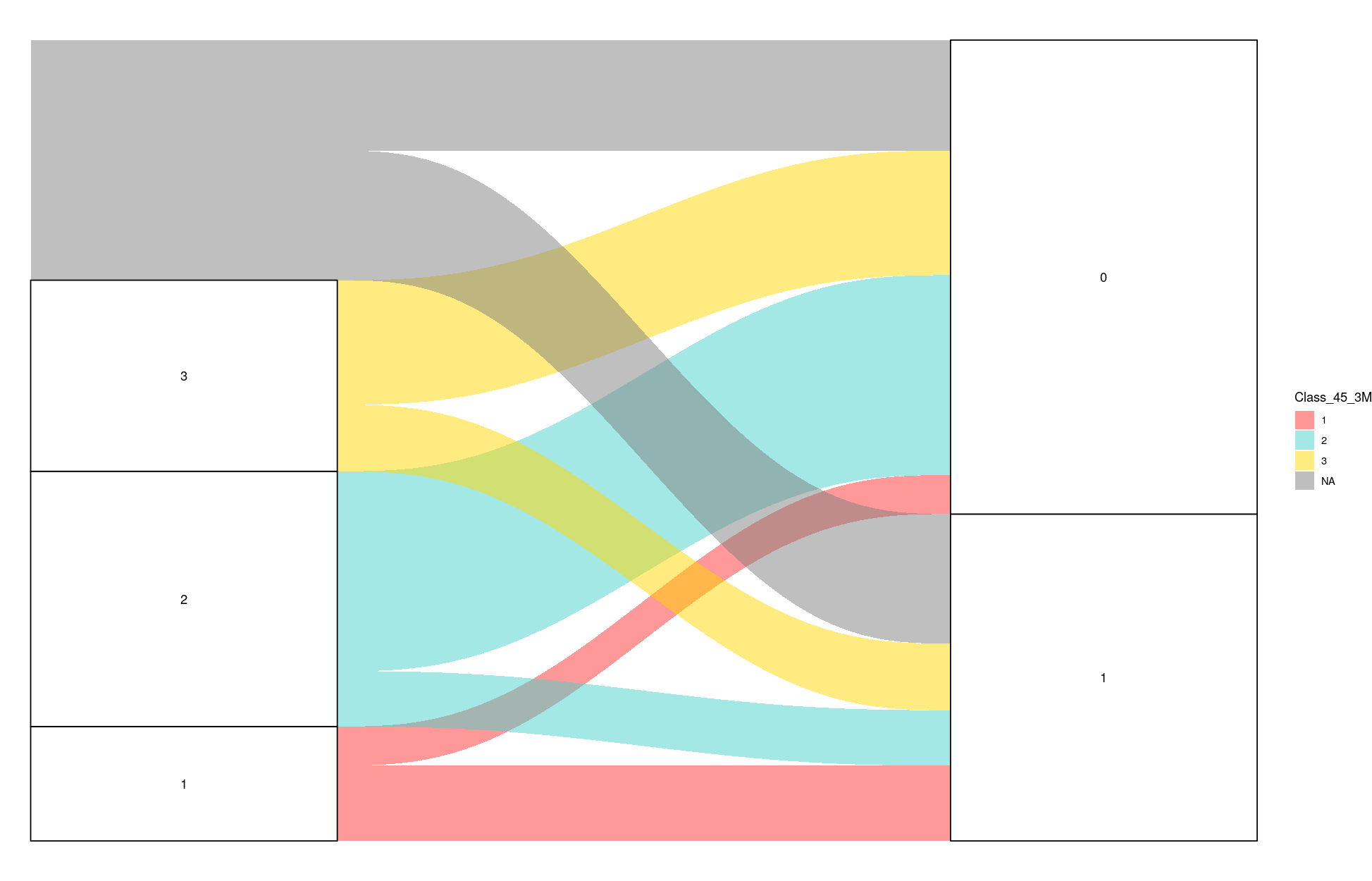
**
Alluvial plots as given by R Statistics before being manually combined into a composite figure (Figure 4). A: Classes 1-4 of the left column correspond to subgroups 1-4 of the early LPA. Classes 1-4 on the right column correspond to subgroups 1-4 of the late LPA, class 5 = already deceased at the late time point, class 6 = already transferred to the normal ward at the late time point. B: Classes 1-4 in the left column correspond to subgroups 1-4 of the late LPA. The grey bar at the top corresponds to deceased and transferred patients. The columns of the right bar correspond to in-hospital mortality, where 0 = discharged alive, 1 = died in hospital.

**Figure S8. Mediation analysis:**

**
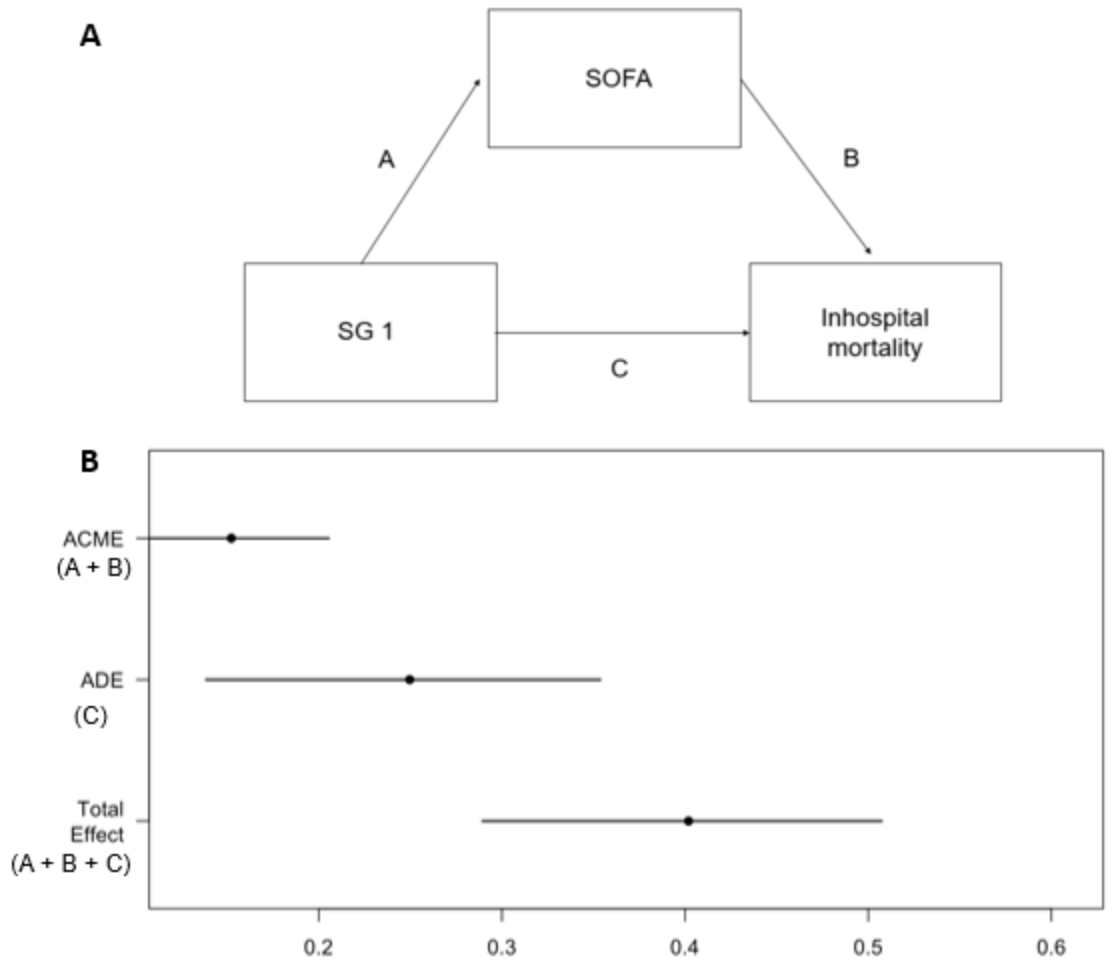
**

Mediation analysis. A: Visualization of the mediation analysis. B: Average mediated, direct and total effect on the association between SG1/early model and inhospital mortality, with the maximum SOFA score within 48 hours of inclusion as a potential mediator. Proportion mediated of the total effect on inhospital mortality by A+B: 37,6% (95% CI 25,7 – 55%). Abbreviations: ACME = average causal mediation effect (A +B), ADE = average direct effect (C), SG1 = subgroup 1 in the early model.

**Table S11A. External validation of the reduced model in the eICU database**

|  | **MHH_Sepsis** | **eICU** |
| --- | --- | --- |
|  | N = 637 | N = 508 |
| Age | 65 (54-74) | 64 (52, 76) |
| Gender | 420 (66%) | 292 (57%) |
| Hospital mortality | 260 (41%) | 107 (21%) |
| CRP (mg/l) | 208 (118-309) | 142 (52, 246) |
| WBC(x 1000/µl) | 16 (11-23) | 16 (11, 23) |
| Platelets (x 1000/µl) | 148 (94-229) | 147 (95, 204) |
| INR | 1.3 (1.1, 1.5) | 1.3 (1.1, 1.9) |
| ALT (U/l) | 35 (20, 78) | 31 (19, 64) |
| Bilirubin (µmol/l) | 14 (8, 31) | 15 (9, 26) |
| Creatinine (µmol/l) | 151 (97, 230) | 150 (95, 249) |
| Lactate (mmol/l) | 2.8 (1.7, 5.4) | 2.5 (1.5 – 4.3) |

Baseline characteristics of our cohort and the eICU cohort. ICU patients with a diagnosis of sepsis were included. If there were duplicates, only the primary case was included in the analysis. We also excluded all cases with missing values in class-defining variables with > 25% missing data: CRP, ALT, INR, and lactate. Variables included in the model were: CRP, WBC, platelets, INR, bilirubin, creatinine, and lactate. We obtained each the most pathological value in a timeframe of 48 hours of inclusion. No PCT and insufficient values for PaO2/FiO2 ratio and norepinephrine rate were obtained, resulting in the reduction of the model by these variables. Abbreviations: *CRP = C-reactive protein, WBC = white blood cell count, INR = international normalized ratio of prothrombin time, ALT = alanine aminotransferase.*

**Table S11B.** Model fit values of the latent profile analysis – eICU

|  | LL | AIC | BIC | Entropy | | Prob (min) | Prob (max) | N (Min) | N (Max) | BLRT(p) |
| --- | --- | --- | --- | --- | --- | --- | --- | --- | --- | --- |
| VVV- 1 Profile | 672 | -1235 | -1007 | | 1 | 1 | 1 | 1 | 1 |  |
| VVV- 2 Profiles | 1976 | -3735 | -3274 | | 0.94 | 0.98 | 0.99 | 0.36 | 0.64 | *.01* |
| VVV- 3 Profiles | 2220 | -4111 | -3418 | | 0.89 | 0.94 | 0.97 | 0.28 | 0.37 | *.01* |
| VVV- 4 Profiles | 2520 | -4601 | -3675 | | 0.90 | 0.93 | 0.96 | 0.13 | 0.36 | *.01* |
| VVV- 5 Profiles | 2581 | -4614 | -3455 | | 0.89 | 0.91 | 0.97 | 0.15 | 0.27 | *.218* |
| VVV- 6 Profiles | 2738 | -4819 | -3427 | | 0.89 | 0.91 | 0.97 | 0.11 | 0.26 | *.01* |

*Fit statistics suggest a model with four profiles. Abbreviations: VVV = varying variances and varying covariances, LL = Log Likelihood, AIC = Akaike Information criterion, BIC = Bayesian information criterion, N (min) = proportion of the sample assigned to the smallest profile, N(max) = proportion of the sample assigned to the largest profile. BLRT(p) = p-value for the bootstrapped likelihood ratio test, SALPP = smallest average latent profile posterior probability, Prob(Min): Minimal average latent class probability for most likely class membership. Prob(Max) Maximal average latent class probability for most likely class membership*

**Table S11C.** Model fit values of the latent profile analysis – reduced model: own cohort

|  | LL | AIC | BIC | Entropy | | Prob (min) | Prob (max) | N (Min) | N (Max) | BLRT(p) |
| --- | --- | --- | --- | --- | --- | --- | --- | --- | --- | --- |
| VVV- 1 Profile | 2187 | -4266 | -4026 | | 1 | 1 | 1 | 1 | 1 |  |
| VVV- 2 Profiles | 3724 | -7229 | -6743 | | 0.93 | 0.96 | 0.99 | 0.31 | 0.67 | *.01* |
| VVV- 3 Profiles | 4026 | -7723 | -6992 | | 0.85 | 0.89 | 0.96 | 0.23 | 0.45 | *.01* |
| VVV- 4 Profiles | 4262 | -8085 | -7109 | | 0.87 | 0.89 | 0.97 | 0.18 | 0.37 | *.01* |
| VVV- 5 Profiles | 4389 | -8231 | -7010 | | 0.87 | 0.90 | 0.96 | 0.12 | 0.29 | *.01* |
| VVV- 6 Profiles | 4473 | -8287 | -6821 | | 0.85 | 0.84 | 0.95 | 0.11 | 0.27 | *.01* |

*Fit statistics suggest a model of four profiles. Abbreviations: VVV = varying variances and varying covariances, LL = Log Likelihood, AIC = Akaike Information criterion, BIC = Bayesian information criterion, N (min) = proportion of the sample assigned to the smallest profile, N(max) = proportion of the sample assigned to the largest profile. BLRT(p) = p-value for the bootstrapped likelihood ratio test, SALPP = smallest average latent profile posterior probability, Prob(Min): Minimal average latent class probability for most likely class membership. Prob(Max) Maximal average latent class probability for most likely class membership*

**Table S11D. Subgroup characteristics of the reduced models: own cohort and eICU**

|  | **Own cohort** |  |  |  |  | **eICU** |  |  |  |  |
| --- | --- | --- | --- | --- | --- | --- | --- | --- | --- | --- |
|  | SG1  N = 105 | SG2  N = 183 | SG3  N = 236 | SG4  N =  113 | p^1^ | SG1  N = 58 | SG2  N = 137 | SG3  N = 116 | SG4  N = 197 | P^1^ |
| age | 62 (51, 71) | 63 (52, 73) | 67 (57, 75) | 68 (55, 78) | .002 | 63 (53, 73) | 64 (53, 74) | 69 (56, 80) | 63 (51, 76) | .005 |
| Male gender | 65 (62%) | 132 (72%) | 144 (61%) | 79 (70%) | .40 | 37 (64%) | 86 (63%) | 66 (57%) | 103 (52%) | .02 |
| Hospital mortality | 67 (64%) | 61 (33%) | 74 (31%) | 58 (51%) | <.001 | 23 (40%) | 30 (22%) | 34 (30%) | 20 (10%) | <.001 |
| CRP (mg/l) | 146 (95, 247) | 226 (134, 325) | 219 (134, 314) | 232 (104, 310) | <.001 | 134 (56, 200) | 205 (82, 338) | 107 (54, 213) | 155 (39, 231) | <.001 |
| WBC (x1000/µl) | 19 (13, 26) | 15 (10, 21) | 14 (9, 18) | 21 (12, 26) | <.001 | 17 (11, 24) | 15 (10, 22) | 20 (11, 27) | 16 (11, 21) | .20 |
| PCT (µg/l) | 9 (2, 32) | 4 (1, 22) | 3 (1, 11) | 12 (3, 72) | <.001 |  |  |  |  |  |
| Platelets (x1000/µl) | 109 (60, 160) | 147 (86, 230) | 173 (120, 250) | 144 (92, 210) | <.001 | 130 (60, 193) | 136 (90, 193) | 118 (67, 167) | 176 (125, 233) | <.001 |
| INR | 1.9 (1.4, 2.9) | 1.3 (1.1, 1.5) | 1.2 (1.1, 1.3) | 1.3 (1.2, 1.6) | <.001 | 1.88 (1.3 – 3.63) | 1.3 (1.1 – 1.58) | 2.8 (1.93 – 4.1) | 1.19 (1.1 – 1.3) | <.001 |
| ALT (U/l) | 156 (38, 830) | 44 (26, 68) | 20 (15, 29) | 81 (40, 157) | <.001 | 403 (136, 1365) | 55 (25-93) | 26 (16, 39) | 23 (17, 35) | <.001 |
| Bilirubin (µmol/l) | 51 (21,  98) | 29 (14, 49) | 9 (6, 13) | 13 (8, 19) | <.001 | 43 (18, 95) | 14 (9, 22) | 24 (12, 51) | 12 (9, 17) | <.001 |
| Creatinine (µmol/l) | 179 (132, 262) | 125 (79, 179) | 129 (89, 201) | 229 (141, 400) | <.001 | 165 (95, 248) | 279 (156, 495) | 161 (106, 278) | 106 (73, 156) | <.001 |
| Lactate (mmol/l) | 7.3 (3.1, 12.1) | 2.0 (1.5, 2.9) | 2.4 (1.6, 3.9) | 6.1 (2.8, 9.9) | <.001 | 5 (2, 10.3) | 2.6 (1.5, 4.7) | 2.7 (1.8, 4.8) | 2 (1.3 – 3.0) | <.001 |

Both cohorts have a subgroup characterized by hepatobiliary dysfunction, high lactate, and higher in-hospital mortality (own cohort: SG1, eICU: SG1). The subgroup with the second highest mortality has the highest WBC count in both cohorts (own cohort: SG4, eICU: SG3).^1^tested with Wilcoxon rank sum test, Fisher’s exact test or Wilcoxon rank sum exact test. Abbreviations: *CRP = C-reactive protein, WBC = white blood cell count, PCT = procalcitonin, INR = international normalized ratio of prothrombin time, ALT = alanine aminotransferase.*

**Table S11E. Multivariate regression analysis: Predictors for subgroup assignment in the eICU cohort**

| SG1 | OR | 95% CI | p-value |
| --- | --- | --- | --- |
| Lactate_max | 1.85 | 1.03, 3.38 | .038 |
| Platelets | 3.5 | 1.86, 6.96 | <.001 |
| INR_max | 2.07 | 1.19, 3.59 | .008 |
| Creatinine_max | 0.28 | 0.09, 0.66 | .001 |
| Bilirubin_max | 6.36 | 3.35, 14.6 | <.001 |
| ALT | 9,462,693 | 57,425, 9,510,130,476 | <.001 |

Nagelkerke R-square: .87

| SG2 | OR | 95% CI | p-value |
| --- | --- | --- | --- |
| Lactate_max | 2.12 | 1.49, 3.06 | <.001 |
| Platelets | 0.52 | 0.35, 0.77 | .001 |
| INR_max | 0.08 | 0.02, 0.21 | <.001 |
| Creatinine_max | 6.87 | 4.43, 11.3 | <.001 |
| Bilirubin_max | 0.22 | 0.1, 0.45 | <.001 |
| ALT | 1.03 | 1.02, 1.04 | <.001 |
| CRP | 36.1 | 11, 133 | <.001 |

Nagelkerke R-square: .62

| SG3 | OR | 95% CI | p-value |
| --- | --- | --- | --- |
| Platelets | 0.51 | 0.3, 0.8 | .006 |
| INR_max | 37.5 | 16.4, 99.0 | <.001 |
| WBC_max | 2.01 | 1.26, 3.49 | .009 |
| Bilirubin_max | 2.39 | 1.49, 3.86 | <.001 |
| ALT | 0.00 | 0.00, 0.00 | <.001 |
| CRP | 0.01 | 0.00, 0.1 | <.001 |

Nagelkerke R-square: .76

| SG4 | OR | 95% CI | p-value |
| --- | --- | --- | --- |
| Lactate_max | 0.26 | 0.21, 0.5 | <.001 |
| INR_max | 0.0 | 0.00, 0.01 | <.001 |
| Creatinine_max | 0.05 | 0.02, 0.11 | <.001 |
| Bilirubin_max | 0.02 | 0.00, 0.16 | <.001 |
| ALT | 0.00 | 0.00, 0.00 | <.001 |
| CRP | 0.06 | 0.01, 0.35 | .003 |

Nagelkerke R-square: .85

Multivariate logistic regression analysis with forward variable selection. Variables included in the model were: Age, CRP, WBC, platelets, INR, bilirubin, ALT, creatinine and lactate. In each case, the most pathological value in a 48-hour period was used. *Abbreviations: CRP = C-reactive protein, WBC = white blood cells, , INR = international normalized ratio of prothrombin time, ALT = alanine aminotransferase.*

**Table S11F. Multivariate regression analysis: Risk factors for subgroup assignment in the reduced model of our own cohort**

| SG1 | OR | 95% CI | p-value |
| --- | --- | --- | --- |
| Lactate_max | 1.96 | 1.34, 2.9 | <.001 |
| INR_max | 6.04 | 3.52, 11.4 | <.001 |
| Bilirubin_max | 6.25 | 3.71, 11.4 | <.001 |
| ALT | 179 | 34, 1,410 | <.001 |

Nagelkerke R-square: .80

| SG2 | OR | 95% CI | p-value |
| --- | --- | --- | --- |
| Female sex | 0.6 | 0.38, 0.92 | .021 |
| Creatinine_max | 0.55 | 0.41, 0.71 | <.001 |
| Bilirubin_max | 1.36 | 1.1, 1.72 | .006 |
| Lactate_max | 0.24 | 0.15, 0.37 | <.001 |

Nagelkerke R-square: .37

| SG3 | OR | 95% CI | p-value |
| --- | --- | --- | --- |
| INR_max | 0.14 | 0.06, 0.28 | <.001 |
| Creatinine_max | 0.61 | 0.45, 0.83 | .001 |
| Bilirubin_max | 0.00 | 0.00, 0.00 | <.001 |
| ALT | 0.00 | 0.00, 0.00 | <.001 |
| CRP | 1.56 | 1.13, 2.18 | .007 |

Nagelkerke R-square: .80

| SG4 | OR | 95% CI | p-value |
| --- | --- | --- | --- |
| WBC_max | 2.33 | 1.75, 3.19 | <.001 |
| Lactate_max | 4.47 | 3.17, 6.53 | <.001 |
| INR_max | 0.56 | 0.35, 0.81 | .007 |
| Creatinine_max | 2.85 | 2.15, 3.87 | <.001 |
| Bilirubin_max | 0.02 | 0.00, 0.08 | <.001 |
| ALT | 0.28 | 0.12, 0.53 | <.001 |
| Platelets | 0.58 | 0.4, 0.81 | .002 |

Nagelkerke R-square: .57

Multivariate logistic regression analysis with forward variable selection. Variables included in the model were: Age, CRP, WBC, platelets, INR, bilirubin, ALT, creatinine and lactate. In each case, the most pathological value in a 48-hour period was used. *Abbreviations: CRP = C-reactive protein, WBC = white blood cells, INR = international normalized ratio of prothrombin time, ALT = alanine aminotransferase.*
